# Effectiveness of Videoconference-Delivered Cognitive Behavioral Therapy for Adults With Psychiatric Disorders: Systematic and Meta-Analytic Review

**DOI:** 10.2196/31293

**Published:** 2021-12-13

**Authors:** Kazuki Matsumoto, Sayo Hamatani, Eiji Shimizu

**Affiliations:** 1 Research Center for Child Mental Development Chiba University Chiba Japan; 2 Laboratory of Neuropsychology, Institute of Liberal Arts and Science Kanazawa University Kanazawa Japan; 3 Department of Behavioural Sciences and Learning Linköping University Linköping Sweden; 4 Research Center for Child Mental Development University of Fukui Fukui Japan; 5 Department of Cognitive Behavioral Physiology Graduate School of Medicine Chiba University Chiba Japan; 6 Cognitive Behavioral Therapy Center Chiba University Hospital Chiba Japan

**Keywords:** videoconference-delivered cognitive behavioral therapy, depression, anxiety, psychiatric disorders, systematic review, meta-analysis, digital health, mental health, cognitive therapy, internet-based therapy, cognition, neurodevelopment, communication technology, health technology, psychological disorders, anxiety disorder

## Abstract

**Background:**

Cognitive behavioral therapy (CBT) is the gold standard of psychotherapy for psychiatric disorders. However, the format of delivering CBT in person limits access to the intervention. The advancements in information and communication technology, especially the internet, present an opportunity for cognitive behavioral therapists to service patients or clients in remote areas through videoconferencing. Although many randomized controlled trials of videoconference-delivered cognitive behavioral therapy (VCBT) have already been conducted, the overall estimated effect size of VCBT for psychiatric disorders has not been examined by systematic reviews and meta-analyses.

**Objective:**

This study attempts to evaluate the effectiveness of VCBT for psychiatric disorders through a systematic and meta-analytic review.

**Methods:**

A systematic review and meta-analysis of studies in which VCBT was directly compared to control groups (such as treatment as usual, attention control, wait-list control, and other minimal supports) was carried out. To identify previous studies that meet our study objective, 2 independent reviewers undertook a systematic search through seven databases: MEDLINE (via PubMed), Web of Science, Science Direct, PsycINFO, CINAHL, LILACS, and SciELO. Other databases (ClinicalTrials.gov and Cochrane Central Resister of Controlled Trials) were also checked. All studies included in the review were assessed using the quality criteria of the Cochrane Collaboration. Statistical analysis was performed by using Cochrane Review Manager (RevMan, version 5.4.0). Standardized mean difference was used in major meta-analyses where a *P* value of .05 or less was the threshold for statistical significance. A heterogeneity test and the chi-square test were performed to assess the presence and extent of statistical heterogeneity with significance set at *P*<.10. Funnel plots were visually inspected to assess the risk of bias. Subgroup analyses were conducted for each disorder to estimate intervention effects.

**Results:**

The systematic search resulted in 16 studies (total N=1745) that met the criteria for this study and were included in the review. There were 10 studies on depressive symptoms, 3 on chronic pain, 1 on generalized anxiety disorder, 1 on obsessive-compulsive disorder, and 1 on hypochondriasis. The quality and risk of bias was also assessed. Results showed a pooled effect size (Hedge *g*) post treatment of −0.49 (95% CI –0.68 to –0.29), indicating that VCBT is effective for clients with psychiatric disorders. Study quality did not affect outcomes.

**Conclusions:**

While the overall results indicate the effectiveness of VCBT, there are still only a limited number of studies on specific psychiatric and somatic conditions. Therefore, more randomized controlled trials are needed to establish the effectiveness of VCBT for different disorders.

**Trial Registration:**

International Prospective Register of Systematic Reviews (PROSPERO) CRD42021224832; https://www.crd.york.ac.uk/prospero/display_record.php?RecordID=224832

## Introduction

### Background

The incidence of mental health disorders has significant socioeconomic implications for public health and human rights globally. Depression, for example, is a leading cause of disability, affecting an estimated 264 million people worldwide [1]. It has been shown that cognitive behavioral therapy (CBT) is an effective treatment for a variety of mental disorders [2,3]. Cognitive behavioral therapists analyze the effects of their patients’ cognition and behavior on psychiatric symptoms [4,5], and work toward developing adaptive cognitive-behavioral techniques with their client [6,7]. Evidence suggests that CBT is effective not only for psychiatric disorders [8] but also for somatic disorders [9]. It is considered the gold standard in the treatment of mental health disorders because it is substantiated by theory and research [10]. Further, evidence suggests that CBT is superior to other modalities, such as interpersonal psychotherapy [11]. The World Health Organization (2019) has also recognized its effectiveness and stated that access to CBT is important [12]. Face-to-face therapy is the most common format for providing treatment for mental health issues. However, this can restrict access to CBT for patients living in remote areas. Considering the widespread use of the internet and telecommunications equipment [13], there is a window of opportunity to provide access to CBT to patients living in remote areas [14]. Most remote CBT is delivered with the help of websites/webpages, under the guidance of a therapist. This format is called internet-based cognitive behavioral therapy (ICBT) or simply “internet intervention.” According to Olthuis et al [15], “to be considered an Internet intervention, CBT must have been delivered over the Internet through the use of web pages or e-mail, or both.” Two systematic reviews including meta-analyses have suggested that in terms of effectiveness, ICBT is equivalent to face-to-face CBT [16,17].

### Videoconference-Delivered CBT

Another approach to improve accessibility to CBT for individuals residing in remote areas is to utilize a videoconferencing system [18-20]. In comparison to ICBT, videoconference-delivered CBT (VCBT) has the advantage of enabling remote treatment through interactive real-time communication between the therapist and the patient, which makes it similar to face-to-face CBT. At the same time, VCBT differs from face-to-face CBT because there is a “physical separation” between the therapist and patient, which may create limitations in clinical practice. For example, when dealing with a patient with obsessive-compulsive disorder, the patient cannot directly touch the stimulus/subject provided by the therapist. The patient must work on the subject at home on their own. Time lags and poor eye contact during video calls may affect the quality of interaction between the therapist and patient, creating obstructions for cognitive reconstruction and the creation of cognitive models.

Despite these concerns, results from previous clinical trials investigating the feasibility and efficacy of VCBT were generally promising [18-21]. The results from prospective and rigorous clinical trials (RCTs) have suggested that VCBT is not inferior to face-to-face CBT for the treatment of depression and posttraumatic stress disorder [22-25]. An existing literature review summarized previous findings [26] but did not conduct a meta-analysis of the results of RCTs drawing direct comparison to controls such as conventional treatment. A network meta-analysis that examined the most effective CBT format for the treatment of acute depression also did not compare for VCBT [27]. Several systematic reviews to assess the effectiveness of videoconference-delivered psychotherapy for the treatment of depression [28] and anxiety disorders [29] suggest that VCBT is an acceptable form of remote therapy for such patients, and clinical symptoms can be expected to improve. However, these reviews did not perform a meta-analysis owing to a lack of RCTs. Therefore, the estimate effect size of VCBT could not be gauged.

### Study Objective

As of December 2020, results from new RCTs to validate the effectiveness of VCBT for people with depressive symptoms [30-35], chronic pain, and hypochondriasis [36,37] have emerged. Therefore, there is a need for a systematic review and meta-analysis focusing on VCBT. The objective of this study is to examine the effectiveness of VCBT as a treatment option for psychiatric and somatic disorders in comparison to control conditions. The population of this review targeted both clinical and community samples. To increase the credibility of the results by reviewing high-evidence studies [38], this review included only RCTs. This study’s protocol was registered with PROSERO (CRD42021224832) [39]. The protocol planned to include RCTs targeting children and adolescents. However, owing to a small number of RCTs with children and adolescents as participants [40,41], the selection criteria for this review focused on studies with adult participants. This review was in accordance with the Preferred Reporting Items for Systematic reviews and Meta-Analyses (PRISMA) 2020 statement [42], see the PRISMA checklist provided in Multimedia Appendix 1.

## Methods

### Eligibility Criteria

The eligibility criteria for the original studies to be a part of review were as follows: (1) subjects of the study were adults (age>18 years); (2) the intervention was VCBT; (3) the intervention group was compared to attention training (AT), treatment as usual (TAU), wait-list control (WLC), or other active control (AC) conditions; (4) the outcome was the effect of VCBT on the management of the symptoms of psychiatric or somatic disorders (primary outcome measures); (5) used a randomized control study design; and (6) were written in English. Exclusion criteria were studies in which the intervention was not based on cognitive-behavioral techniques, participants were children or adolescents, and the necessary data were inaccessible.

### Information Sources and Search Strategy

To identify previous studies that met our study objective, systematic searches were conducted on MEDLINE (via PubMed), Web of Science, Science Direct, PsycINFO, CINAHL, LILACS, and SciELO using the following terms related to psychiatric and somatic disorders: “depression,” “panic disorder,” “social phobia,” “social anxiety disorder,” “generalized anxiety disorder,” “obsessive-compulsive disorder,” “post-traumatic stress disorder,” “specific phobia,” “hypochondriasis,” “bulimia,” “tinnitus,” “erectile dysfunction,” “chronic pain,” or “fatigue.” To determine the intervention approach, these search terms were combined with “videoconference,” “video conference,” “videoconferencing,” “tele,” “teleconference,” “tele conference,” or “teleconferencing,” and the search filter “randomized controlled trial” was used. The search did not include unpublished studies. Searches were last updated on December 25, 2020. Other databases were also checked (ClinicalTrials.gov and Cochrane Central Resister of Controlled Trials), along with the references of the previous systematic reviews [28,29]. For more information on the full search strategies, see the complete search strategy provided in Multimedia Appendix 2.

### Process of Selection and Data Collection

A total of 2 reviewers (KM and SH) independently made decisions on whether they met the selection criteria in accordance with the aforementioned search strategy. If the selected studies did not match, the decision was made by a joint discussion among the research team, including a third party (ES). The selected studies were managed using EndNote.

### Data Items

Participants were those who received VCBT without restrictions, including clinical samples, community members, and students. We set the clinical symptoms as outcomes before and after the intervention. For example, if satisfaction or acceptance of the intervention was the primary outcome, a secondary outcome to measure the severity of the target disorder was adopted. The intervention was conditional on the inclusion of having sessions with the therapist via a videoconferencing system and cognitive behavioral techniques such as behavioral activation, cognitive restructuring, exposure, and mindfulness, among others.

### Study Risk of Bias Assessment

The first and second authors (KM and SH) read the abstracts independently. In case of any disagreement regarding the inclusion of a particular study, the article was discussed among all researchers. All studies included in the review were assessed using the revised Cochrane risk-of-bias tool for randomized trials (RoB 2) [43,44]. The included studies were rated on each of the aforementioned dimensions as low risk, some concern, or high risk.

### Statistical Analysis

#### Effect Measures

The standardized mean difference (SMD) was used in major meta-analyses because the studies included in this review used different symptom evaluation scales for the primary outcomes. The value of SMD depends on the effect size (difference in mean) and SD of the outcome (unique variation between participants). In the event of a missing summary statistic, we contacted the authors. If there was no reply, it was excluded from this review because there were no data that can be handled.

#### Synthesis Methods

Statistical analysis was performed by using Cochrane Review Manager (RevMan; version 5.4.0) [43]. First, standardization was achieved by dividing the mean difference (the change from baseline to the end of the study or the value at the end of the study) by the SD of the control group in the study. Next, in a meta-analysis, the standardized mean values from individual studies were integrated to calculate the SMD. The data reflecting intention-to-treat took precedence over the per-protocol data in the meta-analysis. Intervention effects were assessed with random-effect meta-analyses, assuming variation in true effects and accounting for the hypothesized effect distribution [45,46]. A *P* value of ≤.05 was considered the threshold for statistical significance. According to the power calculation by Borenstein et al [45], a power of 80% to detect a small effect size required that each group has an average of 25 participants and studies be 14 or more in number (if the probability of rejecting the null hypothesis is 5%). Subgroup analyses to the estimation of intervention effects were conducted for each disorder.

The heterogeneity test and the chi-square test were performed. Significant heterogeneity of >40% suggests the presence of heterogeneity [47]. The presence and quantity of statistical heterogeneity was assessed using the *I*² statistic, with significance set at *P*<.10 [48].

Sensitivity analysis was performed to compare studies that were judged to have a low risk of bias and to determine the quality of the affected outcomes.

### Reporting Bias

The funnel plots were visually inspected to assess the risk of bias.

## Results

### Studies Included in the Review

**Figure 1:**
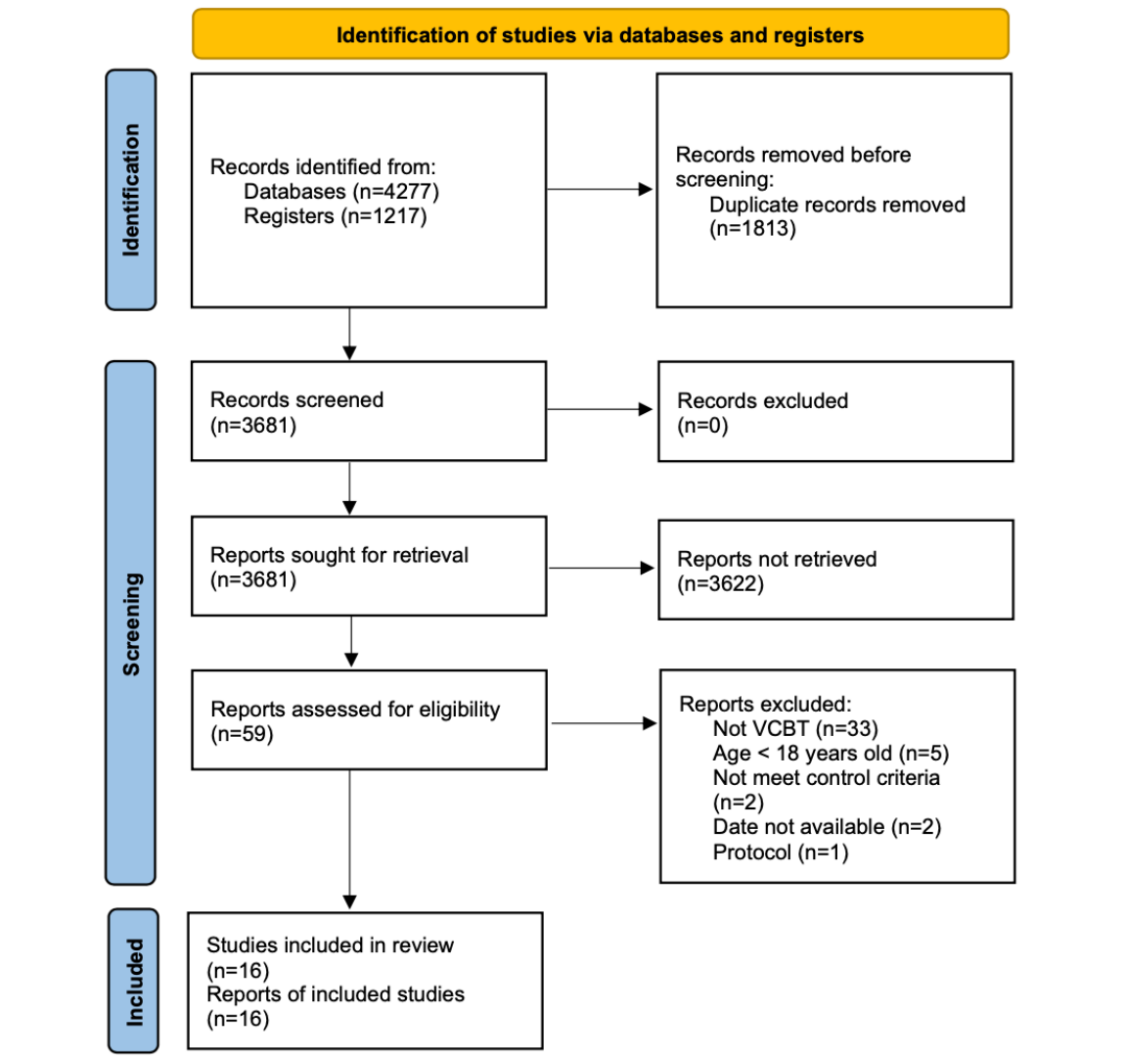
The inclusion process. VCBT: Videoconference-Delivered Cognitive Behavioral Therapy.

Of the 3684 screened studies, 16 (N=1745) met all selection criteria and were included in the analysis. Figure 1 shows the inclusion process based on the PRISMA (Preferred Reporting Items for Systematic Reviews and Meta-Analyses) 2020 flow diagram [49]. There was a 3-arm RCT [33]; 2 arms of those intervention groups were behavioral activation and problem-solving. In terms of participants’ conditions, 10 studies targeted depressive symptoms [30,32-35,50-54], 3 focused on chronic pain [31,37,55], 1 on obsessive-compulsive disorder [21], 1 on general anxiety disorder [56], and 1 on hypochondriasis [36]. The total number of participants from whom posttreatment data were collected was 768 in VCBT and 718 in the control conditions. Studies that reported 2 RCTs were excluded because no data were available [57,58].

### Characteristics of the Selected Studies

The RCTs included in this review were conducted by 13 independent research teams. Of them, 11 were performed in the United States, 2 in the United Kingdom, 2 in Canada, and 1 in Norway. The smallest study had 27 participants and the largest had 343 participants. All studies were published between 2008 and 2020. One RCT was configured with 2 VCBT intervention arms [33], the sample size of the control group set to half when the effect size was estimated in the meta-analysis. Table 1 presents the characteristics of each included study (see Multimedia Appendix 2 for details).

**Table 1:** Characteristics of the selected studies.

Selected studies	Diseases	Intervention			Participants, n			Outcomes		VCBT^a^, mean (SD)			Control, mean (SD)			Quality		Sampling
		CBT^b^ technique	Control	VCBT		Control			Pre		Post	Pre		Post				
Ahmad (2020) [30]	Depression	Mindfulness	WLC^c^	40		40	PHQ-9^d^		8.1 (6.0)		6.0 (3.9)	9.1 (6.2)		9.7 (6.9)	Low risk		Community	
Alschuler (2021) [31]	Pain	CBT	TAU^e^	15		12	PCS^f^		20.1 (7.8)		15.6 (10.0)	17.4 (10.2)		17.3 (9.3)	Some concerns		Clinical	
Bogosian (2015) [50]	Depression	Mindfulness	WLC	19		21	Depression HADS^g^		6.2 (3.5)		5.1 (3.2)	7.2 (3.4)		7.6 (4.0)	Low risk		Clinical	
Choi (2014) [51]	Depression	PS^h^	Telephone support call	43		36	HAMD^i^		24.6 (6.6)		13.9 (7.7)	24.6 (6.6)		19.2 (7.8)	Some concerns		Community	
Choi (2020a) [32]	Depression	Behavioral activation	Tele friendly visits	43		46	PHQ-9		7.2 (4.0)		5.9 (3.8)	7.7 (4.5)		8.3 (4.9)	Some concerns		Community	
Choi (2020b) [33]	Depression	Behavioral activation	AC^j^	99		49	HAMD		23.2 (5.7)		14.6 (9.5)	22.9 (5.7)		18.1 (10.7)	Low risk		Community	
Choi (2020b) [33]	Depression	PS	AC	98		49	HAMD		22.7 (5.7)		12.4 (10.6)	22.9 (5.7)		18.1 (10.7)	Low risk		Community	
Demiris (2019) [56]	GAD	PS	AC	171		172	GAD-7^k^		6.8 (5.3)		6.2 (4.6)	7.6 (5.2)		6.6 (4.9)	High risk		Community	
Elliott (2008) [53]	Depression	PS	Education only	21		14	IDD^l^		11.4 (9.4)		6.1 (6.6)	4.8 (6.1)		8.8 (13.5)	Some concerns		Community	
Ferguson (2016) [54]	Depression	CBT	Supportive therapy	27		20	DASS^m^ depression		6.0 (6.6)		3.7 (4.3)	12.6 (9.4)		7.3 (7.7)	Some concerns		Clinical	
EI-Jawahri (2020) [52]	Depression	CBT	TAU	45		47	HADSD		4.9 (2.8)		2.9 (5.6)	3.5 (3.4)		4.4 (5.5)	Some concerns		Clinical	
Fox (2020) [34]	Depression	CBSM^n^	AC	95		97	PROMIS^o^ depression		49.0 (7.3)		46.6 (9.2)	48.5 (7.4)		46.6 (8.1)	Some concerns		Clinical	
EI-Morr (2020) [35]	Depression	Mindfulness	WLC	80		80	PHQ-9		8.4 (5.6)		7.0 (5.0)	9.9 (6.2)		11.2 (6.7)	Low risk		Community	
Morriss (2019) [36]	Hypochondriasis	CBT	TAU	78		78	SHAI^p^		24.9 (4.2)		17.7 (8.0)	25.1 (4.5)		22.6 (6.8)	Some concerns		Clinical	
Somers (2018) [55]	Pain	PCST^q^	TAU	18		18	Pain severity		3.0 (2.1)		3.3 (2.4)	2.7 (1.9)		2.5 (1.9)	Some concerns		Clinical	
Vogel (2014) [21]	OCD^r^	ERP^s^	WLC	10		10	Y-BOCS^t^		24.2 (4.3)		11.5 (4.8)	23.4 (2.8)		23.4 (4.8)	High risk		Clinical	
Vranceanu (2019) [37]	Pain	TOR^u^	TAU	25		29	Physical function in SMFA^w^		69.8 (18.2)		20.7 (17.4)	63.2 (17.4)		48.6 (21.8)	Some concerns		Clinical	

^a^VCBT: Videoconference-Delivered Cognitive Behavioral Therapy

^b^CBT: Cognitive Behavioral Therapy

^c^WLC: Wait-List Control

^d^PHQ-9: Patient Health Questionnaire, 9-item

^e^TAU: Treatment As Usual

^f^PCS: Pain Catastrophizing Scale

^g^HADS: Hospital Anxiety and Depression Scale

^h^PS: Problem Solving

^i^HAMD: Hamilton Depression Rating Scale

^j^AC: Attention Control

^k^GAD-7: Generalized Anxiety Disorder, 7-item

^l^IDD: Inventory to Diagnose Depression

^m^DASS: Depression Anxiety Stress Scales

^n^CBSM: Cognitive-Behavioral Stress Management

^o^PROMIS: Patient-Reported Outcome Measurement Information System

^p^SHAI: Short Health Anxiety Inventory

^q^PSCBT: Problem-Solving Cognitive Behavioral Therapy

^r^OCD: Obsessive-Compulsive Disorder

^s^ERP: Exposure Response Prevention

^t^Y-BOCS: Yale-Brown Obsessive-Compulsive Scale

^u^TOR: Toolkit for Optimal Recovery

^w^SMFA: Short Musculoskeletal Function Assessment Questionnaire

### Risk of Bias in Studies

Evaluation of the quality of the studies included in this review shows that 4 RCTs (5 comparisons) are at a lower risk of bias [30,33,35,50], 10 RCTs are of some concern [31,32,34,36,37,51-55], and 2 RCTs are at a high risk of bias (see Multimedia Appendix 2 for details) [21,56].

### Results of Syntheses

**Figure 2:**
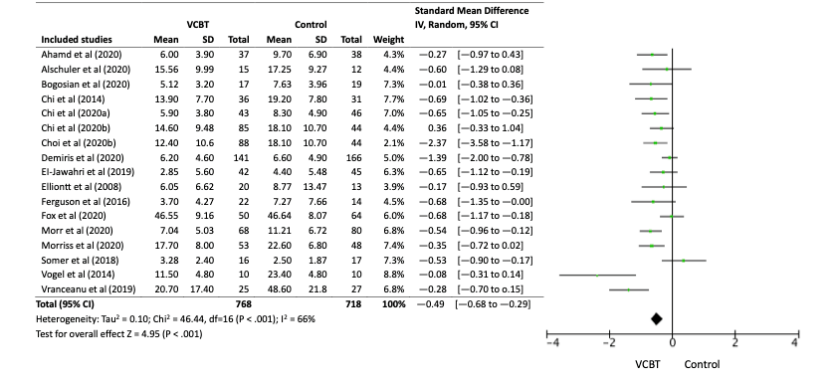
Forest plot. VCBT: videoconference-delivered cognitive behavioral therapy.

Figure 2, a forest plot, shows the effect size (Hedge *g*) of each study and the overall effect size integrated by the meta-analysis. An effect size estimated below 0 favors guided VCBT. In all 16 studies (17 intervention arms), the pooled between-group effect size (Hedge *g*) was −0.49 (95% CI −0.68 to −0.29, *P*<.001), showing that VCBT was significantly more effective than the control conditions.

In the 10 studies (11 comparing) focused on depressive symptoms, the effect size (Hedge *g*) was medium, at −0.46 (95% CI −0.60 to −0.32, *P*<.001). In 3 studies targeting chronic pain, the effect size (Hedge *g*) was −0.41 (95% CI −1.49 to 0.67, *P*=.46), showing the effectiveness of VCBT, but was not significant. In the study targeting generalized anxiety disorder, the effect size (Hedge *g*) was −0.08 (95% CI −0.31 to 0.14, *P*=.46). In the study targeting obsessive-compulsive disorder, the effect size (Hedge *g*) was −2.37 (95% CI −3.58 to −1.17, *P*<.001). In the study targeting hypochondriasis, the effect size (Hedge *g*) was −0.65 (95% CI −1.05 to −0.25, *P*=.001).

### Results of the Heterogeneity Test

Tests of heterogeneity demonstrated significant differences in effects across treatments (*τ*^2^=0.10; *χ*^2^_16_=46.40; *I*^2^=66%; *P*<.001). The heterogeneity was largely driven by a study conducted on exposure response prevention for obsessive-compulsive disorder [21], and 2 studies that conducted unique interventions [37,55]. If those studies were excluded, the *I*^2^ decreased from 66% to 27%: heterogeneity was not significant (*τ*^2^=0.02; *χ*^2^_13_=17.87; *P*=.16). The pooled effect size across all studies changed marginally from Hedge *g*=−0.47 (95% CI −0.69 to −0.23) to Hedge *g*=−0.44 (95% CI −0.59 to −0.29), if those studies were excluded from the analysis. In the 10 studies (11 comparing) on depressive symptoms, tests of heterogeneity demonstrated no differences in effects across treatments (*τ*^2^=0.01; *χ*^2^_10_=11.09; *I*^2^=10%; *P*=.35).

### Results of Sensitivity Analysis

Subgroup analysis was conducted to verify an association between the studies’ quality and intervention effects. In the 4 studies (5 comparisons) judged to have a low risk of bias, the estimated pooled effect size (Hedge *g*) was −0.56 (95% CI −0.74 to −0.38, *P*<.001; *τ*^2^<0.001, *χ*^2^_4_=2.13, *P*=.71; *I*^2^=0%) and −0.42 (95% CI −0.69 to −0.16, *P*=.002; *τ*^2^=0.15, *χ*^2^_12_=42.01, *P*<.001; *I*^2^=71%) for the other 11 studies. Thus, our results suggest that study quality did not significantly affect intervention effects.

### Publication Bias

**Figure 3:**
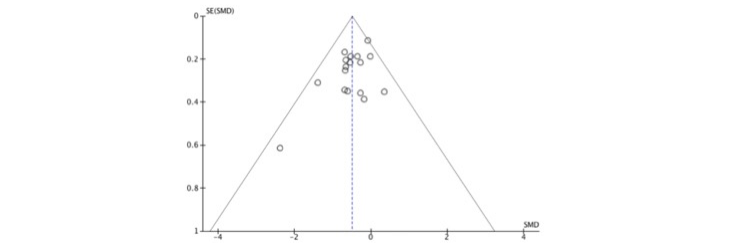
Funnel plot. SMD: standardized mean difference.

Figure 3 displays a funnel plot. Effect sizes were not evenly distributed around the averaged effect. The lower-right section of the funnel plot is devoid of studies, which suggests that there was bias in the pooled effect estimate owing to unpublished studies.

## Discussion

### Principal Findings

The objective of this systematic review was to investigate the effectiveness of VCBT as an intervention strategy as compared to control conditions such as AC, AT, TAU, and WLC using meta-analysis. Taken together, the results of this meta-analysis suggest that the pooled effect size of the primary outcomes of each disorder is medium, indicating that VCBT is especially effective for depressive symptoms. The novelty of this meta-analytic review lies in integrating the effectiveness of VCBT for psychiatric and somatic disorders, extending current knowledge into the field of remote psychotherapy [28,29,59]. However, most of the RCTs included in this review targeted depression. Therefore, further RCTs should be performed to accurately estimate the effectiveness of VCBT for generalized anxiety disorder, obsessive-compulsive disorder, chronic pain, and hypochondriasis. The quality of the study in the 16 RCTs (17 comparisons) was evaluated using RoB [43]: only 4 studies (5 comparisons) showed a low risk of bias, 10 showed some concern, and 2 showed a high risk of bias. Meta-analysis with the 4 studies (low risk of bias) revealed a moderate effect size and no heterogeneity for the effectiveness of VCBT. This result was also similar to those of a meta-analysis of 11 studies in which other judgments were made for all 16 RCTs (17 comparisons) and other bias risks. Therefore, the findings of this systematic and meta-analysis suggest evidence that VCBT is also effective for diseases for which face-to-face and guided ICBT have been demonstrated [8,16,17].

### Strengths of the Review

This meta-analysis has several strengths. The results of this systematic review and meta-analysis for psychiatric disorders extend support in favor of VCBT as an effective mode of intervention. Prior systematic reviews of CBT utilizing videoconferencing systems did not include meta-analysis [28,29]. Furthermore, this study has integrated the results of RCTs that directly compared the effectiveness of VCBT for typical psychiatric disorders with minimal intervention. Our results provide useful information for clinicians and policy makers to take the practicality of VCBT into account, especially in response to the COVID-19 pandemic [60].

### Limitations

This study also has some limitations. First, the analyzed studies were highly heterogeneous. Second, this study adopted a broad definition of CBT. CBT is a broad concept that includes treatment methods such as cognitive therapy, behavioral therapy, acceptance and commitment therapy, behavioral activation, problem-solving techniques, and prolonged exposure therapy. Therefore, the effect size should ideally be estimated for each therapeutic technique in the future, as was performed in a previous review of cognitive therapy for depression [61]. However, the effectiveness of each cognitive behavioral technique could not be calculated in this study, owing to the small number of pre-existing studies to analyze intervention categories and subcategories. Furthermore, owing to the inconsistent control criteria in this review, it is not possible to assess the exact effectiveness of VCBT. The gold-standard design for estimating the effect of treatment is RCT with psychological or the pill placebo group [62]. These control conditions can be standardized to control the impact of patient expectations on outcomes. Therefore, to estimate the effectiveness of VCBT accurately, it is necessary to perform a meta-analysis at the stage when RCT with psychological or pill placebo group is sufficiently accumulated. Further, this review only included studies that used adult participants owing to the low number of RCTs for children and adolescents. In future, RCTs to evaluate the effectiveness of VCBT should also be conducted on samples of children and adolescents. Since we only included articles written in English, we need to examine studies reported in more diverse languages in the future. Finally, long-term effectiveness of VCBT was not analyzed. Since this study succeeded in demonstrating the short-term effectiveness of VCBT for adult psychiatric disorders and somatic symptom disorders, the results of long-term effectiveness in the future RCTs should also be integrated.

### Comparison With Prior Work

Our results, indicating that VCBT is effective for somatic disorders, extend support to those of Liu et al [9], who compared VCBT and face-to-face CBT. Additionally, the overall effect size estimated in this meta-analysis is very similar to previous results on face-to-face CBT and computerized CBT for treatment of depression and anxiety, compared to primary care TAU [61,63]. Furthermore, the effect size (Hedge *g*=0.46, medium effect size) of VCBT for depressive symptoms is consistent with previous results (Hedge *g*=0.71, large effect size) of a bias-adjusted meta-analysis [64]. Therefore, our findings demonstrate the effectiveness of VCBT as a treatment option for adults with psychiatric disorders.

This review did not include exposure techniques except for an RCT by Vogle et al [21]. Real-time interventions that utilize videoconferencing systems have the advantage of exposing the home environment of the patient [65]. At the same time, it is difficult to match up to the interpersonal experience of working with a therapist to resolve the issues, especially in the treatment for disorders that require exposure therapy such as obsessive-compulsive disorder and panic disorder. In case of obsessive-compulsive disorder, patients are afraid of things such as hospital floors, rags, and toilet paper. In case of panic disorder, the task of working with the therapist to climb stairs and exercise may be difficult to perform. Therefore, it would be premature to determine the effectiveness of VCBT for patients with anxiety disorders from the effect sizes shown in this review. The results of clinical studies that were not RCTs show that VCBT is sufficient to improve symptoms of obsessive-compulsive disorder and panic disorder [18,21,65], where exposure is an important therapeutic component [66,67]. In the future, tightly controlled RCTs are expected to be implemented, and this review should be updated with regard to the calculation of integrated estimated effect sizes for those disorders.

The number of studies included in the analysis corresponding to each condition was small, except for depressive symptoms. However, the total number of studies and participants made it possible to detect significant differences between VCBT and control conditions. However, the high degree of heterogeneity indicates the need for careful interpretation of our results. While the risk of bias detected in the quality assessment appeared to have an overall minor impact, there may be publication bias, as no negative results were reported. Namely, the funnel plot in this study suggests that there was bias in the pooled effect estimate owing to unpublished studies. Positive findings are thrice more likely to be published than negative findings [68]; hence, careful interpretation of our results is required.

### Conclusions

This study attempted to provide evidence in favor of the effectiveness of VCBT as a feasible alternative approach to service patients with poor access to face-to-face CBT. VCBT, has the advantage of facilitating real-time communication between patients and therapists. This has important implications for clinicians and policy makers because it is a well-accepted approach that has demonstrated a high degree of satisfaction [19]. Although more studies are needed to draw firm conclusions, findings such as those from our meta-analysis show that VCBT is a promising treatment for future use [69].

## Appendix

Multimedia Appendix 1 PRISMA 2020 checklist.

Multimedia Appendix 2 The complete search strategy, details of the included studies, and list of excluded studies.

## Abbreviations

AC: active control
AT: attention training
CBT: cognitive behavioral therapy
ICBT: internet-based cognitive behavioral therapy
PRISMA: Preferred Reporting Items for Systematic Reviews and Meta-Analyses
RCT: randomized controlled trial
SMD: standardized mean difference
TAU: treatment as usual
VCBT: videoconference-delivered cognitive behavioral therapy
WLC: wait-list control
