# Supplementary material for: Effectiveness of Videoconference-Delivered Cognitive Behavioral Therapy for Adults With Psychiatric Disorders: Systematic and Meta-Analytic Review
Source: J Med Internet Res. 2021 Dec 13;23(12):e31293. doi: 10.2196/31293 (PMC8713091; doi:10.2196/31293)
Supplement: Multimedia Appendix 2 [file jmir_v23i12e31293_app2.pdf]

Supplementary file

Supplementary file 1.

The Complete Search Strategy

Abstract, Search methods
MECIR R6 – Include date of last search, indicate databases and sources searched

We searched MEDLINE (via PubMed), Web of Science, Science Direct, PsychINFO, CINAHL, LILACS, CiELO, and [x] trials registers (ClinicalTrials.gov, Cochrane Central Resister of Controlled Trials, www.ensaiosclinicos.gov.br) to [25-December, 2020], together with reference checking, citation searching and contact with study authors to identify additional studies.

Search methods for identification of studies
MECIR R34 Search sources. List all sources searched<br>MECIR R35 Latest searches. Provide the dates of the last search and issue/version number for each database where relevant<br>MECIR R38 Search strategies for bibliographic datebases

Electronic searches

We used the criteria and standard methods of Cochrane. We conducted a comprehensive search including: the Cochrane Central Register of Controlled Trials (CENTRAL) in the Cochrane Library; and MEDLINE via PubMed to 25 December, 2020; using the following search terms: "depression," "panic disorder," "social phobia," "social anxiety disorder," "generalized anxiety disorder," "obsessive-compulsive disorder," "post-traumatic stress disorder," "specific phobia," "hypochondriasis," "bulimia," "tinnitus," "erectile dysfunction," "chronic pain," or "fatigue." To determine the intervention approach, these search terms were combined with "videoconference," "video conference," "videoconferencing," "tele," "teleconference," "tele conference," or "teleconferencing," plus database-specific limiters for RCTs (see Appendix 1 for the full search strategies for each database). We apply language restrictions in English. We searched clinical trials registries for ongoing or recently completed trials.

Searching other resources
MECIR R37 searchers for different types of evidence e.g. adverse effects<br>MECIR R39 Search strategies for other sources

Adverse effects

We did not perform a separate search for adverse effects of interventions used for the treatment of VCBT. We considered adverse effects described in included studies only.

Searching within other reviews

The Information Specialist searched MEDLINE to retrieve existing systematic reviews relevant to this systematic review, so that we could scan their reference lists for additional trials.

Searching reference lists

Supplementary file

We searched the reference lists of papers reporting studies selected for inclusion in this review in order to identify additional relevant trials.

Searching by contacting individuals

Where necessary, we contacted authors of key papers and abstracts to request further information about their trials.

Conference proceedings

We did not search for conference abstracts.

Results of the search

The searches of the seven databases (see Electronic searches) retrieved 4,277 records. Our searches of the trials registers identified 1,217 further studies. Our screening of the reference lists of the included publications did/did not reveal 0 additional RCTs. We therefore had a total of 5,493 records.

Once duplicates had been removed, we had a total of 3,684 records. We excluded 3,622 records based on titles and abstracts. We obtained the full text of the remaining 62 records. We excluded 46 studies (see Characteristics of excluded studies). We added 16 records to Characteristics of studies awaiting classification.

For a further description of our screening process, see the study flow diagram (Figure 1).

Discussion

MECIR R100 Limitations

Potential biases in the review process

We attempted to conduct a comprehensive search for studies, but the fact that two studies are awaiting classification and have not yet been incorporated may be a source of potential bias.

Authors' conclusions

MECIR R101 Conclusions: implications for practice

Authors conclusions: Implication for practice

There are two studies that we have identified as potentially relevant but have yet to classify; these may alter the conclusions of the review once assessed (see Studies awaiting classification).

Supplementary file

Supplementary file 2**.**

Table S1. Detail of the included studies

**Studies**	**Detail**
**Ahmad (2020)**	**Population:** Eligibility criteria were a minimal age of 18, fluency in English, self-reported reliability to complete the study, with ability to use computers and smartphones, and with internet literacy. The mean age of the participants was 24.8 (SD = 6.5), and 28 males (24.8%) and 85 females (75.2%) participated in this RCT. The mean age of 39 participants in the intervention group was 24.9 years (SD = 6.4) with 10 males (26.0%) and 29 females (74.0%), and 39 in the WLC group were 25.4 (SD = 7.3) with eight males and 31 females (21.0%) and 31 females (80.0). Eligibility criteria did not include to diagnose tool by DSM or ICD, cutoff scores to assess depression symptom at screening.<br>**Intervention**: The Mindfulness Virtual Community Program was weekly conducted in the RCT for eight-week. The intervention also included 20-min live videoconferences on module topics guided by a mental health professional.<br>**Comparison**: Wilt-list control (WLC)<br>**Outcomes**: The Patient Health Questionnaire (PHQ) is a self-administered version of the PRIME-MD diagnostic instrument for common mental disorders. The PHQ-9 is the depression module, which scores each of the 9 DSM-IV criteria as “0” (not at all) to “3” (nearly every day).<br>**Setting**: This RCT was conducted at York University, Toronto. The recruitment of eligible undergraduate students occurred during December 7, 2016 and January 10, 2017. These students started the parallel-arm RCT on January 16, 2017, with a baseline survey followed by exposure to 2 interventions, a 4-week (middle-intervention) online survey, and an 8-week (post-intervention) online survey. The 8-week-long interventions started on January 22, 2017 and ended on March 16, 2017.<br>**Define of community and clinical sample:** Participants in this RCT were students at York University in Toronto. Therefore, the participants belonged to the same community.<br>**Funding**: The Canadian Institutes for Health Research (CIHR), eHealth Innovations Partnership Program Grant; grant number EH1-143553.
**Alschuler (2021)**	**Population**: In this RCT, participants were 27 patients with pain in multiple sclerosis, predominantly male (66.7%), non-Hispanic White (74.1%), married (55.6%). Most participants reported their disease course was relapsing remitting (77.8%), mean time since MS diagnosis was just over two years. Mean age was 40.1 (SD = 11.2) and 39.6 (SD = 12.3), in the intervention and control groups, respectively.<br>**Intervention**: The psychological pain management intervention was designed as a 120 min group videoconference session, focused on developing an adaptive set of pain coping strategies based upon cognitive behavioral theories of pain. The module included education on pain and theoretical models of chronic pain, pain coping, relaxation training, a brief module on pacing, cognitive restructuring, and cognitive delusion.<br>**Comparison**: TAU.<br>**Outcomes**: The Pain Catastrophizing Scale (PCS) was used to assess pain catastrophizing in this RCT. The PCS is a well-validated 13-item self- report measure that produces a total score (higher score = more catastrophizing), as well as subscale scores across rumination, magnification, and helplessness.<br>**Setting**: The RCT was conducted from the UW Medicine Multiple Sclerosis Center (University of Washington, Seattle, WA). Pre-treatment (baseline) data were collected 1 to 2 weeks prior to the intervention. Accept-ability, impact, and outcome data were collected at post-treatment (1–2 weeks postcompletion of the study intervention) and 3 months (2 weeks) following the treatment (follow-up).<br>**Define of community and clinical sample**: Clinical sample.<br>**Funding**: The National Multiple Sclerosis Society (PP-1609-25787).
**Bogosian (2015)**	**Population**: Participants were recruited through adverts on the Parkin- son’s UK and the Michael J. Of the 60 participants in this RCT, 30 were assigned to the intervention group (mean age 59.5, SD = 11.12, 13 female) and 30 to the WLC group (mean age 62.2, SD = 8.96, 17 female).<br>**Intervention**: The mindfulness-based intervention via videoconference was delivered in 8 sessions over 8 weeks. The sessions were carried out by the facilitator.

Supplementary file

	**Comparison**: WLC.<br>**Outcomes**: The Hamilton Depression Rating Scale (also known as the Ham-D) is the most widely used clinician-administered depression assessment scale. The original version contains 17 items (HDRS17) pertaining to symptoms of depression experienced over the past week. A limitation of the HDRS is that atypical symptoms of depression (e.g., hypersomnia, hyperphagia) are not assessed.<br>**Setting**: Fox Foundation websites and emails sent to the Parkinson's UK Research Network. Recruitment took place between February and March 2016. Participants were randomly assigned to either an 8-week the intervention or a WLC. Endpoints were 4 time: baseline, mid-intervention (four-week), post-intervention (eight-week), and follow-up (12-week).<br>**Define of community and clinical sample:** This study targeted at people with Parkinson's disease, hence, the participant of the RCT was clinical sample.<br>**Funding**: This RCT was conducted at City, University of London and was supported by Parkinson's UK under Grant K-1409.
**Choi (2014)**	**Population**: Eligibility criteria were PHQ-9>10, 50 years and older, and a 24-item Hamilton Rating Scale for Depression (HAMD) of 15 points or higher. The mean age of the participants was 65.21 (SD = 9.22), 27 males (22.3%) and 94 females (SD = 77.7). In family income <= 15,000 was 77 (63.6%), 15,001-25,000 was 25 (20.7%), and 25001-50,000 was 7 (5.8%) of the participants. Eighty-one patients (67%) were classified as Major Depressive Disorder (MDD) in the diagnostic classification of SCID.<br>**Intervention**: Six 60-minute sessions of the problem-solving therapy-primary care version were conducted by using videoconference, weekly.<br>**Comparison**: Telephone support calls, weekly.<br>**Outcomes**: The Hamilton Depression Rating Scale (also known as the Ham-D) is the most widely used clinician-administered depression assessment scale. The original version contains 17 items (HDRS17) pertaining to symptoms of depression experienced over the past week. A limitation of the HDRS is that atypical symptoms of depression (e.g., hypersomnia, hyperphagia) are not assessed.<br>**Setting:** In this RCT, the baseline and 12- and 24-week follow-up HAMD scores were used.<br>**Define of community and clinical sample:** Participants were older adult who were referred to the project by case managers at a large Meals on Wheels (MOW) and other aging-network agencies serving low-income individuals in central Texas, USA. Therefore, this study used community-based samples.<br>**Funding**: This RCT was funded by the National Institute of Mental Health (R34 MH083872).
**Choi (2020a)**	**Population**: People with mild to Middle Eastern depressive symptoms with PHQ-9 <10, over 50 years old in Texas, and over 60 years old in New Hampshire met the selection criteria. The average age of the participants was 74 years (SD = 9.0), 62% were female. Among 277 participants, 193 (69.7%) were women, 83 (30.0%) were Black, and 81 (29.2%) were Hispanic (Table 1). The mean (SD) age was 67.5 (8.9) years, and 255 participants (92.1%) had an annual income of $35 000 or less.<br>**Intervention**: Tele-behavioral activation (Tele-BA) of 5 weekly, 1-hour video conferenced session.<br>**Comparison**: Teel-friendly visits.<br>**Outcomes**: The Patient Health Questionnaire (PHQ) is a self-administered version of the PRIME-MD diagnostic instrument for common mental disorders. The PHQ-9 is the depression module, which scores each of the 9 DSM-IV criteria as "0" (not at all) to "3" (nearly every day).<br>**Setting**: The study was conducted on June 16, 2017 and completed on September 1, 2020. Of 89 participants, 81 and 80 completed intervention sessions and 6-week and 12-week follow-up assessments, respectively.<br>**Define of community and clinical sample**: Study participants were referred to the investigators by case managers of a home-delivered meals (HDM) program in a large city in Central Texas and a HDM program of the New Hampshire consortium of five aging service agencies that largely serve rural areas. Therefore, the participants of this RCT belong to the same community.<br>**Funding:** AARP Foundation (ISO-2017-07-001; PI: M. Bruce).
**Choi (2020b)**	**Population**: Among 277 participants, 193 (69.7%) were women. The mean (SD) age was 67.5 (8.9) years. Our study cohort closely represented the overall population of individuals in the study area who receive home-delivered meals. 142 participants (51.3%) were using 1 or more antidepressant medications. In the diagnostic classification of SCID-5, 42 people were applicable to MDD and 172 corresponded to persistent depression (dysthymia).

Supplementary file

	**Intervention**: A 5-step Tele-BA and 7-step Tele-Problem-solving therapy (Tele-PST) by two lay counselors with Before working with participants, lay counselors, one with a bachelor's degree in social work, the other with a bachelor's degree in communication, received a 50-hour didactic training in depression, BA, and care coordination and practiced tele-BA sessions with 3 older adults who were homebound and depressed under the supervision of a licensed clinical social worker (L.S.). The licensed clinical social worker also provided clinical supervision and fidelity monitoring of 20% of all sessions during the intervention phase. Tele-PST was performed in a similar manner to Choi et al. (2014).<br>**Comparison**: Attention control.<br>**Outcome**: The Hamilton Depression Rating Scale (also known as the Ham-D) is the most widely used clinician-administered depression assessment scale. The original version contains 17 items (HDRS17) pertaining to symptoms of depression experienced over the past week. A limitation of the HDRS is that atypical symptoms of depression (e.g., hypersomnia, hyperphagia) are not assessed.<br>**Setting:** From February 15, 2015, to April 15, 2019, home-delivered meals and aging services case managers referred 505 individuals aged 50 years or older who were homebound and who were residing in Central Texas to the study team. This trial's term was February 15, 2016 to April 15, 2019. Assessments were performed at baseline and at 12, 24, and 36 weeks.<br>**Define of community and clinical sample**: Users of home-delivery meal services living in Central Texas, USA, are participating in this RCT. Therefore, this RCT is a clinical trial with community samples.<br>**Funding**: This study was supported by grant No. 1R01MD009675 from the National Institute on Minority Health and Health Disparities.
**Demiris (2019)**	**Population**: A total number of 514 caregivers who participated in the study (AC, n = 172; PSI, n = 171). Caregivers ranged in age from 19 to 100 (mean age = 60.3 y), were predominantly female (75%), and mostly adult children (55%) or spouses/partners (27%) of hospice patients.<br>**Intervention**: Problem-solving intervention (PSI) via videoconferencing involved five steps: adopting a positive attitude, defining the problem, creating alternatives, predicting consequences, and trying a solution. Two interventionists (one psychologist and one social worker) employed by our team received 25 hours of PST training.<br>**Comparison**: Friendly calls (attention control, AC).<br>**Outcome**: The Generalized Anxiety Disorder 7-Item (GAD-7) is a brief, valid, and efficient tool for screening for anxiety and assessing its severity in clinical practice and research. The GAD-7 was tested and demonstrated high levels of reliability and validity. There were no differences in caregivers in the VC condition compared with the AC condition.<br>**Setting**: Two large hospice agencies in the Pacific Northwest participated in the project. This study was completed in October 2011 and March 2016. One last follow-up assessment over the phone took place approximately 40 days after the exit interview.<br>**Define of community and clinical sample**: Participants were family/informal caregiver of a hospice patient of two large hospice agencies in the Pacific Northwest. Therefore, the target population of this RCT was a community sample.<br>**Funding**: The National Institute of Nursing Research of the National Institutes of Health (NIH) under Award Number R01NR012213.
**Elliontt (2008)**	**Population**: Consenting participants included seven men (Mean age 59.71 years, SD 16.64) and 54 women (Mean age 47.13, SD 14.19) in caregiver roles for persons with spinal cord injuries. The sample comprised 42 Caucasian and 19 African-American individuals. Most caregivers were spouses (N=24) and parents (N=19) of the care recipient. Other caregivers were daughters (N=45), grandparents (N=44) and siblings (N=43) of the care recipient. Their care recipients also consented to participate (40 men, mean age 38, SD 14.83 years; 21 women, Mean age 46.29, SD 9.09 years).<br>**Intervention**: PSI via videoconferencing was delivered by two coordinators with doctorates in clinical psychology from accredited programs. The first session with the caregiver required approximately 2–3 hours in a face-to-face session to conduct the baseline assessment and provide the orientation. Manuals containing information about problem-solving principles were also provided in the first session. Participants receiving problem-solving training were first oriented to the basic steps of the problem-solving process.

Supplementary file

	**Comparison**: Education only. Participants assigned to the education-only control group received telephone contacts from a research staff member who discussed educational concerns with the participation. Educational materials were provided at scheduled intervals and as needed, based on the unique interests and issues of each participant. The two coordinators provided educational materials to and interacted with participants assigned to the control group.<br>**Outcome**: The Inventory to Diagnose Depression (IDD). IDD is a 22-item self-report scale designed to diagnose major depressive disorder (MDD) according to Diagnostic and Statistical Manual of Mental Disorders (DSM-III; American Psychiatric Association, 1980) criteria.<br>**Setting**: Participants completed the IDD at pre-treatment, 6 months, and 12 months.<br>**Define of community and clinical sample**: Prospective participants were recruited from the inpatient rehabilitation program and from the community. Therefore, the target population of this RCT was a community sample.<br>**Funding**: The National Institute on Disability and Rehabilitation Research, Office of Special Education and Rehabilitative Services, US Department of Education (H133B90016) and by Grant No. R49/CCR403641 from the US Department of Health and Human Services, Centers for Disease Control and Prevention, National Center for Injury Prevention and Control to the University of Alabama at Birmingham, Injury Control Research Center.
**Ferguson (2016)**	**Population**: 47 survivors of female breast cancer, mean age 54.6 (SD = 12.12). Exclusion criteria included: 1) previous treatment with central nervous system radiation or intrathecal therapy; 2) surgery involving the central nervous system; 3) neurobehavioral risk factors including brain injury; 4) a history of neurological disorder, substance abuse, or learning disability; 5) meeting criteria for an active Axis I psychiatric disorder (Diagnostic and Statistical Manual of Mental Disorders, 4th Edition, Text Revision [DSM-IV-TR]); and 6) severe hearing impairment.<br>**Intervention:** Memory and Attention Adaptation Training (MAAT) as a cognitive behavioral therapy (CBT) via videoconferencing is consisting of 8 weekly visits of 30 to 45 minutes each<br>**Comparison**: Supportive therapy (ST) controls for nonspecific psychotherapeutic factors of the clinician-participant alliance, empathy, support, and warmth14 while not providing the active behavior change training contained within MAAT ("behavioral placebo").<br>**Outcomes**: The Depression Anxiety Stress Scales-21 (DASS-21) is the short version of a self-report measure that was originally developed to provide maximum differentiation between depressive and anxious symptoms.<br>**Setting**: Assessment was conducted at baseline, after treatment, and at 2 months of follow-up.<br>**Define of community and clinical sample**: Participants had a diagnosis of Stage I, II, or IIIA breast cancer (TNM staging system of the of Union for International Cancer Control). Therefore, the target population of this RCT was a clinical sample.<br>**Funding**: The National Institutes of Health Office of Research on Women's Health administrated by the National Cancer Institute (R21CA143619-01A1).
**El-Jawahri (2019)**	**Population**: Participants were family caregivers of patients undergoing hematopoietic stem cell transplantation (HCT). Enrolled caregivers were mostly white (92.4% [85 of 92]), and the majority were female (69.6% [64 of 92]) and married to the patient (81.5% [75 of 92]); the median age was 61 years (range, 22-93 years). Most caregivers (64.1%) were caring for an allogeneic HCT recipient.<br>**Intervention**: A study team consisting of oncologists and psychologists collaboratively developed the structured, conducted manualized the intervention. The intervention was a 6-session caregiver-directed coping skills intervention that integrates HCT-related education with cognitive behavioral strategies to enhance caregiver knowledge and skills across the transplant trajectory. Before meeting with any participants, all study interventionists completed a half-day of in-person training and attended weekly telephone-based group clinical supervision throughout the course of the study with the lead licensed clinical psychologist.<br>**Comparison**: Treatment as usual (TAU).<br>**Outcomes**: To assess mood and anxiety symptoms, caregivers completed the Hospital Anxiety and Depression Scale (HADS). The 14-item HADS consists of 2 sub-scales assessing anxiety and depression symptoms in the past week. Subscale scores on the HADS range from 0 (no distress) to 21 (maximum distress).<br>**Setting**: The study was conducted at the Massachusetts General Hospital Cancer Center, at Boston, Massachusetts, USA. We approached 138 caregivers for study participation between December 2017 and April 2019, and 72.5% (100 of 138) enrolled. Overall, 83 and 87 caregivers completed the day 30 and day 60 post-HCT

	assessments with missing data rates of 9.8% and 5.4%, respectively.<br>**Define of community and clinical sample:** Clinical sampling.<br>**Funding**: A National Institutes of Health K12 Career Development Award.
**Fox (2020)**	**Population**: Participants were men with stage Ⅲ or IV advanced prostate cancer (APC) had an mean age of 71.31 years old (SD = 8.9).<br>**Intervention**: Cognitive behavioral stress management (CBSM) treatment was delivered through WebEx for approximately 60 minutes each week. The intervention was adapted to information and situational examples relevant to APC.<br>**Comparison**: a 10-week, AC.<br>**Outcomes**: Patient Reported Outcome Measurement Information System. Patient reported psychosocial functioning was assessed weekly using the PROMIS Anxiety, Depression, Fatigue, Pain Interference. Higher scores represent more symptoms of anxiety, depression, fatigue, and pain interference, and better physical function.<br>**Setting**: Participants were recruited from Northwestern Medicine- affiliated hospitals, the Jesse Brown VA Medical Center, and Rush University Medical Center. Participants enrolled and provided written informed consent from January 2013 through November 2016. An assessment was performed 1 and 10 weeks after the intervention.<br>**Define of community and clinical sample:** This study has submitted the results of RCTs using clinical samples performed at specific institutions.<br>**Funding**: This study was supported by an NCI grant (R01CA157809).
**EI-Morr (2020)**	**Population**: A total of 160 undergraduate student were 32 males (20.1%) and 125 females (78.6%) who participated with 2 students declaring gender fluid and nonbinary genders. Most participants were born outside of Canada (87/159, 54.7%) and reported English as their first language (93/159, 58.5%), and 20.1% (32/159) of the sample self-identified as White. Most participants did not have access to private mental health insurance (102/159, 64.1%).<br>**Intervention**: An 8-week web-based mindfulness and CBT program. The intervention comprised 3 components: (1) 12 student-specific mental health modules conveyed by online video; (2) 3 anonymous discussion boards dedicated to depression, anxiety, and stress; and (3) an anonymous 20-minute group-based live videoconference led by a moderator (a counselor with a master's degree in psychology and training in mindfulness) during which students could raise and discuss topics covered in the modules.<br>**Comparison**: WLC.<br>**Outcomes**: The Patient Health Questionnaire (PHQ) is a self-administered version of the PRIME-MD diagnostic instrument for common mental disorders. The PHQ-9 is the depression module, which scores each of the 9 DSM-IV criteria as "0" (not at all) to "3" (nearly every day).<br>**Setting**: A sample of 480 students (240 students per group) was recruited over 3 semesters (Fall 2017, Winter 2018, and Fall 2018). However, the 3 samples could not be combined due to substantial differences in the campus environment. Notably, in the Fall 2017 semester the platform functionalities presented connection challenges to students and the platform did not capture the user analytics correctly via the built-in tools, a problem which was corrected for subsequent semesters. In addition, during the Winter 2018 semester the university was disrupted by an employee strike of 3 months' duration. Prior to the Fall 2018 semester (the semester during which this study was undertaken), the strike was resolved, and the university resumed routine functioning. The results of this RCT is based on the sample recruited in Fall 2018.<br>**Define of community and clinical sample**: Sampling was conducted in undergraduate students at a large Canadian university, community sample.<br>**Funding**: The Canadian Institutes for Health Research (CIHR) eHealth Innovations Partnership Program Grant (eHIPP; Grant No. EH1-143553)
**Morriss (2019)**	**Population**: Participants were the 524 patients with health anxiety referred to the study, 470 were eligible and 156 (33%) participants were recruited. Most participants (80%) were referred from primary care by their GP. Two-thirds were females and half were under 35 years of age, although there was a wide age range. The mean (SD) scores at baseline on the GAD-7 and the PHQ-9 were above clinical cut-offs (> 8 and>10, respectively) for both groups.<br>**Intervention**: A team of four experienced CBT therapists remotely de- livered CBT for health anxiety using a treatment manual developed from the Cognitive Behavioral

Supplementary file

	Therapy for Health Anxiety in Medical Patients study. Between six and 12 sessions of CBT were offered, with up to three booster sessions if required. This included an initial 'setup' session, during which the methods used to adapt CBT to remote delivery were discussed and any concerns about this method were addressed.<br>Comparison: TAU.<br>**Outcomes**: The 14-item Short Health Anxiety Inventory (SHAI), a measure of health anxiety, was used. Each item is comprised of four statements from which the respondent chooses the statement that best captures their experience over the past week. Items are scored on a scale from 0 (no symptoms) to 3 (severe symptoms) and the sum of the items produced a total score of health anxiety (0–42), with higher scores reflecting higher levels of health anxiety.<br>**Setting**: This single-blind, patient-level, parallel group, multicenter randomized controlled trial (RCT) was conducted in primary and secondary care centres across the East Midlands and at two other English sites. Participants were referred to the study and were assessed for eligibility between 19 November 2014 and 31 December 2016. Data were collected at baseline, 3, 6, 9 and 12 months.<br>**Define of community and clinical sample**: This RCT was conducted in primary and secondary care centers across the East Midlands and at two other English sites. Therefore, clinical samples were targeted.<br>**Funding**: The National Institute for Health Research (NIHR) Collaboration for Leadership in Applied Health Research and Care East Midlands, with matched funding from NHS Trust sites and the University of Nottingham.
**Somers (2018)**	**Population**: Participants included 32 individuals with HCT pain who had undergone autologous (87%, 28/32) or allogeneic (13%, 4/32) stem cell transplant and reported having post-transplant pain. Participants were 50% female (16/32), 72% Caucasian (23/32), and were aged between 43 and 76 years (mean 61). Majority of the participants were married (84%, 27/32) and 53% (17/32) had a college degree or higher.<br>**Intervention**: The Mobile Pain Coping Skills Training (MPCST)included 6, 50-min sessions delivered over a period of 6-10 weeks, to help patients understand that pain is a complex experience influenced by thoughts, feelings, and behaviors. Patients are taught several skills (eg, relaxation training, cognitive-restructuring, activity pacing, pleasant activity planning, imagery, problem solving, and goal setting) to enhance their ability to cope with their pain by changing their thoughts, feelings, and behaviors.<br>**Comparison:** TAU.<br>**Outcomes**: The Primary outcome of pain was pain severity was assessed with the 4-item brief pain inventory. Patients rated their pain from 0=no pain to 10=worst pain imaginable in response to average pain, worst pain, least pain, and pain right now over the past 7 days. An average of the 4 items was used to create a single pain severity score.<br>**Setting:** All participants were recruited from the adult bone marrow transplant clinic (ABMT) at a major academic medical center. Assessment was performed at baseline and post-treatment.<br>**Define of community and clinical sample:** Clinical sample at academic medical center in USA.<br>**Funding**: Funding for this project (Grant Number: 1R21CA173307-01A1) was provided by National Institute of Health (NIH).
**Vogel (2014)**	**Population**: Thirty patients with OCD (mean age 28.8, SD = 9.2 in the intervention group; 40.7, SD = 11.1 in the WLC group) participated in this RCT. The proportion of female patients was 6 in the intervention group and 7 in the WLC group. The proportion of participants with depression was two in the intervention group and four in the WLC group. The use of selective serotonin reuptake inhibitor was permitted during this RCT.<br>**Intervention**: Exposure Response Prevention via video conferencing was conducted for obsessive-compulsive disorder. The videoconference sessions allowed some direct observation of in vivo exposures to "contaminated" objects, or imaginary expo- sures to feared situations, and the concomitant prevention of ritualistic behaviors was monitored.<br>**Comparison**: WLC.<br>**Outcomes**: Obsessive-compulsion and obsessive-compulsion are composed of 10 items, and the severity of each item is evaluated from 0 to 4. Therefore, the range of Y-BOCS total points is 0 to 40 points.

Supplementary file

	**Setting**: Assessments was performed before (baseline), after (post-treatment), and 3 months after this RCT.<br>**Define of community and clinical sample**: Participants were sampled from outpatient treatment at specialized clinics in Oslo, Kristiansand, and Trondheim, Norway. Therefore, this RCT was performed by clinical sampling.<br>**Funding**: The study was supported by a grant from the Norwegian Extra Foundation for Health and Rehabilitation (grant number 2009/3/0075).
**Vranceanu (2019)**	**Population**: The participants were patients with an orthopedic injury in the prior 1–2 months, 18 years or older, English fluency and literacy, and over median level of pain. The average age of participants was 51 years (SD = 16) and 50 years (SD = 21), in the intervention group and the control group. The proportion of women was 57.4% (n = 31/54).<br>**Intervention**: The intervention was a four-session, live video, manualized mind-body program informed by the fear avoidance model: Relaxation response skills; cognitive behavioral skills; acceptance and commitment therapy skills.<br>**Comparison**: TAU.<br>**Outcomes**: The Short Musculoskeletal Function Assessment Ques- tionnaire (SMFA) is a validated 46-item questionnaire that measures physical functioning/musculoskeletal disability. It is developed from the 101-item parent questionnaire which has been extensively validated and tested for reliability and responsiveness. The score is calculated by summing up the individual items which cover assessment of function (34 questions) and percep- tion of how bothersome symptoms are (12 questions). All questions are answered on a 4-point Likert scale with high scores depicting higher disability. Raw scores are summed and transformed so that the final score ranges from 0 to 100.<br>**Setting:** Recruitment occurred between January 2016 and May 2018. In the RCT, the researchers collected data at baseline, 4–5weeks after baseline, and 4 months post-baseline (3 months after post-test).<br>**Define of community and clinical sample**: Clinical sample.<br>**Funding**: This study was supported by an Orthopedic Research and Education Foundation (OREF) grant

Supplementary file

Supplementary file

Supplementary file 3.

Revised Cochrane risk-of-bias tool for randomized trials (RoB 2)
TEMPLATE FOR COMPLETION

Edited by Julian PT Higgins, Jelena Savović, Matthew J Page, Jonathan AC Sterne
on behalf of the RoB2 Development Group

Version of 22 August 2019

The development of the RoB 2 tool was supported by the MRC Network of Hubs for Trials Methodology Research (MR/L004933/2- N61), with the support of the host MRC ConDuCT-II Hub (Collaboration and innovation for Difficult and Complex randomised controlled Trials In Invasive procedures - MR/K025643/1), by MRC research grant MR/M025209/1, and by a grant from The Cochrane Collaboration.

This work is licensed under a Creative Commons Attribution-NonCommercial-NoDerivatives 4.0 International License.

Study details

Supplementary file

**Reference**	Ahmad F, El Morr C, Ritvo P, Othman N, Moineddin R; MVC Team. An Eight-Week, Web-Based Mindfulness Virtual Community Intervention for Students' Mental Health: Randomized Controlled Trial. *JMIR Ment Health*. 2020;7(2):e15520. Published 2020 Feb 18. doi:10.2196/15520

Study design

X Individually-randomized parallel-group trial
☐ Cluster-randomized parallel-group trial
☐ Individually randomized cross-over (or other matched) trial

For the purposes of this assessment, the interventions being compared are defined as

Experimental:	Mindfulness with professionally guide via videoconferences	Comparator:	Wait-list control

**Specify which outcome is being assessed for risk of bias**	The Patient health questionnaire-9 items (PHQ-9)
**Specify the numerical result being assessed.** In case of multiple alternative analyses being presented, specify the numeric result (e.g. RR = 1.52 (95% CI 0.83 to 2.77) and/or a reference (e.g. to a table, figure or paragraph) that uniquely defines the result being assessed.	The score reductions for PHQ-9 were statistically significant in both unadjusted (T2 unadjusted score change −2.47; P=.01; T3 unadjusted score change −3.39; P<.001) and adjusted (T2 adjusted score change −3.00; P=.015; T3 adjusted score-change −4.03; P<.001) analysis.

Is the review team's aim for this result…?

X to assess the effect of *assignment to intervention* (the 'intention-to-treat' effect)
☐ to assess the effect of *adhering to intervention* (the 'per-protocol' effect)

If the aim is to assess the effect of *adhering to intervention*, select the deviations from intended intervention that should be addressed (at least one must be checked):

☐ occurrence of non-protocol interventions
☐ failures in implementing the intervention that could have affected the outcome
☐ non-adherence to their assigned intervention by trial participants

Which of the following sources were <u>obtained</u> to help inform the risk-of-bias assessment? (tick as many as apply)

X Journal article(s) with results of the trial

Supplementary file

- Trial protocol
- Statistical analysis plan (SAP)
- X Non-commercial trial registry record (e.g. ClinicalTrials.gov record)
- Company-owned trial registry record (e.g. GSK Clinical Study Register record)
- “Grey literature” (e.g. unpublished thesis)
- Conference abstract(s) about the trial
- Regulatory document (e.g. Clinical Study Report, Drug Approval Package)
- Research ethics application
- Grant database summary (e.g. NIH RePORTER or Research Councils UK Gateway to Research)
- Personal communication with trialist
- Personal communication with the sponsor

Supplementary file

Risk of bias assessment

Responses underlined in green are potential markers for low risk of bias, and responses in red are potential markers for a risk of bias. Where questions relate only to sign posts to other questions, no formatting is used.

Domain 1: Risk of bias arising from the randomization process

Signalling questions	Comments	Response options
**1.1 Was the allocation sequence random?**	Yes. Conducted in "computer-generator by an off-stie team member" and "using block randomisation."	Y / PY / PN / N / NI
**1.2 Was the allocation sequence concealed until participants were enrolled and assigned to interventions?**	Yes. Used opaque envelops.	Y / PY / PN / N / NI
**1.3 Did baseline differences between intervention groups suggest a problem with the randomization process?**		Y / PY / PN / N / NI
**Risk-of-bias judgement**	Low risk of bias.	Low / High / Some concerns
Optional: What is the predicted direction of bias arising from the randomization process?	NA.	NA / Favours experimental / Favours comparator / Towards null /Away from null / Unpredictable

Supplementary file

Domain 2: Risk of bias due to deviations from the intended interventions (*effect of assignment to intervention*)

Signalling questions	Comments	Response options
**2.1. Were participants aware of their assigned intervention during the trial?**	Probably Yes.	Y / PY / PN / N / NI
**2.2. Were carers and people delivering the interventions aware of participants' assigned intervention during the trial?**	Probably yes.	Y / PY / PN / N / NI
**2.3. If Y/PY/NI to 2.1 or 2.2: Were there deviations from the intended intervention that arose because of the trial context?**	Probably no. The intervention seems to have been carried out as originally intended. However, it is reported that 65% of participants (n=24/37) have achieved the entire intervention program.	NA / Y / PY / PN / N / NI
**2.4 If Y/PY to 2.3: Were these deviations likely to have affected the outcome?**	NA.	NA / Y / PY / PN / N / NI
**2.5. If Y/PY/NI to 2.4: Were these deviations from intended intervention balanced between groups?**	NA.	NA / Y / PY / PN / N / NI
**2.6 Was an appropriate analysis used to estimate the effect of assignment to intervention?**	Yes. Intention-to-treat (ITT) analyses was conducted.	Y / PY / PN / N / NI
**2.7 If N/PN/NI to 2.6: Was there potential for a substantial impact (on the result) of the failure to analyse participants in the group to which they were randomized?**	NA.	NA / Y / PY / PN / N / NI
**Risk-of-bias judgement**	Low risk of bias	Low / High / Some concerns
Optional: What is the predicted direction of bias due to deviations from intended interventions?	NA.	NA / Favours experimental / Favours comparator / Towards null /Away from null / Unpredictable

Supplementary file

Domain 3: Missing outcome data

Signalling questions	Comments	Response options
**3.1 Were data for this outcome available for all, or nearly all, participants randomized?**	Yes. Four losses to follow-up after 8 weeks out of 78 participants. The Reasons were disclosed. Losses unlikely to affect the results.	Y / PY / PN / N / NI
**3.2 If N/PN/NI to 3.1: Is there evidence that the result was not biased by missing outcome data?**	NA.	NA / Y / PY / PN / N
**3.3 If N/PN to 3.2: Could missingness in the outcome depend on its true value?**	NA.	NA / Y / PY / PN / N / NI
**3.4 If Y/PY/NI to 3.3: Is it likely that missingness in the outcome depended on its true value?**		NA / Y / PY / PN / N / NI
**Risk-of-bias judgement**	Low risk of bias.	Low / High / Some concerns
Optional: What is the predicted direction of bias due to missing outcome data?	NA.	NA / Favours experimental / Favours comparator / Towards null /Away from null / Unpredictable

Supplementary file

Domain 4: Risk of bias in measurement of the outcome

Signalling questions	Comments	Response options
**4.1 Was the method of measuring the outcome inappropriate?**	No.	Y / PY / PN / N / NI
**4.2 Could measurement or ascertainment of the outcome have differed between intervention groups?**	Probably no.	Y / PY / PN / N / NI
**4.3 If N/PN/NI to 4.1 and 4.2: Were outcome assessors aware of the intervention received by study participants?**	Probably yes.	NA / Y / PY / PN / N / NI
**4.4 If Y/PY/NI to 4.3: Could assessment of the outcome have been influenced by knowledge of intervention received?**	Probably No. PHQ-9, as the outcome of this RCT, is a self-assessed measure of depression that is highly relevant and reliable. Therefore, it is unlikely that the evaluator (participant) was influenced by the knowledge of the intervention.	NA / Y / PY / PN / N / NI
**4.5 If Y/PY/NI to 4.4: Is it likely that assessment of the outcome was influenced by knowledge of intervention received?**	NA.	NA / Y / PY / PN / N / NI
**Risk-of-bias judgement**	Low risk of bias.	Low / High / Some concerns
Optional: What is the predicted direction of bias in measurement of the outcome?	NA.	NA / Favours experimental / Favours comparator / Towards null /Away from null / Unpredictable

Supplementary file

Domain 5: Risk of bias in selection of the reported result

Signalling questions	Comments	Response options
**5.1 Were the data that produced this result analysed in accordance with a pre-specified analysis plan that was finalized before unblinded outcome data were available for analysis?**	Yes. This RCT has a pre-designed trial protocol and is registered in the clinical trial registration system. The pre-registered test protocol is consistent with the description in the paper reporting the results of this RCT.	Y / PY / PN / N / NI
**Is the numerical result being assessed likely to have been selected, on the basis of the results, from...**		
**5.2. ... multiple eligible outcome measurements (e.g. scales, definitions, time points) within the outcome domain?**	No.	Y / PY / PN / N / NI
**5.3 ... multiple eligible analyses of the data?**	No.	Y / PY / PN / N / NI
**Risk-of-bias judgement**	Low risk of bias.	Low / High / Some concerns
Optional: What is the predicted direction of bias due to selection of the reported result?	NA.	NA / Favours experimental / Favours comparator / Towards null /Away from null / Unpredictable

Supplementary file

Overall risk of bias

**Risk-of-bias judgement**	Low risk of bias.	Low / High / Some concerns
Optional: What is the overall predicted direction of bias for this outcome?	NA.	NA / Favours experimental / Favours comparator / Towards null /Away from null / Unpredictable

Supplementary file

Study details

**Reference**	Alschuler KN, Altman JK, Ehde DM. Feasibility and acceptability of a single-session, videoconference-delivered group intervention for pain in multiple sclerosis. *Rehabil Psychol*. 2021;66(1):22-30. doi:10.1037/rep0000360

Study design

X Individually-randomized parallel-group trial
☐ Cluster-randomized parallel-group trial
☐ Individually randomized cross-over (or other matched) trial

For the purposes of this assessment, the interventions being compared are defined as

Experimental:	Videoconference-delivered single-session pain intervention	Comparator:	Treatment as usual (TAU)

**Specify which outcome is being assessed for risk of bias**	The Pain Catastrophizing Scale (PCS)
**Specify the numerical result being assessed.** In case of multiple alternative analyses being presented, specify the numeric result (e.g. RR = 1.52 (95% CI 0.83 to 2.77) and/or a reference (e.g. to a table, figure or paragraph) that uniquely defines the result being assessed.	PCS decreased from 20.1 (SD = 7.8), 17.4 (SD = 10.2) to 15.6 (SD = 10.0), 17.3 (SD = 9.3) in the test and TAU groups, respectively.

Is the review team's aim for this result…?

X to assess the effect of *assignment to intervention* (the 'intention-to-treat' effect)
☐ to assess the effect of *adhering to intervention* (the 'per-protocol' effect)

If the aim is to assess the effect of *adhering to intervention*, select the deviations from intended intervention that should be addressed (at least one must be checked):

☐ occurrence of non-protocol interventions
☐ failures in implementing the intervention that could have affected the outcome
☐ non-adherence to their assigned intervention by trial participants

Which of the following sources were <u>obtained</u> to help inform the risk-of-bias assessment? (tick as many as apply)

Supplementary file

X	Journal article(s) with results of the trial
☐	Trial protocol
☐	Statistical analysis plan (SAP)
☐	Non-commercial trial registry record (e.g. ClinicalTrials.gov record)
☐	Company-owned trial registry record (e.g. GSK Clinical Study Register record)
☐	“Grey literature” (e.g. unpublished thesis)
☐	Conference abstract(s) about the trial
☐	Regulatory document (e.g. Clinical Study Report, Drug Approval Package)
☐	Research ethics application
☐	Grant database summary (e.g. NIH RePORTER or Research Councils UK Gateway to Research)
☐	Personal communication with trialist
☐	Personal communication with the sponsor

Supplementary file

Risk of bias assessment

Responses underlined in green are potential markers for low risk of bias, and responses in red are potential markers for a risk of bias. Where questions relate only to sign posts to other questions, no formatting is used.

Domain 1: Risk of bias arising from the randomization process

Signalling questions	Comments	Response options
**1.1 Was the allocation sequence random?**	Yes. "Computer-generated random number sequence."	Y / PY / PN / N / NI
**1.2 Was the allocation sequence concealed until participants were enrolled and assigned to interventions?**	No information.	Y / PY / PN / N / NI
**1.3 Did baseline differences between intervention groups suggest a problem with the randomization process?**	No.	Y / PY / PN / N / NI
**Risk-of-bias judgement**	Some concerns.	Low / High / Some concerns
Optional: What is the predicted direction of bias arising from the randomization process?	Unpredicatable.	NA / Favours experimental / Favours comparator / Towards null /Away from null / Unpredictable

Supplementary file

Domain 2: Risk of bias due to deviations from the intended interventions (*effect of assignment to intervention*)

Signalling questions	Comments	Response options
**2.1. Were participants aware of their assigned intervention during the trial?**	Yes.	Y / PY / PN / N / NI
**2.2. Were carers and people delivering the interventions aware of participants' assigned intervention during the trial?**	Yes.	Y / PY / PN / N / NI
**2.3. If Y/PY/NI to 2.1 or 2.2: Were there deviations from the intended intervention that arose because of the trial context?**	Probably no.	NA / Y / PY / PN / N / NI
**2.4 If Y/PY to 2.3: Were these deviations likely to have affected the outcome?**	NA.	NA / Y / PY / PN / N / NI
**2.5. If Y/PY/NI to 2.4: Were these deviations from intended intervention balanced between groups?**	NA.	NA / Y / PY / PN / N / NI
**2.6 Was an appropriate analysis used to estimate the effect of assignment to intervention?**	No information.	Y / PY / PN / N / NI
**2.7 If N/PN/NI to 2.6: Was there potential for a substantial impact (on the result) of the failure to analyse participants in the group to which they were randomized?**	No. There is no data loss.	NA / Y / PY / PN / N / NI
**Risk-of-bias judgement**	Some concerns.	Low / High / Some concerns
Optional: What is the predicted direction of bias due to deviations from intended interventions?	Unpredictable.	NA / Favours experimental / Favours comparator / Towards null /Away from null / Unpredictable

Supplementary file

Domain 3: Missing outcome data

Signalling questions	Comments	Response options
**3.1 Were data for this outcome available for all, or nearly all, participants randomized?**	Yes.	Y / PY / PN / N / NI
**3.2 If N/PN/NI to 3.1: Is there evidence that the result was not biased by missing outcome data?**	NA.	NA / Y / PY / PN / N
**3.3 If N/PN to 3.2: Could missingness in the outcome depend on its true value?**	NA.<br><br>NA.	NA / Y / PY / PN / N / NI
**3.4 If Y/PY/NI to 3.3: Is it likely that missingness in the outcome depended on its true value?**		NA / Y / PY / PN / N / NI
**Risk-of-bias judgement**	Low risk of bias.	Low / High / Some concerns
Optional: What is the predicted direction of bias due to missing outcome data?	NA.	NA / Favours experimental / Favours comparator / Towards null /Away from null / Unpredictable

Supplementary file

Domain 4: Risk of bias in measurement of the outcome

Signalling questions	Comments	Response options
**4.1 Was the method of measuring the outcome inappropriate?**	Yes.	Y / PY / PN / N / NI
**4.2 Could measurement or ascertainment of the outcome have differed between intervention groups?**	Yes.	Y / PY / PN / N / NI
**4.3 If N/PN/NI to 4.1 and 4.2: Were outcome assessors aware of the intervention received by study participants?**	Yes.	NA / Y / PY / PN / N / NI
**4.4 If Y/PY/NI to 4.3: Could assessment of the outcome have been influenced by knowledge of intervention received?**	Probably no.	NA / Y / PY / PN / N / NI
**4.5 If Y/PY/NI to 4.4: Is it likely that assessment of the outcome was influenced by knowledge of intervention received?**	NA.	NA / Y / PY / PN / N / NI
**Risk-of-bias judgement**	Low risk of bias.	Low / High / Some concerns
Optional: What is the predicted direction of bias in measurement of the outcome?	NA.	NA / Favours experimental / Favours comparator / Towards null /Away from null / Unpredictable

Supplementary file

Domain 5: Risk of bias in selection of the reported result

Signalling questions	Comments	Response options
**5.1 Were the data that produced this result analysed in accordance with a pre-specified analysis plan that was finalized before unblinded outcome data were available for analysis?**	No information.	Y / PY / PN / N / NI
**Is the numerical result being assessed likely to have been selected, on the basis of the results, from...**		
**5.2. ... multiple eligible outcome measurements (e.g. scales, definitions, time points) within the outcome domain?**	No information.	Y / PY / PN / N / NI
**5.3 ... multiple eligible analyses of the data?**	No information.	Y / PY / PN / N / NI
**Risk-of-bias judgement**	Some concerns.	Low / High / Some concerns
Optional: What is the predicted direction of bias due to selection of the reported result?	Unpredictable.	NA / Favours experimental / Favours comparator / Towards null /Away from null / Unpredictable

Supplementary file

Overall risk of bias

**Risk-of-bias judgement**	Some concerns.	Low / High / Some concerns
Optional: What is the overall predicted direction of bias for this outcome?	Unpredictable.	NA / Favours experimental / Favours comparator / Towards null /Away from null / Unpredictable

Supplementary file

Study details

**Reference**	Bogosian A, Hurt CS, Hindle JV, et al. Acceptability and Feasibility of a Mindfulness Intervention Delivered via Videoconferencing for People With Parkinson's [published online ahead of print, 2021 Jan 28]. *J Geriatr Psychiatry Neurol*. 2021;891988720988901. doi:10.1177/0891988720988901

Study design

X Individually-randomized parallel-group trial
☐ Cluster-randomized parallel-group trial
☐ Individually randomized cross-over (or other matched) trial

For the purposes of this assessment, the interventions being compared are defined as

Experimental: the Skype delivered mindfulness Comparator: Wait-list control

**Specify which outcome is being assessed for risk of bias**	Depression on hospital anxiety and depression scale (HADS).
**Specify the numerical result being assessed.** In case of multiple alternative analyses being presented, specify the numeric result (e.g. RR = 1.52 (95% CI 0.83 to 2.77) and/or a reference (e.g. to a table, figure or paragraph) that uniquely defines the result being assessed.	Table 3. to assess the effect of *adhering to intervention* (the 'per-protocol' effect)

Is the review team's aim for this result…?

X to assess the effect of *assignment to intervention* (the 'intention-to-treat' effect)
☐ to assess the effect of *adhering to intervention* (the 'per-protocol' effect)

If the aim is to assess the effect of *adhering to intervention*, select the deviations from intended intervention that should be addressed (at least one must be checked):

☐ occurrence of non-protocol interventions
☐ failures in implementing the intervention that could have affected the outcome
☐ non-adherence to their assigned intervention by trial participants

Which of the following sources were <u>obtained</u> to help inform the risk-of-bias assessment? (tick as many as apply)

Supplementary file

X	Journal article(s) with results of the trial
☐	Trial protocol
☐	Statistical analysis plan (SAP)
X	Non-commercial trial registry record (e.g. ClinicalTrials.gov record)
☐	Company-owned trial registry record (e.g. GSK Clinical Study Register record)
☐	"Grey literature" (e.g. unpublished thesis)
☐	Conference abstract(s) about the trial
☐	Regulatory document (e.g. Clinical Study Report, Drug Approval Package)
☐	Research ethics application
☐	Grant database summary (e.g. NIH RePORTER or Research Councils UK Gateway to Research)
☐	Personal communication with trialist
☐	Personal communication with the sponsor

Supplementary file

Risk of bias assessment

Responses underlined in green are potential markers for low risk of bias, and responses in red are potential markers for a risk of bias. Where questions relate only to sign posts to other questions, no formatting is used.

Domain 1: Risk of bias arising from the randomization process

Signalling questions	Comments	Response options
**1.1 Was the allocation sequence random?**	Yes. "An independent service at the King's College Mental Health and Neuroscience Clinical Trials Unit (CTU) handled the randomisation, using fixed block sizes of two".	Y / PY / PN / N / NI
**1.2 Was the allocation sequence concealed until participants were enrolled and assigned to interventions?**	Yes. Central allocation.	Y / PY / PN / N / NI
**1.3 Did baseline differences between intervention groups suggest a problem with the randomization process?**	No.	Y / PY / PN / N / NI
**Risk-of-bias judgement**	Low risk of bias.	Low / High / Some concerns
Optional: What is the predicted direction of bias arising from the randomization process?	NA.	NA / Favours experimental / Favours comparator / Towards null /Away from null / Unpredictable

Supplementary file

Domain 2: Risk of bias due to deviations from the intended interventions (*effect of assignment to intervention*)

Signalling questions	Comments	Response options
**2.1. Were participants aware of their assigned intervention during the trial?**	Yes.	Y / PY / PN / N / NI
**2.2. Were carers and people delivering the interventions aware of participants' assigned intervention during the trial?**	Yes.	Y / PY / PN / N / NI
**2.3. If Y/PY/NI to 2.1 or 2.2: Were there deviations from the intended intervention that arose because of the trial context?**	No.	NA / Y / PY / PN / N / NI
**2.4 If Y/PY to 2.3: Were these deviations likely to have affected the outcome?**	NA.	NA / Y / PY / PN / N / NI
**2.5. If Y/PY/NI to 2.4: Were these deviations from intended intervention balanced between groups?**	NA.	NA / Y / PY / PN / N / NI
**2.6 Was an appropriate analysis used to estimate the effect of assignment to intervention?**	Yes. In the RCT, ITT analyses were conducted.	Y / PY / PN / N / NI
**2.7 If N/PN/NI to 2.6: Was there potential for a substantial impact (on the result) of the failure to analyse participants in the group to which they were randomized?**	NA.	NA / Y / PY / PN / N / NI
**Risk-of-bias judgement**	Low risk of bias.	Low / High / Some concerns
Optional: What is the predicted direction of bias due to deviations from intended interventions?	NA.	NA / Favours experimental / Favours comparator / Towards null /Away from null / Unpredictable

Supplementary file

Domain 3: Missing outcome data

Signalling questions	Comments	Response options
**3.1 Were data for this outcome available for all, or nearly all, participants randomized?**	Yes. Data from all participants are used for analysis in this RCT.	Y / PY / PN / N / NI
**3.2 If N/PN/NI to 3.1: Is there evidence that the result was not biased by missing outcome data?**	NA.	NA / Y / PY / PN / N
**3.3 If N/PN to 3.2: Could missingness in the outcome depend on its true value?**	NA.	NA / Y / PY / PN / N / NI
**3.4 If Y/PY/NI to 3.3: Is it likely that missingness in the outcome depended on its true value?**		NA / Y / PY / PN / N / NI
**Risk-of-bias judgement**	Low risk of bias.	Low / High / Some concerns
Optional: What is the predicted direction of bias due to missing outcome data?	NA.	NA / Favours experimental / Favours comparator / Towards null /Away from null / Unpredictable

Supplementary file

Domain 4: Risk of bias in measurement of the outcome

Signalling questions	Comments	Response options
**4.1 Was the method of measuring the outcome inappropriate?**	No.	Y / PY / PN / N / NI
**4.2 Could measurement or ascertainment of the outcome have differed between intervention groups?**	Probably No.	Y / PY / PN / N / NI
**4.3 If N/PN/NI to 4.1 and 4.2: Were outcome assessors aware of the intervention received by study participants?**	Yes.	NA / Y / PY / PN / N / NI
**4.4 If Y/PY/NI to 4.3: Could assessment of the outcome have been influenced by knowledge of intervention received?**	Probably No. HADS is a responsive, valid and reliable self-assessment questionnaire. It is unlikely that the answer will be distorted just because the assessor (participant) knows the intervention.	NA / Y / PY / PN / N / NI
**4.5 If Y/PY/NI to 4.4: Is it likely that assessment of the outcome was influenced by knowledge of intervention received?**	NA.	NA / Y / PY / PN / N / NI
**Risk-of-bias judgement**	Low risk of bias.	Low / High / Some concerns
Optional: What is the predicted direction of bias in measurement of the outcome?	NA.	NA / Favours experimental / Favours comparator / Towards null /Away from null / Unpredictable

Supplementary file

Domain 5: Risk of bias in selection of the reported result

Signalling questions	Comments	Response options
**5.1 Were the data that produced this result analysed in accordance with a pre-specified analysis plan that was finalized before unblinded outcome data were available for analysis?**	Yes. “Ethical approval was obtained from the City, University of London Psychology Ethics Committee (reference: PSYETH (S/F) 15/16 112) and registered with ClinicalTrials.gov (NCT02683330) in January 2016.”	Y / PY / PN / N / NI
**Is the numerical result being assessed likely to have been selected, on the basis of the results, from...**		
**5.2. ... multiple eligible outcome measurements (e.g. scales, definitions, time points) within the outcome domain?**	No.	Y / PY / PN / N / NI
**5.3 ... multiple eligible analyses of the data?**	No. “2 (group: Mindfulness, Wait-list) x 4 (time: baseline, mid-intervention, post-intervention, follow-up) mixed ANOVAs were conducted to see the effect of group allocation (between-subjects factor) and time (within-subjects factor) on both the primary and secondary outcome measures”	Y / PY / PN / N / NI
**Risk-of-bias judgement**	Low risk of bias.	Low / High / Some concerns
Optional: What is the predicted direction of bias due to selection of the reported result?	NA.	NA / Favours experimental / Favours comparator / Towards null /Away from null / Unpredictable

Supplementary file

Overall risk of bias

**Risk-of-bias judgement**	Low risk of bias.	Low / High / Some concerns
Optional: What is the overall predicted direction of bias for this outcome?	NA.	NA / Favours experimental / Favours comparator / Towards null /Away from null / Unpredictable

Supplementary file

Study details

**Reference**	Choi NG, Hegel MT, Marti N, Marinucci ML, Sirrianni L, Bruce ML. Telehealth problem-solving therapy for depressed low-income homebound older adults. Am J Geriatr Psychiatry. 2014;22(3):263-271.

Study design

X Individually-randomized parallel-group trial
☐ Cluster-randomized parallel-group trial
☐ Individually randomized cross-over (or other matched) trial

For the purposes of this assessment, the interventions being compared are defined as

Experimental:	Telehealth delivery of problem-solving therapy.	Comparator:	Telephone support calls.

**Specify which outcome is being assessed for risk of bias**	Hamilton Depression rating Scale (HAM-D)
**Specify the numerical result being assessed.** In case of multiple alternative analyses being presented, specify the numeric result (e.g. RR = 1.52 (95% CI 0.83 to 2.77) and/or a reference (e.g. to a table, figure or paragraph) that uniquely defines the result being assessed.	Table 3 shows that the group differences in predicted mean HAMD scores at 12-week follow-up were significant between tele-PST participants and telephone support call participants (Tele-PST 13.92 (SE=1.18) vs. control group 19.16 (SE=1.26); t=-3.03; df=233.56; P=.003)

Is the review team's aim for this result…?

X to assess the effect of *assignment to intervention* (the 'intention-to-treat' effect)
☐ to assess the effect of *adhering to intervention* (the 'per-protocol' effect)

If the aim is to assess the effect of *adhering to intervention*, select the deviations from intended intervention that should be addressed (at least one must be checked):

☐ occurrence of non-protocol interventions
☐ failures in implementing the intervention that could have affected the outcome
☐ non-adherence to their assigned intervention by trial participants

Supplementary file

Which of the following sources were obtained to help inform the risk-of-bias assessment? (tick as many as apply)

X Journal article(s) with results of the trial
☐ Trial protocol
☐ Statistical analysis plan (SAP)
☐ Non-commercial trial registry record (e.g. ClinicalTrials.gov record)
☐ Company-owned trial registry record (e.g. GSK Clinical Study Register record)
☐ "Grey literature" (e.g. unpublished thesis)
☐ Conference abstract(s) about the trial
☐ Regulatory document (e.g. Clinical Study Report, Drug Approval Package)
☐ Research ethics application
☐ Grant database summary (e.g. NIH RePORTER or Research Councils UK Gateway to Research)
☐ Personal communication with trialist
☐ Personal communication with the sponsor

Supplementary file

Risk of bias assessment

Responses underlined in green are potential markers for low risk of bias, and responses in red are potential markers for a risk of bias. Where questions relate only to sign posts to other questions, no formatting is used.

Domain 1: Risk of bias arising from the randomization process

Signalling questions	Comments	Response options
**1.1 Was the allocation sequence random?**	No information.	Y / PY / PN / N / NI
**1.2 Was the allocation sequence concealed until participants were enrolled and assigned to interventions?**	No information.	Y / PY / PN / N / NI
**1.3 Did baseline differences between intervention groups suggest a problem with the randomization process?**	No.	Y / PY / PN / N / NI
**Risk-of-bias judgement**	Some concerns.	Low / High / Some concerns
Optional: What is the predicted direction of bias arising from the randomization process?	Unpredictable.	NA / Favours experimental / Favours comparator / Towards null /Away from null / Unpredictable

Supplementary file

Domain 2: Risk of bias due to deviations from the intended interventions (*effect of assignment to intervention*)

Signalling questions	Comments	Response options
**2.1. Were participants aware of their assigned intervention during the trial?**	No information.	Y / PY / PN / N / NI
**2.2. Were carers and people delivering the interventions aware of participants' assigned intervention during the trial?**	No information.	Y / PY / PN / N / NI
**2.3. If Y/PY/NI to 2.1 or 2.2: Were there deviations from the intended intervention that arose because of the trial context?**	Probably no.	NA / Y / PY / PN / N / NI
**2.4 If Y/PY to 2.3: Were these deviations likely to have affected the outcome?**	NA.	NA / Y / PY / PN / N / NI
**2.5. If Y/PY/NI to 2.4: Were these deviations from intended intervention balanced between groups?**	NA.	NA / Y / PY / PN / N / NI
**2.6 Was an appropriate analysis used to estimate the effect of assignment to intervention?**	Yes. In the RCT, ITT analyses were conducted.	Y / PY / PN / N / NI
**2.7 If N/PN/NI to 2.6: Was there potential for a substantial impact (on the result) of the failure to analyse participants in the group to which they were randomized?**	NA.	NA / Y / PY / PN / N / NI
**Risk-of-bias judgement**	Low risk of bias.	Low / High / Some concerns
Optional: What is the predicted direction of bias due to deviations from intended interventions?	NA.	NA / Favours experimental / Favours comparator / Towards null /Away from null / Unpredictable

Supplementary file

Domain 3: Missing outcome data

Signalling questions	Comments	Response options
**3.1 Were data for this outcome available for all, or nearly all, participants randomized?**	Probably yes. Researchers in the RCT have succeeded in collecting all the data of about 90% (n=69/76) of the participants. In addition, they use data from almost all participants in this RCT for analysis	Y / PY / PN / N / NI
**3.2 If N/PN/NI to 3.1: Is there evidence that the result was not biased by missing outcome data?**	NA.	NA / Y / PY / PN / N
**3.3 If N/PN to 3.2: Could missingness in the outcome depend on its true value?**	NA.	NA / Y / PY / PN / N / NI
**3.4 If Y/PY/NI to 3.3: Is it likely that missingness in the outcome depended on its true value?**	NA.	NA / Y / PY / PN / N / NI
**Risk-of-bias judgement**	Low risk of bias.	Low / High / Some concerns
Optional: What is the predicted direction of bias due to missing outcome data?	NA.	NA / Favours experimental / Favours comparator / Towards null /Away from null / Unpredictable

Supplementary file

Domain 4: Risk of bias in measurement of the outcome

Signalling questions	Comments	Response options
**4.1 Was the method of measuring the outcome inappropriate?**	Probably no.	Y / PY / PN / N / NI
**4.2 Could measurement or ascertainment of the outcome have differed between intervention groups?**	Probably no.	Y / PY / PN / N / NI
**4.3 If N/PN/NI to 4.1 and 4.2: Were outcome assessors aware of the intervention received by study participants?**	No information.	NA / Y / PY / PN / N / NI
**4.4 If Y/PY/NI to 4.3: Could assessment of the outcome have been influenced by knowledge of intervention received?**	Probably no. HAM-D is a responsive depressive symptom rating scale performed by trained professionals. The validity and reliability of the scale is so strong that knowledge of the intervention is unlikely to distort the outcome.	NA / Y / PY / PN / N / NI
**4.5 If Y/PY/NI to 4.4: Is it likely that assessment of the outcome was influenced by knowledge of intervention received?**		NA / Y / PY / PN / N / NI
**Risk-of-bias judgement**	Low risk of bias.	Low / High / Some concerns
Optional: What is the predicted direction of bias in measurement of the outcome?	AN.	NA / Favours experimental / Favours comparator / Towards null /Away from null / Unpredictable

Supplementary file

Domain 5: Risk of bias in selection of the reported result

Signalling questions	Comments	Response options
**5.1 Were the data that produced this result analysed in accordance with a pre-specified analysis plan that was finalized before unblinded outcome data were available for analysis?**	No information.	Y / PY / PN / N / NI
**Is the numerical result being assessed likely to have been selected, on the basis of the results, from...**		
**5.2. ... multiple eligible outcome measurements (e.g. scales, definitions, time points) within the outcome domain?**	No. There is only one possible way in which the outcome domain can be measured (hence there is no opportunity to select from multiple measures).	Y / PY / PN / N / NI
**5.3 ... multiple eligible analyses of the data?**	No.	Y / PY / PN / N / NI
**Risk-of-bias judgement**	Some concerns.	Low / High / Some concerns
Optional: What is the predicted direction of bias due to selection of the reported result?	Towards null.	NA / Favours experimental / Favours comparator / Towards null /Away from null / Unpredictable

Supplementary file

Overall risk of bias

**Risk-of-bias judgement**	Some concerns.	Low / High / Some concerns
Optional: What is the overall predicted direction of bias for this outcome?	Towards null.	NA / Favours experimental / Favours comparator / Towards null /Away from null / Unpredictable

Supplementary file

Study details

**Reference**	Choi NG, Pepin R, Marti CN, Stevens CJ, Bruce ML. Improving Social Connectedness for Homebound Older Adults: Randomized Controlled Trial of Tele-Delivered Behavioral Activation Versus Tele-Delivered Friendly Visits. Am J Geriatr Psychiatry. 2020a;28(7):698-708.

Study design

X Individually-randomized parallel-group trial
☐ Cluster-randomized parallel-group trial
☐ Individually randomized cross-over (or other matched) trial

For the purposes of this assessment, the interventions being compared are defined as

Experimental:	Tele-behavioral activation (tele-BA)	Comparator:	Tele-friendly visits (Tele-FV)

**Specify which outcome is being assessed for risk of bias**	PHQ-9
**Specify the numerical result being assessed.** In case of multiple alternative analyses being presented, specify the numeric result (e.g. RR = 1.52 (95% CI 0.83 to 2.77) and/or a reference (e.g. to a table, figure or paragraph) that uniquely defines the result being assessed.	"Compared to Tele-FV participants, Tele-BA participants had greater increase in depression (t [82] = -3.46, p = 0.001), and disability (t [81] = -2.29, p = 0.025)."

Is the review team's aim for this result…?

X to assess the effect of *assignment to intervention* (the 'intention-to-treat' effect)
☐ to assess the effect of *adhering to intervention* (the 'per-protocol' effect)

If the aim is to assess the effect of *adhering to intervention*, select the deviations from intended intervention that should be addressed (at least one must be checked):

☐ occurrence of non-protocol interventions
☐ failures in implementing the intervention that could have affected the outcome
☐ non-adherence to their assigned intervention by trial participants

Supplementary file

Which of the following sources were obtained to help inform the risk-of-bias assessment? (tick as many as apply)	
X	Journal article(s) with results of the trial
☐	Trial protocol
☐	Statistical analysis plan (SAP)
☐	Non-commercial trial registry record (e.g. ClinicalTrials.gov record)
☐	Company-owned trial registry record (e.g. GSK Clinical Study Register record)
☐	“Grey literature” (e.g. unpublished thesis)
☐	Conference abstract(s) about the trial
☐	Regulatory document (e.g. Clinical Study Report, Drug Approval Package)
☐	Research ethics application
☐	Grant database summary (e.g. NIH RePORTER or Research Councils UK Gateway to Research)
☐	Personal communication with trialist
☐	Personal communication with the sponsor

Supplementary file

Risk of bias assessment

Responses underlined in green are potential markers for low risk of bias, and responses in red are potential markers for a risk of bias. Where questions relate only to sign posts to other questions, no formatting is used.

Domain 1: Risk of bias arising from the randomization process

Signalling questions	Comments	Response options
**1.1 Was the allocation sequence random?**	No information.	Y / PY / PN / N / NI
**1.2 Was the allocation sequence concealed until participants were enrolled and assigned to interventions?**	No information.	Y / PY / PN / N / NI
**1.3 Did baseline differences between intervention groups suggest a problem with the randomization process?**	Probably no.	Y / PY / PN / N / NI
**Risk-of-bias judgement**	Some concerns.	Low / High / Some concerns
Optional: What is the predicted direction of bias arising from the randomization process?	Unpredictable.	NA / Favours experimental / Favours comparator / Towards null /Away from null / Unpredictable

Supplementary file

Domain 2: Risk of bias due to deviations from the intended interventions (*effect of assignment to intervention*)

Signalling questions	Comments	Response options
**2.1. Were participants aware of their assigned intervention during the trial?**	Probably no. The control group received active control intervention and was probably unaware of the assigned intervention intent.	Y / PY / PN / N / NI
**2.2. Were carers and people delivering the interventions aware of participants' assigned intervention during the trial?**	Probably yes. The therapists who provided the intervention were probably aware of the intent of this study.	Y / PY / PN / N / NI
**2.3. If Y/PY/NI to 2.1 or 2.2: Were there deviations from the intended intervention that arose because of the trial context?**	Probably no.	NA / Y / PY / PN / N / NI
**2.4 If Y/PY to 2.3: Were these deviations likely to have affected the outcome?**	NA.	NA / Y / PY / PN / N / NI
**2.5. If Y/PY/NI to 2.4: Were these deviations from intended intervention balanced between groups?**	NA.	NA / Y / PY / PN / N / NI
**2.6 Was an appropriate analysis used to estimate the effect of assignment to intervention?**	Yes. Intention-to-treat analysis was performed.	Y / PY / PN / N / NI
**2.7 If N/PN/NI to 2.6: Was there potential for a substantial impact (on the result) of the failure to analyse participants in the group to which they were randomized?**	NA.	NA / Y / PY / PN / N / NI
**Risk-of-bias judgement**	Low risk of bias.	Low / High / Some concerns
Optional: What is the predicted direction of bias due to deviations from intended interventions?	NA.	NA / Favours experimental / Favours comparator / Towards null /Away from null / Unpredictable

Supplementary file

Domain 3: Missing outcome data

Signalling questions	Comments	Response options
**3.1 Were data for this outcome available for all, or nearly all, participants randomized?**	Probably yes. This RCT collected all data for about 91% of the participants (n = 81/89).	Y / PY / PN / N / NI
**3.2 If N/PN/NI to 3.1: Is there evidence that the result was not biased by missing outcome data?**	NA.	NA / Y / PY / PN / N
**3.3 If N/PN to 3.2: Could missingness in the outcome depend on its true value?**	NA.	NA / Y / PY / PN / N / NI
**3.4 If Y/PY/NI to 3.3: Is it likely that missingness in the outcome depended on its true value?**		NA / Y / PY / PN / N / NI
**Risk-of-bias judgement**	Low risk of bias.	Low / High / Some concerns
Optional: What is the predicted direction of bias due to missing outcome data?	NA.	NA / Favours experimental / Favours comparator / Towards null /Away from null / Unpredictable

Supplementary file

Domain 4: Risk of bias in measurement of the outcome

Signalling questions	Comments	Response options
**4.1 Was the method of measuring the outcome inappropriate?**	Yes.	Y / PY / PN / N / NI
**4.2 Could measurement or ascertainment of the outcome have differed between intervention groups?**	No.	Y / PY / PN / N / NI
**4.3 If N/PN/NI to 4.1 and 4.2: Were outcome assessors aware of the intervention received by study participants?**	Probably yes.	NA / Y / PY / PN / N / NI
**4.4 If Y/PY/NI to 4.3: Could assessment of the outcome have been influenced by knowledge of intervention received?**	Probably no. Since the researchers are using PHQ-9, which has been proven to be reliable and valid, there is probably no impact on the assessment results.	NA / Y / PY / PN / N / NI
**4.5 If Y/PY/NI to 4.4: Is it likely that assessment of the outcome was influenced by knowledge of intervention received?**	NA.	NA / Y / PY / PN / N / NI
**Risk-of-bias judgement**	Low risk of bias.	Low / High / Some concerns
Optional: What is the predicted direction of bias in measurement of the outcome?	NA.	NA / Favours experimental / Favours comparator / Towards null /Away from null / Unpredictable

Supplementary file

Domain 5: Risk of bias in selection of the reported result

Signalling questions	Comments	Response options
**5.1 Were the data that produced this result analysed in accordance with a pre-specified analysis plan that was finalized before unblinded outcome data were available for analysis?**	No Information.	Y / PY / PN / N / NI
**Is the numerical result being assessed likely to have been selected, on the basis of the results, from...**		
**5.2. ... multiple eligible outcome measurements (e.g. scales, definitions, time points) within the outcome domain?**	No. In this RCT, depressive symptoms were measured only with PHQ-9.	Y / PY / PN / N / NI
**5.3 ... multiple eligible analyses of the data?**	No. There is only one possible way in which the outcome measurement can be analysed (hence there is no opportunity to select from multiple analyses).	Y / PY / PN / N / NI
**Risk-of-bias judgement**	Some concerns.	Low / High / Some concerns
Optional: What is the predicted direction of bias due to selection of the reported result?	Unpredictable.	NA / Favours experimental / Favours comparator / Towards null /Away from null / Unpredictable

Supplementary file

Overall risk of bias

**Risk-of-bias judgement**	Some concerns	Low / High / Some concerns
Optional: What is the overall predicted direction of bias for this outcome?	Unpredicatable.	NA / Favours experimental / Favours comparator / Towards null /Away from null / Unpredictable

Supplementary file

Study details

**Reference**	Choi NG, Marti CN, Wilson NL, Chen GJ, Sirrianni L, Hegel MT, et al. Effect of Telehealth Treatment by Lay Counselors vs by Clinicians on Depressive Symptoms Among Older Adults Who Are Homebound: A Randomized Clinical Trial. JAMA Netw Open. 2020b;3(8):e2015648.

Study design

X Individually-randomized parallel-group trial

☐ Cluster-randomized parallel-group trial

☐ Individually randomized cross-over (or other matched) trial

For the purposes of this assessment, the interventions being compared are defined as

Experimental:	Videoconference behavioral activation (tele-BA), videoconference problem-solving therapy (tele-PST)	Comparator:	Attention control (AC)

**Specify which outcome is being assessed for risk of bias**	24-itme Hamilton Depression Rating Scale (HAMD)
**Specify the numerical result being assessed.** In case of multiple alternative analyses being presented, specify the numeric result (e.g. RR = 1.52 (95% CI 0.83 to 2.77) and/or a reference (e.g. to a table, figure or paragraph) that uniquely defines the result being assessed.	"Compared with participants in the AC group, participants in the tele-BA and tele-PST groups had significantly higher response and remission rates and medium to large effect sizes (tele-BA: raw growth modeling analysis d = 0.62 [95% CI, 0.35 to 0.89]; P < .001; tele-PST: raw growth modeling analysis d = 1.00 [95% CI, 0.73 to 1.26]; P < .001) for HAMD scores."

Is the review team's aim for this result…?

X to assess the effect of *assignment to intervention* (the 'intention-to-treat' effect)

☐ to assess the effect of *adhering to intervention* (the 'per-protocol' effect)

If the aim is to assess the effect of *adhering to intervention*, select the deviations from intended intervention that should be addressed (at least one must be checked):

☐ occurrence of non-protocol interventions

☐ failures in implementing the intervention that could have affected the outcome

☐	non-adherence to their assigned intervention by trial participants

Which of the following sources were <u>obtained</u> to help inform the risk-of-bias assessment? (tick as many as apply)

X	Journal article(s) with results of the trial
X	Trial protocol
X	Statistical analysis plan (SAP)
☐	Non-commercial trial registry record (e.g. ClinicalTrials.gov record)
☐	Company-owned trial registry record (e.g. GSK Clinical Study Register record)
☐	"Grey literature" (e.g. unpublished thesis)
☐	Conference abstract(s) about the trial
☐	Regulatory document (e.g. Clinical Study Report, Drug Approval Package)
☐	Research ethics application
☐	Grant database summary (e.g. NIH RePORTER or Research Councils UK Gateway to Research)
☐	Personal communication with trialist
☐	Personal communication with the sponsor

Supplementary file

Risk of bias assessment

Responses underlined in green are potential markers for low risk of bias, and responses in red are potential markers for a risk of bias. Where questions relate only to sign posts to other questions, no formatting is used.

Domain 1: Risk of bias arising from the randomization process

Signalling questions	Comments	Response options
**1.1 Was the allocation sequence random?**	Yes. "A random assignment sequence generated by the project's biostatistician (C.N.M.) was used."	Y / PY / PN / N / NI
**1.2 Was the allocation sequence concealed until participants were enrolled and assigned to interventions?**	Yes. Central allocation.	Y / PY / PN / N / NI
**1.3 Did baseline differences between intervention groups suggest a problem with the randomization process?**	No.	Y / PY / PN / N / NI
**Risk-of-bias judgement**	Low risk of bias.	Low / High / Some concerns
Optional: What is the predicted direction of bias arising from the randomization process?	NA.	NA / Favours experimental / Favours comparator / Towards null /Away from null / Unpredictable

Supplementary file

Domain 2: Risk of bias due to deviations from the intended interventions (*effect of assignment to intervention*)

Signalling questions	Comments	Response options
**2.1. Were participants aware of their assigned intervention during the trial?**	Probably No. This RCT provided a psychological placebo with AC to the control group.	Y / PY / PN / N / NI
**2.2. Were carers and people delivering the interventions aware of participants' assigned intervention during the trial?**	Probably yes.	Y / PY / PN / N / NI
**2.3. If Y/PY/NI to 2.1 or 2.2: Were there deviations from the intended intervention that arose because of the trial context?**	Probably no.	NA / Y / PY / PN / N / NI
**2.4 If Y/PY to 2.3: Were these deviations likely to have affected the outcome?**	NA.	NA / Y / PY / PN / N / NI
**2.5. If Y/PY/NI to 2.4: Were these deviations from intended intervention balanced between groups?**	NA.	NA / Y / PY / PN / N / NI
**2.6 Was an appropriate analysis used to estimate the effect of assignment to intervention?**	Yes. ITT analyses were performed.	Y / PY / PN / N / NI
**2.7 If N/PN/NI to 2.6: Was there potential for a substantial impact (on the result) of the failure to analyse participants in the group to which they were randomized?**	NA.	NA / Y / PY / PN / N / NI
**Risk-of-bias judgement**	Low risk of bias.	Low / High / Some concerns
Optional: What is the predicted direction of bias due to deviations from intended interventions?	NA.	NA / Favours experimental / Favours comparator / Towards null /Away from null / Unpredictable

Supplementary file

Domain 3: Missing outcome data

Signalling questions	Comments	Response options
**3.1 Were data for this outcome available for all, or nearly all, participants randomized?**	Probably yes. This RCT collected all data for about 95% of the participants (n = 278/295).	Y / PY / PN / N / NI
**3.2 If N/PN/NI to 3.1: Is there evidence that the result was not biased by missing outcome data?**	NA.	NA / Y / PY / PN / N
**3.3 If N/PN to 3.2: Could missingness in the outcome depend on its true value?**	NA.	NA / Y / PY / PN / N / NI
**3.4 If Y/PY/NI to 3.3: Is it likely that missingness in the outcome depended on its true value?**		NA / Y / PY / PN / N / NI
**Risk-of-bias judgement**	Low risk of bias.	Low / High / Some concerns
Optional: What is the predicted direction of bias due to missing outcome data?	NA.	NA / Favours experimental / Favours comparator / Towards null /Away from null / Unpredictable

Supplementary file

Domain 4: Risk of bias in measurement of the outcome

Signalling questions	Comments	Response options
**4.1 Was the method of measuring the outcome inappropriate?**	No.	Y / PY / PN / N / NI
**4.2 Could measurement or ascertainment of the outcome have differed between intervention groups?**	No.	Y / PY / PN / N / NI
**4.3 If N/PN/NI to 4.1 and 4.2: Were outcome assessors aware of the intervention received by study participants?**	Yes.	NA / Y / PY / PN / N / NI
**4.4 If Y/PY/NI to 4.3: Could assessment of the outcome have been influenced by knowledge of intervention received?**	No. Assessors were not informed of study hypotheses in RCT.	NA / Y / PY / PN / N / NI
**4.5 If Y/PY/NI to 4.4: Is it likely that assessment of the outcome was influenced by knowledge of intervention received?**		NA / Y / PY / PN / N / NI
**Risk-of-bias judgement**	Low risk of bias.	Low / High / Some concerns
Optional: What is the predicted direction of bias in measurement of the outcome?	NA.	NA / Favours experimental / Favours comparator / Towards null /Away from null / Unpredictable

Supplementary file

Domain 5: Risk of bias in selection of the reported result

Signalling questions	Comments	Response options
**5.1 Were the data that produced this result analysed in accordance with a pre-specified analysis plan that was finalized before unblinded outcome data were available for analysis?**	Yes. The protocol of this RCT has been submitted in advance.	Y / PY / PN / N / NI
**Is the numerical result being assessed likely to have been selected, on the basis of the results, from...**		
**5.2. ... multiple eligible outcome measurements (e.g. scales, definitions, time points) within the outcome domain?**	No. There is clear evidence that all eligible reported results for the outcome domain correspond to all intended outcome measurements.	Y / PY / PN / N / NI
**5.3 ... multiple eligible analyses of the data?**	No. There is clear evidence that all eligible reported results for the outcome domain correspond to all intended outcome measurements.	Y / PY / PN / N / NI
**Risk-of-bias judgement**	Low risk of bias.	Low / High / Some concerns
Optional: What is the predicted direction of bias due to selection of the reported result?	NA.	NA / Favours experimental / Favours comparator / Towards null /Away from null / Unpredictable

Supplementary file

Overall risk of bias

**Risk-of-bias judgement**	Low risk of bias.	Low / High / Some concerns
Optional: What is the overall predicted direction of bias for this outcome?	NA.	NA / Favours experimental / Favours comparator / Towards null /Away from null / Unpredictable

Supplementary file

Study details

**Reference**	Demiris G, Oliver DP, Washington K, Pike K. A Problem-Solving Intervention for Hospice Family Caregivers: A Randomized Clinical Trial. J Am Geriatr Soc. 2019;67(7):1345-1352.

Study design

X Individually-randomized parallel-group trial

☐ Cluster-randomized parallel-group trial

☐ Individually randomized cross-over (or other matched) trial

For the purposes of this assessment, the interventions being compared are defined as

Experimental: PSI via videoconferencing Comparator: AC

**Specify which outcome is being assessed for risk of bias**	The Generalized Anxiety Disorder 7-item (GAD-7)
**Specify the numerical result being assessed.** In case of multiple alternative analyses being presented, specify the numeric result (e.g. RR = 1.52 (95% CI 0.83 to 2.77) and/or a reference (e.g. to a table, figure or paragraph) that uniquely defines the result being assessed.	There were no differences between conditions.

Is the review team's aim for this result…?

X to assess the effect of *assignment to intervention* (the 'intention-to-treat' effect)

☐ to assess the effect of *adhering to intervention* (the 'per-protocol' effect)

If the aim is to assess the effect of *adhering to intervention*, select the deviations from intended intervention that should be addressed (at least one must be checked):

☐ occurrence of non-protocol interventions

☐ failures in implementing the intervention that could have affected the outcome

☐ non-adherence to their assigned intervention by trial participants

Which of the following sources were obtained to help inform the risk-of-bias assessment? (tick as many as apply)

Supplementary file

X	Journal article(s) with results of the trial
☐	Trial protocol
☐	Statistical analysis plan (SAP)
X	Non-commercial trial registry record (e.g. ClinicalTrials.gov record)
☐	Company-owned trial registry record (e.g. GSK Clinical Study Register record)
☐	"Grey literature" (e.g. unpublished thesis)
☐	Conference abstract(s) about the trial
☐	Regulatory document (e.g. Clinical Study Report, Drug Approval Package)
☐	Research ethics application
☐	Grant database summary (e.g. NIH RePORTER or Research Councils UK Gateway to Research)
☐	Personal communication with trialist
☐	Personal communication with the sponsor

Supplementary file

Risk of bias assessment

Responses underlined in green are potential markers for low risk of bias, and responses in red are potential markers for a risk of bias. Where questions relate only to sign posts to other questions, no formatting is used.

Domain 1: Risk of bias arising from the randomization process

Signalling questions	Comments	Response options
**1.1 Was the allocation sequence random?**	Yes. "A block randomization approach was used."	Y / PY / PN / N / NI
**1.2 Was the allocation sequence concealed until participants were enrolled and assigned to interventions?**	Yes. Central allocation.	Y / PY / PN / N / NI
**1.3 Did baseline differences between intervention groups suggest a problem with the randomization process?**	No.	Y / PY / PN / N / NI
**Risk-of-bias judgement**	Low risk of bias.	Low / High / Some concerns
Optional: What is the predicted direction of bias arising from the randomization process?	NA.	NA / Favours experimental / Favours comparator / Towards null /Away from null / Unpredictable

Supplementary file

Domain 2: Risk of bias due to deviations from the intended interventions (*effect of assignment to intervention*)

Signalling questions	Comments	Response options
**2.1. Were participants aware of their assigned intervention during the trial?**	Probably no. A psychological placebo (AT) was set as a control condition.	Y / PY / PN / N / NI
**2.2. Were carers and people delivering the interventions aware of participants' assigned intervention during the trial?**	Yes.	Y / PY / PN / N / NI
**2.3. If Y/PY/NI to 2.1 or 2.2: Were there deviations from the intended intervention that arose because of the trial context?**	Probably no.	NA / Y / PY / PN / N / NI
**2.4 If Y/PY to 2.3: Were these deviations likely to have affected the outcome?**	NA.	NA / Y / PY / PN / N / NI
**2.5. If Y/PY/NI to 2.4: Were these deviations from intended intervention balanced between groups?**	NA.	NA / Y / PY / PN / N / NI
**2.6 Was an appropriate analysis used to estimate the effect of assignment to intervention?**	No information.	Y / PY / PN / N / NI
**2.7 If N/PN/NI to 2.6: Was there potential for a substantial impact (on the result) of the failure to analyse participants in the group to which they were randomized?**	Probably yes. Although the reasons are disclosed, the intervention group has a higher dropout rate than the control group.	NA / Y / PY / PN / N / NI
**Risk-of-bias judgement**	High risk of bias.	Low / High / Some concerns
Optional: What is the predicted direction of bias due to deviations from intended interventions?	Unpredicatable.	NA / Favours experimental / Favours comparator / Towards null /Away from null / Unpredictable

Supplementary file

Domain 3: Missing outcome data

Signalling questions	Comments	Response options
**3.1 Were data for this outcome available for all, or nearly all, participants randomized?**	No.	Y / PY / PN / N / NI
**3.2 If N/PN/NI to 3.1: Is there evidence that the result was not biased by missing outcome data?**	Probably no.	NA / Y / PY / PN / N
**3.3 If N/PN to 3.2: Could missingness in the outcome depend on its true value?**	Probably no. This RCT confirms that there is no difference in the characteristics of participants who have completed treatment and those who have dropped out.	NA / Y / PY / PN / N / NI
**3.4 If Y/PY/NI to 3.3: Is it likely that missingness in the outcome depended on its true value?**	NA.	NA / Y / PY / PN / N / NI
**Risk-of-bias judgement**	Low risk of bias.	Low / High / Some concerns
Optional: What is the predicted direction of bias due to missing outcome data?	NA.	NA / Favours experimental / Favours comparator / Towards null /Away from null / Unpredictable

Supplementary file

Domain 4: Risk of bias in measurement of the outcome

Signalling questions	Comments	Response options
**4.1 Was the method of measuring the outcome inappropriate?**	No.	Y / PY / PN / N / NI
**4.2 Could measurement or ascertainment of the outcome have differed between intervention groups?**	No.	Y / PY / PN / N / NI
**4.3 If N/PN/NI to 4.1 and 4.2: Were outcome assessors aware of the intervention received by study participants?**	Probably yes.	NA / Y / PY / PN / N / NI
**4.4 If Y/PY/NI to 4.3: Could assessment of the outcome have been influenced by knowledge of intervention received?**	Probably no. GAD-7 is a self-administered generalized anxiety severity endpoint, and the evaluator's knowledge probably did not affect the results.	NA / Y / PY / PN / N / NI
**4.5 If Y/PY/NI to 4.4: Is it likely that assessment of the outcome was influenced by knowledge of intervention received?**	NA.	NA / Y / PY / PN / N / NI
**Risk-of-bias judgement**	Low risk of bias.	Low / High / Some concerns
Optional: What is the predicted direction of bias in measurement of the outcome?	NA.	NA / Favours experimental / Favours comparator / Towards null /Away from null / Unpredictable

Supplementary file

Domain 5: Risk of bias in selection of the reported result

Signalling questions	Comments	Response options
**5.1 Were the data that produced this result analysed in accordance with a pre-specified analysis plan that was finalized before unblinded outcome data were available for analysis?**	Yes. Pre-specified outcome was reported.	Y / PY / PN / N / NI
**Is the numerical result being assessed likely to have been selected, on the basis of the results, from...**		
**5.2. ... multiple eligible outcome measurements (e.g. scales, definitions, time points) within the outcome domain?**	No. Pre-specified outcome was reported.	Y / PY / PN / N / NI
**5.3 ... multiple eligible analyses of the data?**	No. Pre-specified outcome was reported.	Y / PY / PN / N / NI
**Risk-of-bias judgement**	Low risk of bias.	Low / High / Some concerns
Optional: What is the predicted direction of bias due to selection of the reported result?	NA.	NA / Favours experimental / Favours comparator / Towards null /Away from null / Unpredictable

Supplementary file

Overall risk of bias

**Risk-of-bias judgement**	High risk of bias.	Low / High / Some concerns
Optional: What is the overall predicted direction of bias for this outcome?	Unpredictable.	NA / Favours experimental / Favours comparator / Towards null /Away from null / Unpredictable

Supplementary file

Study details

**Reference**	Elliott TR, Brossart D, Berry JW, Fine PR. Problem-solving training via videoconferencing for family caregivers of persons with spinal cord injuries: a randomized controlled trial. Behav Res Ther. 2008;46(11):1220-1229.

Study design

X Individually-randomized parallel-group trial
☐ Cluster-randomized parallel-group trial
☐ Individually randomized cross-over (or other matched) trial

For the purposes of this assessment, the interventions being compared are defined as

Experimental: PST Comparator: Psychoeducation only

**Specify which outcome is being assessed for risk of bias**	Inventory to diagnose depression (IDD)
**Specify the numerical result being assessed.** In case of multiple alternative analyses being presented, specify the numeric result (e.g. RR = 1.52 (95% CI 0.83 to 2.77) and/or a reference (e.g. to a table, figure or paragraph) that uniquely defines the result being assessed.	At the 6 month evaluation, participants in the PST group reported less depression than the education group.

Is the review team's aim for this result…?

X to assess the effect of *assignment to intervention* (the 'intention-to-treat' effect)
☐ to assess the effect of *adhering to intervention* (the 'per-protocol' effect)

If the aim is to assess the effect of *adhering to intervention*, select the deviations from intended intervention that should be addressed (at least one must be checked):

☐ occurrence of non-protocol interventions
☐ failures in implementing the intervention that could have affected the outcome
☐ non-adherence to their assigned intervention by trial participants

Which of the following sources were <u>obtained</u> to help inform the risk-of-bias assessment? (tick as many as apply)

Supplementary file

X	Journal article(s) with results of the trial
☐	Trial protocol
☐	Statistical analysis plan (SAP)
☐	Non-commercial trial registry record (e.g. ClinicalTrials.gov record)
☐	Company-owned trial registry record (e.g. GSK Clinical Study Register record)
☐	"Grey literature" (e.g. unpublished thesis)
☐	Conference abstract(s) about the trial
☐	Regulatory document (e.g. Clinical Study Report, Drug Approval Package)
☐	Research ethics application
☐	Grant database summary (e.g. NIH RePORTER or Research Councils UK Gateway to Research)
☐	Personal communication with trialist
☐	Personal communication with the sponsor

Supplementary file

Risk of bias assessment

Responses underlined in green are potential markers for low risk of bias, and responses in red are potential markers for a risk of bias. Where questions relate only to sign posts to other questions, no formatting is used.

Domain 1: Risk of bias arising from the randomization process

Signalling questions	Comments	Response options
**1.1 Was the allocation sequence random?**	Yes. "The first author used a simple randomization strategy (with a random numbers table) to assign participants to the PST group or to the education-only control group."	Y / PY / PN / N / NI
**1.2 Was the allocation sequence concealed until participants were enrolled and assigned to interventions?**	Yes. "The first author had no information about the caregiver or care recipient at the time of randomization."	Y / PY / PN / N / NI
**1.3 Did baseline differences between intervention groups suggest a problem with the randomization process?**	No.	Y / PY / PN / N / NI
**Risk-of-bias judgement**	Low risk of bias.	Low / High / Some concerns
Optional: What is the predicted direction of bias arising from the randomization process?	NA.	NA / Favours experimental / Favours comparator / Towards null /Away from null / Unpredictable

Supplementary file

Domain 2: Risk of bias due to deviations from the intended interventions (*effect of assignment to intervention*)

Signalling questions	Comments	Response options
**2.1. Were participants aware of their assigned intervention during the trial?**	Probably no. The Psychological placebo (education only) is provided to the control group in this RCT.	Y / PY / PN / N / NI
**2.2. Were carers and people delivering the interventions aware of participants' assigned intervention during the trial?**	Probably yes.	Y / PY / PN / N / NI
**2.3. If Y/PY/NI to 2.1 or 2.2: Were there deviations from the intended intervention that arose because of the trial context?**	Probably no.	NA / Y / PY / PN / N / NI
**2.4 If Y/PY to 2.3: Were these deviations likely to have affected the outcome?**	NA.	NA / Y / PY / PN / N / NI
**2.5. If Y/PY/NI to 2.4: Were these deviations from intended intervention balanced between groups?**	NA.	NA / Y / PY / PN / N / NI
**2.6 Was an appropriate analysis used to estimate the effect of assignment to intervention?**	Yes. ITT analyses were conducted.	Y / PY / PN / N / NI
**2.7 If N/PN/NI to 2.6: Was there potential for a substantial impact (on the result) of the failure to analyse participants in the group to which they were randomized?**	NA.	NA / Y / PY / PN / N / NI
**Risk-of-bias judgement**	Low risk of bias.	Low / High / Some concerns
Optional: What is the predicted direction of bias due to deviations from intended interventions?	NA.	NA / Favours experimental / Favours comparator / Towards null /Away from null / Unpredictable

Supplementary file

Domain 3: Missing outcome data

Signalling questions	Comments	Response options
**3.1 Were data for this outcome available for all, or nearly all, participants randomized?**	No. 22 patients lost to follow-up. Reasons for losses were not disclosed. Losses likely to affect results.	Y / PY / PN / N / NI
**3.2 If N/PN/NI to 3.1: Is there evidence that the result was not biased by missing outcome data?**	Probably no.	NA / Y / PY / PN / N
**3.3 If N/PN to 3.2: Could missingness in the outcome depend on its true value?**	Probably yes.<br><br>Probably no.	NA / Y / PY / PN / N / NI
**3.4 If Y/PY/NI to 3.3: Is it likely that missingness in the outcome depended on its true value?**		NA / Y / PY / PN / N / NI
**Risk-of-bias judgement**	Some concerns.	Low / High / Some concerns
Optional: What is the predicted direction of bias due to missing outcome data?	Favours experimental.	NA / Favours experimental / Favours comparator / Towards null /Away from null / Unpredictable

Supplementary file

Domain 4: Risk of bias in measurement of the outcome

Signalling questions	Comments	Response options
**4.1 Was the method of measuring the outcome inappropriate?**	No.	Y / PY / PN / N / NI
**4.2 Could measurement or ascertainment of the outcome have differed between intervention groups?**	Probably no.	Y / PY / PN / N / NI
**4.3 If N/PN/NI to 4.1 and 4.2: Were outcome assessors aware of the intervention received by study participants?**	Probably no.	NA / Y / PY / PN / N / NI
**4.4 If Y/PY/NI to 4.3: Could assessment of the outcome have been influenced by knowledge of intervention received?**	NA.	NA / Y / PY / PN / N / NI
**4.5 If Y/PY/NI to 4.4: Is it likely that assessment of the outcome was influenced by knowledge of intervention received?**		NA / Y / PY / PN / N / NI
**Risk-of-bias judgement**	Low risk of bias.	Low / High / Some concerns
Optional: What is the predicted direction of bias in measurement of the outcome?	NA.	NA / Favours experimental / Favours comparator / Towards null /Away from null / Unpredictable

Supplementary file

Domain 5: Risk of bias in selection of the reported result

Signalling questions	Comments	Response options
**5.1 Were the data that produced this result analysed in accordance with a pre-specified analysis plan that was finalized before unblinded outcome data were available for analysis?**	No information.	Y / PY / PN / N / NI
**Is the numerical result being assessed likely to have been selected, on the basis of the results, from...**		
**5.2. ... multiple eligible outcome measurements (e.g. scales, definitions, time points) within the outcome domain?**	No. Depressive symptoms are evaluated on only one outcome (IDD).	Y / PY / PN / N / NI
**5.3 ... multiple eligible analyses of the data?**	No.	Y / PY / PN / N / NI
**Risk-of-bias judgement**	Some concern. The number of dropouts for each reported reason is similar between groups.	Low / High / Some concerns
Optional: What is the predicted direction of bias due to selection of the reported result?	NA.	NA / Favours experimental / Favours comparator / Towards null /Away from null / Unpredictable

Supplementary file

Overall risk of bias

**Risk-of-bias judgement**	Some concerns.	Low / High / Some concerns
Optional: What is the overall predicted direction of bias for this outcome?	Favours experimental.	NA / Favours experimental / Favours comparator / Towards null /Away from null / Unpredictable

Supplementary file

Study details

Reference

Ferguson RJ, Sigmon ST, Pritchard AJ, LaBrie SL, Goetze RE, Fink CM, Garrett AM. A randomized trial of videoconference-delivered cognitive behavioral therapy for survivors of breast cancer with self-reported cognitive dysfunction. Cancer 2016;122(11):1782-1791.

Study design

X Individually-randomized parallel-group trial
☐ Cluster-randomized parallel-group trial
☐ Individually randomized cross-over (or other matched) trial

For the purposes of this assessment, the interventions being compared are defined as

Experimental: VCBT Comparator: AT

**Specify which outcome is being assessed for risk of bias**	Depression on depression anxiety stress scales (DASS)
**Specify the numerical result being assessed.** In case of multiple alternative analyses being presented, specify the numeric result (e.g. RR = 1.52 (95% CI 0.83 to 2.77) and/or a reference (e.g. to a table, figure or paragraph) that uniquely defines the result being assessed.	The scores of DASS depression changed 12.62 (SD = 9.36), 6.00(SD=6.62) to 7.27 (SD = 7.66), 3.70 (SD = 4.27) in AT and VCBT group from baseline to after treatment.

Is the review team's aim for this result…?

X to assess the effect of *assignment to intervention* (the 'intention-to-treat' effect)
☐ to assess the effect of *adhering to intervention* (the 'per-protocol' effect)

If the aim is to assess the effect of *adhering to intervention*, select the deviations from intended intervention that should be addressed (at least one must be checked):

☐ occurrence of non-protocol interventions
☐ failures in implementing the intervention that could have affected the outcome
☐ non-adherence to their assigned intervention by trial participants

Which of the following sources were <u>obtained</u> to help inform the risk-of-bias assessment? (tick as many as apply)

Supplementary file

X	Journal article(s) with results of the trial
☐	Trial protocol
☐	Statistical analysis plan (SAP)
☐	Non-commercial trial registry record (e.g. ClinicalTrials.gov record)
☐	Company-owned trial registry record (e.g. GSK Clinical Study Register record)
☐	"Grey literature" (e.g. unpublished thesis)
☐	Conference abstract(s) about the trial
☐	Regulatory document (e.g. Clinical Study Report, Drug Approval Package)
☐	Research ethics application
☐	Grant database summary (e.g. NIH RePORTER or Research Councils UK Gateway to Research)
☐	Personal communication with trialist
☐	Personal communication with the sponsor

Supplementary file

Risk of bias assessment

Responses underlined in green are potential markers for low risk of bias, and responses in red are potential markers for a risk of bias. Where questions relate only to sign posts to other questions, no formatting is used.

Domain 1: Risk of bias arising from the randomization process

Signalling questions	Comments	Response options
**1.1 Was the allocation sequence random?**	No information.	Y / PY / PN / N / NI
**1.2 Was the allocation sequence concealed until participants were enrolled and assigned to interventions?**	No information.	Y / PY / PN / N / NI
**1.3 Did baseline differences between intervention groups suggest a problem with the randomization process?**	No.	Y / PY / PN / N / NI
**Risk-of-bias judgement**	Some concerns.	Low / High / Some concerns
Optional: What is the predicted direction of bias arising from the randomization process?	Unpredictable.	NA / Favours experimental / Favours comparator / Towards null /Away from null / Unpredictable

Supplementary file

Domain 2: Risk of bias due to deviations from the intended interventions (*effect of assignment to intervention*)

Signalling questions	Comments	Response options
**2.1. Were participants aware of their assigned intervention during the trial?**	Probably no. A psychological placebo was set as a control group in this RCT.	Y / PY / PN / N / NI
**2.2. Were carers and people delivering the interventions aware of participants' assigned intervention during the trial?**	Yes.	Y / PY / PN / N / NI
**2.3. If Y/PY/NI to 2.1 or 2.2: Were there deviations from the intended intervention that arose because of the trial context?**	No.	NA / Y / PY / PN / N / NI
**2.4 If Y/PY to 2.3: Were these deviations likely to have affected the outcome?**	NA.	NA / Y / PY / PN / N / NI
**2.5. If Y/PY/NI to 2.4: Were these deviations from intended intervention balanced between groups?**	NA.	NA / Y / PY / PN / N / NI
**2.6 Was an appropriate analysis used to estimate the effect of assignment to intervention?**	Probably yes. "The main hypotheses were tested with analysis of covariance with the baseline score as the covariate and testing separately for group differences at the posttreatment and 2-month follow-up time points."	Y / PY / PN / N / NI
**2.7 If N/PN/NI to 2.6: Was there potential for a substantial impact (on the result) of the failure to analyse participants in the group to which they were randomized?**	NA.	NA / Y / PY / PN / N / NI
**Risk-of-bias judgement**	Low risk of bias.	Low / High / Some concerns
Optional: What is the predicted direction of bias due to deviations from intended interventions?	NA.	NA / Favours experimental / Favours comparator / Towards null /Away from null / Unpredictable

Supplementary file

Domain 3: Missing outcome data

Signalling questions	Comments	Response options
**3.1 Were data for this outcome available for all, or nearly all, participants randomized?**	Probably no. In this RCT, there was a 25.5% (n = 12/47) dropout.	Y / PY / PN / N / NI
**3.2 If N/PN/NI to 3.1: Is there evidence that the result was not biased by missing outcome data?**	No.	NA / Y / PY / PN / N
**3.3 If N/PN to 3.2: Could missingness in the outcome depend on its true value?**	Probably yes.<br><br>Probably no.	NA / Y / PY / PN / N / NI
**3.4 If Y/PY/NI to 3.3: Is it likely that missingness in the outcome depended on its true value?**		NA / Y / PY / PN / N / NI
**Risk-of-bias judgement**	Some concerns.	Low / High / Some concerns
Optional: What is the predicted direction of bias due to missing outcome data?	Favours experimental.	NA / Favours experimental / Favours comparator / Towards null /Away from null / Unpredictable

Supplementary file

Domain 4: Risk of bias in measurement of the outcome

Signalling questions	Comments	Response options
**4.1 Was the method of measuring the outcome inappropriate?**	No.	Y / PY / PN / N / NI
**4.2 Could measurement or ascertainment of the outcome have differed between intervention groups?**	No.	Y / PY / PN / N / NI
**4.3 If N/PN/NI to 4.1 and 4.2: Were outcome assessors aware of the intervention received by study participants?**	Probably no. "The psychometrist responsible for all assessments remained blind to each participant's assigned treatment condition throughout the study."	NA / Y / PY / PN / N / NI
**4.4 If Y/PY/NI to 4.3: Could assessment of the outcome have been influenced by knowledge of intervention received?**	NA.	NA / Y / PY / PN / N / NI
**4.5 If Y/PY/NI to 4.4: Is it likely that assessment of the outcome was influenced by knowledge of intervention received?**		NA / Y / PY / PN / N / NI
**Risk-of-bias judgement**	Low risk of bias.	Low / High / Some concerns
Optional: What is the predicted direction of bias in measurement of the outcome?	NA.	NA / Favours experimental / Favours comparator / Towards null /Away from null / Unpredictable

Supplementary file

Domain 5: Risk of bias in selection of the reported result

Signalling questions	Comments	Response options
**5.1 Were the data that produced this result analysed in accordance with a pre-specified analysis plan that was finalized before unblinded outcome data were available for analysis?**	No information.	Y / PY / PN / N / NI
**Is the numerical result being assessed likely to have been selected, on the basis of the results, from...**		
**5.2. ... multiple eligible outcome measurements (e.g. scales, definitions, time points) within the outcome domain?**	Probably no. There was only one outcome to assess depressive symptoms.	Y / PY / PN / N / NI
**5.3 ... multiple eligible analyses of the data?**	No. There is only one possible way in which the outcome measurement can be analysed (hence there is no opportunity to select from multiple analyses).	Y / PY / PN / N / NI
**Risk-of-bias judgement**	Some concerns.	Low / High / Some concerns
Optional: What is the predicted direction of bias due to selection of the reported result?	NA.	NA / Favours experimental / Favours comparator / Towards null /Away from null / Unpredictable

Supplementary file

Overall risk of bias

**Risk-of-bias judgement**	Some concerns.	Low / High / Some concerns
Optional: What is the overall predicted direction of bias for this outcome?	Favours experimental.	NA / Favours experimental / Favours comparator / Towards null /Away from null / Unpredictable

Supplementary file

Study details

**Reference**	El-Jawahri A, Jacobs JM, Nelson AM, Traeger L, Greer JA, Nicholson S, et al. Multimodal psychosocial intervention for family caregivers of patients undergoing hematopoietic stem cell transplantation: A randomized clinical trial. Cancer. 2020;126(8):1758-1765.

Study design

X Individually-randomized parallel-group trial
☐ Cluster-randomized parallel-group trial
☐ Individually randomized cross-over (or other matched) trial

For the purposes of this assessment, the interventions being compared are defined as

Experimental: VCBT Comparator: Treatment as usual (TAU)

**Specify which outcome is being assessed for risk of bias**	Hospital anxiety and depression scale (HASD)
**Specify the numerical result being assessed.** In case of multiple alternative analyses being presented, specify the numeric result (e.g. RR = 1.52 (95% CI 0.83 to 2.77) and/or a reference (e.g. to a table, figure or paragraph) that uniquely defines the result being assessed.	Participants randomized to VCBT reported improved depression symptoms (B = -1.23; 95% CI, -1.92 to -0.54; P < .001), in comparison with the TAU group.

Is the review team's aim for this result…?

X to assess the effect of *assignment to intervention* (the 'intention-to-treat' effect)
☐ to assess the effect of *adhering to intervention* (the 'per-protocol' effect)

If the aim is to assess the effect of *adhering to intervention*, select the deviations from intended intervention that should be addressed (at least one must be checked):

☐ occurrence of non-protocol interventions
☐ failures in implementing the intervention that could have affected the outcome
☐ non-adherence to their assigned intervention by trial participants

Which of the following sources were <u>obtained</u> to help inform the risk-of-bias assessment? (tick as many as apply)

Supplementary file

X	Journal article(s) with results of the trial
☐	Trial protocol
☐	Statistical analysis plan (SAP)
X	Non-commercial trial registry record (e.g. ClinicalTrials.gov record)
☐	Company-owned trial registry record (e.g. GSK Clinical Study Register record)
☐	“Grey literature” (e.g. unpublished thesis)
☐	Conference abstract(s) about the trial
☐	Regulatory document (e.g. Clinical Study Report, Drug Approval Package)
☐	Research ethics application
☐	Grant database summary (e.g. NIH RePORTER or Research Councils UK Gateway to Research)
☐	Personal communication with trialist
☐	Personal communication with the sponsor

Supplementary file

Risk of bias assessment

Responses underlined in green are potential markers for low risk of bias, and responses in red are potential markers for a risk of bias. Where questions relate only to sign posts to other questions, no formatting is used.

Domain 1: Risk of bias arising from the randomization process

Signalling questions	Comments	Response options
**1.1 Was the allocation sequence random?**	Yes. "Participants, stratified by the type of transplant (autologous or allogeneic), were randomized in a 1:1 fashion by the Office of Data Quality with a computer-generated number sequence, which was concealed until after the group assignment."	Y / PY / PN / N / NI
**1.2 Was the allocation sequence concealed until participants were enrolled and assigned to interventions?**	Yes. "The Dana Farber/Harvard Cancer Center Office of Data Quality was responsible for participants' registration and assignment to the study groups, but it was not involved in other study procedures."	Y / PY / PN / N / NI
**1.3 Did baseline differences between intervention groups suggest a problem with the randomization process?**	No.	Y / PY / PN / N / NI
**Risk-of-bias judgement**	Low risk of bias.	Low / High / Some concerns
Optional: What is the predicted direction of bias arising from the randomization process?	NA.	NA / Favours experimental / Favours comparator / Towards null /Away from null / Unpredictable

Supplementary file

Domain 2: Risk of bias due to deviations from the intended interventions (*effect of assignment to intervention*)

Signalling questions	Comments	Response options
**2.1. Were participants aware of their assigned intervention during the trial?**	Probably yes.	Y / PY / PN / N / NI
**2.2. Were carers and people delivering the interventions aware of participants' assigned intervention during the trial?**	Yes.	Y / PY / PN / N / NI
**2.3. If Y/PY/NI to 2.1 or 2.2: Were there deviations from the intended intervention that arose because of the trial context?**	Probably No.	NA / Y / PY / PN / N / NI
**2.4 If Y/PY to 2.3: Were these deviations likely to have affected the outcome?**	NA.	NA / Y / PY / PN / N / NI
**2.5. If Y/PY/NI to 2.4: Were these deviations from intended intervention balanced between groups?**	NA.	NA / Y / PY / PN / N / NI
**2.6 Was an appropriate analysis used to estimate the effect of assignment to intervention?**	Probably no. "BMT-CARE (VCBT) was deemed feasible if at least 60% of eligible caregivers enrolled in the study and 60% of those assigned to the intervention completed a minimum of 50% of the planned intervention sessions."	Y / PY / PN / N / NI
**2.7 If N/PN/NI to 2.6: Was there potential for a substantial impact (on the result) of the failure to analyse participants in the group to which they were randomized?**	Probably no. This RCT excluded participants who have not achieved 50% of the intervention from the analysis. However, since very few data were excluded (5.4%; n = 5/92), it seems to have little impact on the results.	NA / Y / PY / PN / N / NI
**Risk-of-bias judgement**	Some concerns.	Low / High / Some concerns
Optional: What is the predicted direction of bias due to deviations from intended interventions?	Towards null.	NA / Favours experimental / Favours comparator / Towards null /Away from null / Unpredictable

Supplementary file

Domain 3: Missing outcome data

Signalling questions	Comments	Response options
**3.1 Were data for this outcome available for all, or nearly all, participants randomized?**	Yes. About 95% of the data has been analyzed.	Y / PY / PN / N / NI
**3.2 If N/PN/NI to 3.1: Is there evidence that the result was not biased by missing outcome data?**	NA.	NA / Y / PY / PN / N
**3.3 If N/PN to 3.2: Could missingness in the outcome depend on its true value?**	NA.	NA / Y / PY / PN / N / NI
**3.4 If Y/PY/NI to 3.3: Is it likely that missingness in the outcome depended on its true value?**	NA.	NA / Y / PY / PN / N / NI
**Risk-of-bias judgement**	Low risk of bias.	Low / High / Some concerns
Optional: What is the predicted direction of bias due to missing outcome data?	NA.	NA / Favours experimental / Favours comparator / Towards null /Away from null / Unpredictable

Supplementary file

Domain 4: Risk of bias in measurement of the outcome

Signalling questions	Comments	Response options
**4.1 Was the method of measuring the outcome inappropriate?**	Yes.	Y / PY / PN / N / NI
**4.2 Could measurement or ascertainment of the outcome have differed between intervention groups?**	No.	Y / PY / PN / N / NI
**4.3 If N/PN/NI to 4.1 and 4.2: Were outcome assessors aware of the intervention received by study participants?**	Yes.	NA / Y / PY / PN / N / NI
**4.4 If Y/PY/NI to 4.3: Could assessment of the outcome have been influenced by knowledge of intervention received?**	Probably no. Since HADS is a proven self-assessment measure of reliability and validity, it is unlikely that the knowledge of the intervention will affect outcomes.	NA / Y / PY / PN / N / NI
**4.5 If Y/PY/NI to 4.4: Is it likely that assessment of the outcome was influenced by knowledge of intervention received?**		NA / Y / PY / PN / N / NI
**Risk-of-bias judgement**	Low risk of bias.	Low / High / Some concerns
Optional: What is the predicted direction of bias in measurement of the outcome?	NA.	NA / Favours experimental / Favours comparator / Towards null /Away from null / Unpredictable

Supplementary file

Domain 5: Risk of bias in selection of the reported result

Signalling questions	Comments	Response options
**5.1 Were the data that produced this result analysed in accordance with a pre-specified analysis plan that was finalized before unblinded outcome data were available for analysis?**	Yes. ClinicalTrials.gov identifier NCT03328663.	Y / PY / PN / N / NI
**Is the numerical result being assessed likely to have been selected, on the basis of the results, from...**		
**5.2. ... multiple eligible outcome measurements (e.g. scales, definitions, time points) within the outcome domain?**	No.	Y / PY / PN / N / NI
**5.3 ... multiple eligible analyses of the data?**	No.	Y / PY / PN / N / NI
**Risk-of-bias judgement**	Low risk of bias.	Low / High / Some concerns
Optional: What is the predicted direction of bias due to selection of the reported result?	NA.	NA / Favours experimental / Favours comparator / Towards null /Away from null / Unpredictable

Supplementary file

Overall risk of bias

**Risk-of-bias judgement**	Some concerns.	Low / High / Some concerns
Optional: What is the overall predicted direction of bias for this outcome?	NA.	NA / Favours experimental / Favours comparator / Towards null /Away from null / Unpredictable

Supplementary file

Study details

**Reference**	Fox RS, Moreno PI, Yanez B, Estabrook R, Thomas J, Bouchard LC, et al. Integrating PROMIS® computerized adaptive tests into a web-based intervention for prostate cancer. Health Psychol. 2019;38(5):403-409.

Study design

X Individually-randomized parallel-group trial
☐ Cluster-randomized parallel-group trial
☐ Individually randomized cross-over (or other matched) trial

For the purposes of this assessment, the interventions being compared are defined as

Experimental: VCBT Comparator: Attention control (AC)

**Specify which outcome is being assessed for risk of bias**	Depression of PROMIS CAT scores
**Specify the numerical result being assessed.** In case of multiple alternative analyses being presented, specify the numeric result (e.g. RR = 1.52 (95% CI 0.83 to 2.77) and/or a reference (e.g. to a table, figure or paragraph) that uniquely defines the result being assessed.	Multilevel modeling showed no statistically significant differences by group regarding change in psychosocial functioning from week 1 to week 10.

Is the review team's aim for this result…?

X to assess the effect of *assignment to intervention* (the 'intention-to-treat' effect)
☐ to assess the effect of *adhering to intervention* (the 'per-protocol' effect)

If the aim is to assess the effect of *adhering to intervention*, select the deviations from intended intervention that should be addressed (at least one must be checked):

☐ occurrence of non-protocol interventions
☐ failures in implementing the intervention that could have affected the outcome
☐ non-adherence to their assigned intervention by trial participants

Which of the following sources were <u>obtained</u> to help inform the risk-of-bias assessment? (tick as many as apply)

Supplementary file

X	Journal article(s) with results of the trial
☐	Trial protocol
X	Statistical analysis plan (SAP)
☐	Non-commercial trial registry record (e.g. ClinicalTrials.gov record)
☐	Company-owned trial registry record (e.g. GSK Clinical Study Register record)
☐	“Grey literature” (e.g. unpublished thesis)
☐	Conference abstract(s) about the trial
☐	Regulatory document (e.g. Clinical Study Report, Drug Approval Package)
☐	Research ethics application
☐	Grant database summary (e.g. NIH RePORTER or Research Councils UK Gateway to Research)
☐	Personal communication with trialist
☐	Personal communication with the sponsor

Supplementary file

Risk of bias assessment

Responses underlined in green are potential markers for low risk of bias, and responses in red are potential markers for a risk of bias. Where questions relate only to sign posts to other questions, no formatting is used.

Domain 1: Risk of bias arising from the randomization process

Signalling questions	Comments	Response options
**1.1 Was the allocation sequence random?**	Yes. "Groups were stratified by metastatic status, with men who had bone metastases assigned to groups separate from those with no metastases or metastases only to lymph nodes."	Y / PY / PN / N / NI
**1.2 Was the allocation sequence concealed until participants were enrolled and assigned to interventions?**	No information.	Y / PY / PN / N / NI
**1.3 Did baseline differences between intervention groups suggest a problem with the randomization process?**	No.	Y / PY / PN / N / NI
**Risk-of-bias judgement**	Some concerns.	Low / High / Some concerns
Optional: What is the predicted direction of bias arising from the randomization process?	Away from null.	NA / Favours experimental / Favours comparator / Towards null /Away from null / Unpredictable

Supplementary file

Domain 2: Risk of bias due to deviations from the intended interventions (*effect of assignment to intervention*)

Signalling questions	Comments	Response options
**2.1. Were participants aware of their assigned intervention during the trial?**	Probably no.	Y / PY / PN / N / NI
**2.2. Were carers and people delivering the interventions aware of participants' assigned intervention during the trial?**	Yes.	Y / PY / PN / N / NI
**2.3. If Y/PY/NI to 2.1 or 2.2: Were there deviations from the intended intervention that arose because of the trial context?**	No.	NA / Y / PY / PN / N / NI
**2.4 If Y/PY to 2.3: Were these deviations likely to have affected the outcome?**	NA.	NA / Y / PY / PN / N / NI
**2.5. If Y/PY/NI to 2.4: Were these deviations from intended intervention balanced between groups?**	NA.	NA / Y / PY / PN / N / NI
**2.6 Was an appropriate analysis used to estimate the effect of assignment to intervention?**	Yes. All analyses were completed as intent-to-treat analyses.	Y / PY / PN / N / NI
**2.7 If N/PN/NI to 2.6: Was there potential for a substantial impact (on the result) of the failure to analyse participants in the group to which they were randomized?**	NA.	NA / Y / PY / PN / N / NI
**Risk-of-bias judgement**	Low risk of bias.	Low / High / Some concerns
Optional: What is the predicted direction of bias due to deviations from intended interventions?	NA.	NA / Favours experimental / Favours comparator / Towards null /Away from null / Unpredictable

Supplementary file

Domain 3: Missing outcome data

Signalling questions	Comments	Response options
**3.1 Were data for this outcome available for all, or nearly all, participants randomized?**	No. 43 patients lost to follow-up, reasons for losses were disclosed. Losses likely to influence final results.	Y / PY / PN / N / NI
**3.2 If N/PN/NI to 3.1: Is there evidence that the result was not biased by missing outcome data?**	No.	NA / Y / PY / PN / N
**3.3 If N/PN to 3.2: Could missingness in the outcome depend on its true value?**	Probably yes.<br><br>Probably no.	NA / Y / PY / PN / N / NI
**3.4 If Y/PY/NI to 3.3: Is it likely that missingness in the outcome depended on its true value?**		NA / Y / PY / PN / N / NI
**Risk-of-bias judgement**	Some concerns.	Low / High / Some concerns
Optional: What is the predicted direction of bias due to missing outcome data?	Unpredictable.	NA / Favours experimental / Favours comparator / Towards null /Away from null / Unpredictable

Supplementary file

Domain 4: Risk of bias in measurement of the outcome

Signalling questions	Comments	Response options
**4.1 Was the method of measuring the outcome inappropriate?**	No.	Y / PY / PN / N / NI
**4.2 Could measurement or ascertainment of the outcome have differed between intervention groups?**	No.	Y / PY / PN / N / NI
**4.3 If N/PN/NI to 4.1 and 4.2: Were outcome assessors aware of the intervention received by study participants?**	No information.	NA / Y / PY / PN / N / NI
**4.4 If Y/PY/NI to 4.3: Could assessment of the outcome have been influenced by knowledge of intervention received?**	Probably no. The Depression of PROMIS CAT is a reliable and valid endpoint and probably does not affect the results.	NA / Y / PY / PN / N / NI
**4.5 If Y/PY/NI to 4.4: Is it likely that assessment of the outcome was influenced by knowledge of intervention received?**		NA / Y / PY / PN / N / NI
**Risk-of-bias judgement**	Low risk of bias.	Low / High / Some concerns
Optional: What is the predicted direction of bias in measurement of the outcome?	NA.	NA / Favours experimental / Favours comparator / Towards null /Away from null / Unpredictable

Supplementary file

Domain 5: Risk of bias in selection of the reported result

Signalling questions	Comments	Response options
**5.1 Were the data that produced this result analysed in accordance with a pre-specified analysis plan that was finalized before unblinded outcome data were available for analysis?**	Yes. ClinicalTrials.gov Identifier: NCT03149185.	Y / PY / PN / N / NI
**Is the numerical result being assessed likely to have been selected, on the basis of the results, from...**		
**5.2. ... multiple eligible outcome measurements (e.g. scales, definitions, time points) within the outcome domain?**	No.	Y / PY / PN / N / NI
**5.3 ... multiple eligible analyses of the data?**	No.	Y / PY / PN / N / NI
**Risk-of-bias judgement**	Low risk of bias.	Low / High / Some concerns
Optional: What is the predicted direction of bias due to selection of the reported result?	NA.	NA / Favours experimental / Favours comparator / Towards null /Away from null / Unpredictable

Overall risk of bias

**Risk-of-bias judgement**	Some concerns.	Low / High / Some concerns
Optional: What is the overall predicted direction of bias for this outcome?	NA.	NA / Favours experimental / Favours comparator / Towards null /Away from null / Unpredictable

Supplementary file

Study details

**Reference**	El-Morr C, Ritvo P, Ahmad F, Moineddin R; MVC Team. Effectiveness of an 8-Week Web-Based Mindfulness Virtual Community Intervention for University Students on Symptoms of Stress, Anxiety, and Depression: Randomized Controlled Trial [published correction appears in JMIR Ment Health. 2020 Sep 30;7(9):e24131]

Study design

X Individually-randomized parallel-group trial
☐ Cluster-randomized parallel-group trial
☐ Individually randomized cross-over (or other matched) trial

For the purposes of this assessment, the interventions being compared are defined as

Experimental: Tel mindfulness   Comparator: Waiting list control (WLC)

**Specify which outcome is being assessed for risk of bias**	PHQ-9
**Specify the numerical result being assessed.** In case of multiple alternative analyses being presented, specify the numeric result (e.g. RR = 1.52 (95% CI 0.83 to 2.77) and/or a reference (e.g. to a table, figure or paragraph) that uniquely defines the result being assessed.	At postintervention follow-up, according to the adjusted comparisons, there were statistically significant between-group reductions in depression scores (β=–2.21, P=.01) , compared with the WLC group.

Is the review team's aim for this result…?

X to assess the effect of *assignment to intervention* (the 'intention-to-treat' effect)
☐ to assess the effect of *adhering to intervention* (the 'per-protocol' effect)

If the aim is to assess the effect of *adhering to intervention*, select the deviations from intended intervention that should be addressed (at least one must be checked):

☐ occurrence of non-protocol interventions
☐ failures in implementing the intervention that could have affected the outcome
☐ non-adherence to their assigned intervention by trial participants

Which of the following sources were <u>obtained</u> to help inform the risk-of-bias assessment? (tick as many as apply)

Supplementary file

X	Journal article(s) with results of the trial
☐	Trial protocol
☐	Statistical analysis plan (SAP)
X	Non-commercial trial registry record (e.g. ClinicalTrials.gov record)
☐	Company-owned trial registry record (e.g. GSK Clinical Study Register record)
☐	“Grey literature” (e.g. unpublished thesis)
☐	Conference abstract(s) about the trial
☐	Regulatory document (e.g. Clinical Study Report, Drug Approval Package)
☐	Research ethics application
☐	Grant database summary (e.g. NIH RePORTER or Research Councils UK Gateway to Research)
☐	Personal communication with trialist
☐	Personal communication with the sponsor

Supplementary file

Risk of bias assessment

Responses underlined in green are potential markers for low risk of bias, and responses in red are potential markers for a risk of bias. Where questions relate only to sign posts to other questions, no formatting is used.

Domain 1: Risk of bias arising from the randomization process

Signalling questions	Comments	Response options
**1.1 Was the allocation sequence random?**	Yes. "Participating students were randomized to the MVC intervention or the WLC using 1:1 block randomization."	Y / PY / PN / N / NI
**1.2 Was the allocation sequence concealed until participants were enrolled and assigned to interventions?**	Yes. "Allocations were concealed in sequentially numbered opaque envelopes."	Y / PY / PN / N / NI
**1.3 Did baseline differences between intervention groups suggest a problem with the randomization process?**	No.	Y / PY / PN / N / NI
**Risk-of-bias judgement**	Low risk of bias.	Low / High / Some concerns
Optional: What is the predicted direction of bias arising from the randomization process?	NA.	NA / Favours experimental / Favours comparator / Towards null /Away from null / Unpredictable

Supplementary file

Domain 2: Risk of bias due to deviations from the intended interventions (*effect of assignment to intervention*)

Signalling questions	Comments	Response options
**2.1. Were participants aware of their assigned intervention during the trial?**	Yes.	Y / PY / PN / N / NI
**2.2. Were carers and people delivering the interventions aware of participants' assigned intervention during the trial?**	Yes.	Y / PY / PN / N / NI
**2.3. If Y/PY/NI to 2.1 or 2.2: Were there deviations from the intended intervention that arose because of the trial context?**	Probably no.	NA / Y / PY / PN / N / NI
**2.4 If Y/PY to 2.3: Were these deviations likely to have affected the outcome?**	NA.	NA / Y / PY / PN / N / NI
**2.5. If Y/PY/NI to 2.4: Were these deviations from intended intervention balanced between groups?**	NA.	NA / Y / PY / PN / N / NI
**2.6 Was an appropriate analysis used to estimate the effect of assignment to intervention?**	Yes. ITT analysis was conducted in this RCT.	Y / PY / PN / N / NI
**2.7 If N/PN/NI to 2.6: Was there potential for a substantial impact (on the result) of the failure to analyse participants in the group to which they were randomized?**	NA.	NA / Y / PY / PN / N / NI
**Risk-of-bias judgement**	Low risk of bias.	Low / High / Some concerns
Optional: What is the predicted direction of bias due to deviations from intended interventions?	NA.	NA / Favours experimental / Favours comparator / Towards null /Away from null / Unpredictable

Supplementary file

Domain 3: Missing outcome data

Signalling questions	Comments	Response options
**3.1 Were data for this outcome available for all, or nearly all, participants randomized?**	Probably yes. 92.5% (n = 148/160) of participants fully responsed the outcome.	Y / PY / PN / N / NI
**3.2 If N/PN/NI to 3.1: Is there evidence that the result was not biased by missing outcome data?**	NA.	NA / Y / PY / PN / N
**3.3 If N/PN to 3.2: Could missingness in the outcome depend on its true value?**	NA.	NA / Y / PY / PN / N / NI
**3.4 If Y/PY/NI to 3.3: Is it likely that missingness in the outcome depended on its true value?**		NA / Y / PY / PN / N / NI
**Risk-of-bias judgement**	Low risk of bias.	Low / High / Some concerns
Optional: What is the predicted direction of bias due to missing outcome data?	NA.	NA / Favours experimental / Favours comparator / Towards null /Away from null / Unpredictable

Supplementary file

Domain 4: Risk of bias in measurement of the outcome

Signalling questions	Comments	Response options
**4.1 Was the method of measuring the outcome inappropriate?**	No.	Y / PY / PN / N / NI
**4.2 Could measurement or ascertainment of the outcome have differed between intervention groups?**	No.	Y / PY / PN / N / NI
**4.3 If N/PN/NI to 4.1 and 4.2: Were outcome assessors aware of the intervention received by study participants?**	No.	NA / Y / PY / PN / N / NI
**4.4 If Y/PY/NI to 4.3: Could assessment of the outcome have been influenced by knowledge of intervention received?**	NA.	NA / Y / PY / PN / N / NI
**4.5 If Y/PY/NI to 4.4: Is it likely that assessment of the outcome was influenced by knowledge of intervention received?**		NA / Y / PY / PN / N / NI
**Risk-of-bias judgement**	Low risk of bias.	Low / High / Some concerns
Optional: What is the predicted direction of bias in measurement of the outcome?	NA.	NA / Favours experimental / Favours comparator / Towards null /Away from null / Unpredictable

Supplementary file

Domain 5: Risk of bias in selection of the reported result

Signalling questions	Comments	Response options
**5.1 Were the data that produced this result analysed in accordance with a pre-specified analysis plan that was finalized before unblinded outcome data were available for analysis?**	Yes. Trial Registration: ISRCTN Registry ISRCTN12249616; http://www.isrctn.com/ISRCTN12249616	Y / PY / PN / N / NI
**Is the numerical result being assessed likely to have been selected, on the basis of the results, from...**		
**5.2. ... multiple eligible outcome measurements (e.g. scales, definitions, time points) within the outcome domain?**	No.	Y / PY / PN / N / NI
**5.3 ... multiple eligible analyses of the data?**	No.	Y / PY / PN / N / NI
**Risk-of-bias judgement**	Low risk of bias.	Low / High / Some concerns
Optional: What is the predicted direction of bias due to selection of the reported result?	NA.	NA / Favours experimental / Favours comparator / Towards null /Away from null / Unpredictable

Supplementary file

Overall risk of bias

**Risk-of-bias judgement**	Low risk of bias.	Low / High / Some concerns
Optional: What is the overall predicted direction of bias for this outcome?	NA.	NA / Favours experimental / Favours comparator / Towards null /Away from null / Unpredictable

Supplementary file

Study details

Reference Morriss R, Patel S, Malins S, Guo B, Higton F, James M, et al. Clinical and economic outcomes of remotely delivered cognitive behaviour therapy versus treatment as usual for repeat unscheduled care users with severe health anxiety: a multicentre randomised controlled trial. BMC Med. 2019;17(1):16

Study design

X Individually-randomized parallel-group trial

☐ Cluster-randomized parallel-group trial

☐ Individually randomized cross-over (or other matched) trial

For the purposes of this assessment, the interventions being compared are defined as

Experimental: VCBT Comparator: TAU

**Specify which outcome is being assessed for risk of bias**	the 14-item Short Health Anxiety Inventory, SHAI
**Specify the numerical result being assessed.** In case of multiple alternative analyses being presented, specify the numeric result (e.g. RR = 1.52 (95% CI 0.83 to 2.77) and/or a reference (e.g. to a table, figure or paragraph) that uniquely defines the result being assessed.	Compared to TAU, RCBT significantly reduced health anxiety at six months, maintained to 9 and 12 months (mean change difference HAI –2.81; 95% CI –5.11 to –0.50; *P* = 0.017).

Is the review team's aim for this result…?

X to assess the effect of *assignment to intervention* (the 'intention-to-treat' effect)

☐ to assess the effect of *adhering to intervention* (the 'per-protocol' effect)

If the aim is to assess the effect of *adhering to intervention*, select the deviations from intended intervention that should be addressed (at least one must be checked):

☐ occurrence of non-protocol interventions

☐ failures in implementing the intervention that could have affected the outcome

☐ non-adherence to their assigned intervention by trial participants

Which of the following sources were <u>obtained</u> to help inform the risk-of-bias assessment? (tick as many as apply)

Supplementary file

X	Journal article(s) with results of the trial
☐	Trial protocol
☐	Statistical analysis plan (SAP)
X	Non-commercial trial registry record (e.g. ClinicalTrials.gov record)
☐	Company-owned trial registry record (e.g. GSK Clinical Study Register record)
☐	“Grey literature” (e.g. unpublished thesis)
☐	Conference abstract(s) about the trial
☐	Regulatory document (e.g. Clinical Study Report, Drug Approval Package)
☐	Research ethics application
☐	Grant database summary (e.g. NIH RePORTER or Research Councils UK Gateway to Research)
☐	Personal communication with trialist
☐	Personal communication with the sponsor

Supplementary file

Risk of bias assessment

Responses underlined in green are potential markers for low risk of bias, and responses in red are potential markers for a risk of bias. Where questions relate only to sign posts to other questions, no formatting is used.

Domain 1: Risk of bias arising from the randomization process

Signalling questions	Comments	Response options
**1.1 Was the allocation sequence random?**	Yes. "Randomisation was determined by a computer-generated pseudo-random code using random permuted blocks."	Y / PY / PN / N / NI
**1.2 Was the allocation sequence concealed until participants were enrolled and assigned to interventions?**	Yes. "Only the trial manager and the administration support officer had password access to the un-blinded randomisation data."	Y / PY / PN / N / NI
**1.3 Did baseline differences between intervention groups suggest a problem with the randomization process?**	No.	Y / PY / PN / N / NI
**Risk-of-bias judgement**	Low risk of bias.	Low / High / Some concerns
Optional: What is the predicted direction of bias arising from the randomization process?	NA.	NA / Favours experimental / Favours comparator / Towards null /Away from null / Unpredictable

Supplementary file

Domain 2: Risk of bias due to deviations from the intended interventions (*effect of assignment to intervention*)

Signalling questions	Comments	Response options
**2.1. Were participants aware of their assigned intervention during the trial?**	Yes.	Y / PY / PN / N / NI
**2.2. Were carers and people delivering the interventions aware of participants' assigned intervention during the trial?**	Yes.	Y / PY / PN / N / NI
**2.3. If Y/PY/NI to 2.1 or 2.2: Were there deviations from the intended intervention that arose because of the trial context?**	Probably no.	NA / Y / PY / PN / N / NI
**2.4 If Y/PY to 2.3: Were these deviations likely to have affected the outcome?**	NA.	NA / Y / PY / PN / N / NI
**2.5. If Y/PY/NI to 2.4: Were these deviations from intended intervention balanced between groups?**	NA.	NA / Y / PY / PN / N / NI
**2.6 Was an appropriate analysis used to estimate the effect of assignment to intervention?**	Yes. ITT analysis was conducted.	Y / PY / PN / N / NI
**2.7 If N/PN/NI to 2.6: Was there potential for a substantial impact (on the result) of the failure to analyse participants in the group to which they were randomized?**	NA.	NA / Y / PY / PN / N / NI
**Risk-of-bias judgement**	Low risk of bias.	Low / High / Some concerns
Optional: What is the predicted direction of bias due to deviations from intended interventions?	NA.	NA / Favours experimental / Favours comparator / Towards null /Away from null / Unpredictable

Supplementary file

Domain 3: Missing outcome data

Signalling questions	Comments	Response options
**3.1 Were data for this outcome available for all, or nearly all, participants randomized?**	No. 55 patients lost to follow-up, reasons for losses were disclosed.	Y / PY / PN / N / NI
**3.2 If N/PN/NI to 3.1: Is there evidence that the result was not biased by missing outcome data?**	No.	NA / Y / PY / PN / N
**3.3 If N/PN to 3.2: Could missingness in the outcome depend on its true value?**	No information.<br><br>No.	NA / Y / PY / PN / N / NI
**3.4 If Y/PY/NI to 3.3: Is it likely that missingness in the outcome depended on its true value?**		NA / Y / PY / PN / N / NI
**Risk-of-bias judgement**	Some concerns.	Low / High / Some concerns
Optional: What is the predicted direction of bias due to missing outcome data?	NA.	NA / Favours experimental / Favours comparator / Towards null /Away from null / Unpredictable

Supplementary file

Domain 4: Risk of bias in measurement of the outcome

Signalling questions	Comments	Response options
**4.1 Was the method of measuring the outcome inappropriate?**	No.	Y / PY / PN / N / NI
**4.2 Could measurement or ascertainment of the outcome have differed between intervention groups?**	No.	Y / PY / PN / N / NI
**4.3 If N/PN/NI to 4.1 and 4.2: Were outcome assessors aware of the intervention received by study participants?**	Yes.	NA / Y / PY / PN / N / NI
**4.4 If Y/PY/NI to 4.3: Could assessment of the outcome have been influenced by knowledge of intervention received?**	Probably no. Since SHAI is a proven self-assessment measure of reliability and validity, knowledge of interventions is unlikely to affect outcomes.	NA / Y / PY / PN / N / NI
**4.5 If Y/PY/NI to 4.4: Is it likely that assessment of the outcome was influenced by knowledge of intervention received?**	NA.	NA / Y / PY / PN / N / NI
**Risk-of-bias judgement**	Low risk of bias.	Low / High / Some concerns
Optional: What is the predicted direction of bias in measurement of the outcome?	NA.	NA / Favours experimental / Favours comparator / Towards null /Away from null / Unpredictable

Supplementary file

Domain 5: Risk of bias in selection of the reported result

Signalling questions	Comments	Response options
**5.1 Were the data that produced this result analysed in accordance with a pre-specified analysis plan that was finalized before unblinded outcome data were available for analysis?**	Yes. The trial was registered at ClinicalTrials.gov on 19 Nov 2014 with reference number NCT02298036.	Y / PY / PN / N / NI
**Is the numerical result being assessed likely to have been selected, on the basis of the results, from...**		
**5.2. ... multiple eligible outcome measurements (e.g. scales, definitions, time points) within the outcome domain?**	No.	Y / PY / PN / N / NI
**5.3 ... multiple eligible analyses of the data?**	No.	Y / PY / PN / N / NI
**Risk-of-bias judgement**	Low risk of bias.	Low / High / Some concerns
Optional: What is the predicted direction of bias due to selection of the reported result?	NA.	NA / Favours experimental / Favours comparator / Towards null /Away from null / Unpredictable

Supplementary file

Overall risk of bias

**Risk-of-bias judgement**	Some concerns.	Low / High / Some concerns
Optional: What is the overall predicted direction of bias for this outcome?	NA.	NA / Favours experimental / Favours comparator / Towards null /Away from null / Unpredictable

Supplementary file

Study details

Reference

Somers TJ, Kelleher SA, Dorfman CS, Shelby RA, Fisher HM, Nichols KR, et al. An mHealth Pain Coping Skills Training Intervention for Hematopoietic Stem Cell Transplantation Patients: Development and Pilot Randomized Controlled Trial. JMIR Mhealth Uhealth. 2018;6(3):e66.

Study design

X Individually-randomized parallel-group trial
☐ Cluster-randomized parallel-group trial
☐ Individually randomized cross-over (or other matched) trial

For the purposes of this assessment, the interventions being compared are defined as

Experimental: Pain coping skills training (PCST) Comparator: TAU

**Specify which outcome is being assessed for risk of bias**	Pain severity
**Specify the numerical result being assessed.** In case of multiple alternative analyses being presented, specify the numeric result (e.g. RR = 1.52 (95% CI 0.83 to 2.77) and/or a reference (e.g. to a table, figure or paragraph) that uniquely defines the result being assessed.	"The pattern of effect sizes suggests that individuals in the intervention group showed greater improvements in pain disability (d=0.79 vs 0.69)."

Is the review team's aim for this result…?

X to assess the effect of *assignment to intervention* (the 'intention-to-treat' effect)
☐ to assess the effect of *adhering to intervention* (the 'per-protocol' effect)

If the aim is to assess the effect of *adhering to intervention*, select the deviations from intended intervention that should be addressed (at least one must be checked):

☐ occurrence of non-protocol interventions
☐ failures in implementing the intervention that could have affected the outcome
☐ non-adherence to their assigned intervention by trial participants

Which of the following sources were <u>obtained</u> to help inform the risk-of-bias assessment? (tick as many as apply)

Supplementary file

X	Journal article(s) with results of the trial
☐	Trial protocol
☐	Statistical analysis plan (SAP)
X	Non-commercial trial registry record (e.g. ClinicalTrials.gov record)
☐	Company-owned trial registry record (e.g. GSK Clinical Study Register record)
☐	"Grey literature" (e.g. unpublished thesis)
☐	Conference abstract(s) about the trial
☐	Regulatory document (e.g. Clinical Study Report, Drug Approval Package)
☐	Research ethics application
☐	Grant database summary (e.g. NIH RePORTER or Research Councils UK Gateway to Research)
☐	Personal communication with trialist
☐	Personal communication with the sponsor

Supplementary file

Risk of bias assessment

Responses underlined in green are potential markers for low risk of bias, and responses in red are potential markers for a risk of bias. Where questions relate only to sign posts to other questions, no formatting is used.

Domain 1: Risk of bias arising from the randomization process

Signalling questions	Comments	Response options
**1.1 Was the allocation sequence random?**	No information.	Y / PY / PN / N / NI
**1.2 Was the allocation sequence concealed until participants were enrolled and assigned to interventions?**	No information.	Y / PY / PN / N / NI
**1.3 Did baseline differences between intervention groups suggest a problem with the randomization process?**	No.	Y / PY / PN / N / NI
**Risk-of-bias judgement**	Some concerns.	Low / High / Some concerns
Optional: What is the predicted direction of bias arising from the randomization process?	Unpredictable.	NA / Favours experimental / Favours comparator / Towards null /Away from null / Unpredictable

Supplementary file

Domain 2: Risk of bias due to deviations from the intended interventions (*effect of assignment to intervention*)

Signalling questions	Comments	Response options
**2.1. Were participants aware of their assigned intervention during the trial?**	Yes.	Y / PY / PN / N / NI
**2.2. Were carers and people delivering the interventions aware of participants' assigned intervention during the trial?**	Yes.	Y / PY / PN / N / NI
**2.3. If Y/PY/NI to 2.1 or 2.2: Were there deviations from the intended intervention that arose because of the trial context?**	Probably no.	NA / Y / PY / PN / N / NI
**2.4 If Y/PY to 2.3: Were these deviations likely to have affected the outcome?**	NA.	NA / Y / PY / PN / N / NI
**2.5. If Y/PY/NI to 2.4: Were these deviations from intended intervention balanced between groups?**	NA.	NA / Y / PY / PN / N / NI
**2.6 Was an appropriate analysis used to estimate the effect of assignment to intervention?**	Yes. ITT analysis was conducted.	Y / PY / PN / N / NI
**2.7 If N/PN/NI to 2.6: Was there potential for a substantial impact (on the result) of the failure to analyse participants in the group to which they were randomized?**	NA.	NA / Y / PY / PN / N / NI
**Risk-of-bias judgement**	Low risk of bias.	Low / High / Some concerns
Optional: What is the predicted direction of bias due to deviations from intended interventions?	Away from null.	NA / Favours experimental / Favours comparator / Towards null /Away from null / Unpredictable

Supplementary file

Domain 3: Missing outcome data

Signalling questions	Comments	Response options
**3.1 Were data for this outcome available for all, or nearly all, participants randomized?**	Probably no. Three patients (83.3%, n=3/36), who never received assigned treatment, lost to follow-up. Reasons for losses were disclosed. Losses unlikely to affect final results.	Y / PY / PN / N / NI
**3.2 If N/PN/NI to 3.1: Is there evidence that the result was not biased by missing outcome data?**	No.	NA / Y / PY / PN / N
**3.3 If N/PN to 3.2: Could missingness in the outcome depend on its true value?**	Probably no.<br><br>No. The number of dropouts between the groups was equal.	NA / Y / PY / PN / N / NI
**3.4 If Y/PY/NI to 3.3: Is it likely that missingness in the outcome depended on its true value?**		NA / Y / PY / PN / N / NI
**Risk-of-bias judgement**	Low risk of bias.	Low / High / Some concerns
Optional: What is the predicted direction of bias due to missing outcome data?	NA.	NA / Favours experimental / Favours comparator / Towards null /Away from null / Unpredictable

Supplementary file

Domain 4: Risk of bias in measurement of the outcome

Signalling questions	Comments	Response options
**4.1 Was the method of measuring the outcome inappropriate?**	No.	Y / PY / PN / N / NI
**4.2 Could measurement or ascertainment of the outcome have differed between intervention groups?**	No.	Y / PY / PN / N / NI
**4.3 If N/PN/NI to 4.1 and 4.2: Were outcome assessors aware of the intervention received by study participants?**	Yes.	NA / Y / PY / PN / N / NI
**4.4 If Y/PY/NI to 4.3: Could assessment of the outcome have been influenced by knowledge of intervention received?**	Probably no. Since pain severity is a proven self-assessment measure of reliability and validity, knowledge of interventions is unlikely to affect outcomes.	NA / Y / PY / PN / N / NI
**4.5 If Y/PY/NI to 4.4: Is it likely that assessment of the outcome was influenced by knowledge of intervention received?**	NA.	NA / Y / PY / PN / N / NI
**Risk-of-bias judgement**	Low risk of bias.	Low / High / Some concerns
Optional: What is the predicted direction of bias in measurement of the outcome?	NA.	NA / Favours experimental / Favours comparator / Towards null /Away from null / Unpredictable

Supplementary file

Domain 5: Risk of bias in selection of the reported result

Signalling questions	Comments	Response options
**5.1 Were the data that produced this result analysed in accordance with a pre-specified analysis plan that was finalized before unblinded outcome data were available for analysis?**	Yes. ClinicalTrials.gov NCT01984671; https://clinicaltrials.gov/ct2/show/NCT01984671 (Archived by WebCite at http://www.webcitation.org/6xbpx3clZ).	Y / PY / PN / N / NI
**Is the numerical result being assessed likely to have been selected, on the basis of the results, from...**		
**5.2. ... multiple eligible outcome measurements (e.g. scales, definitions, time points) within the outcome domain?**	No.	Y / PY / PN / N / NI
**5.3 ... multiple eligible analyses of the data?**	No.	Y / PY / PN / N / NI
**Risk-of-bias judgement**	Low risk of bias.	Low / High / Some concerns
Optional: What is the predicted direction of bias due to selection of the reported result?	NA.	NA / Favours experimental / Favours comparator / Towards null /Away from null / Unpredictable

Supplementary file

Overall risk of bias

**Risk-of-bias judgement**	Some concerns.	Low / High / Some concerns
Optional: What is the overall predicted direction of bias for this outcome?	Unpredictable.	NA / Favours experimental / Favours comparator / Towards null /Away from null / Unpredictable

Supplementary file

Study details

**Reference**	Vogel PA, Solem S, Hagen K, Moen EM, Launes G, Håland Å, et al. A pilot randomized controlled trial of videoconference-assisted treatment for obsessive-compulsive disorder. Behav Res Ther. 2014;63:162-168.

Study design

X Individually-randomized parallel-group trial
☐ Cluster-randomized parallel-group trial
☐ Individually randomized cross-over (or other matched) trial

For the purposes of this assessment, the interventions being compared are defined as

Experimental:	Videoconference-assisted exposure and response prevention (VERP)	Comparator:	WLC

**Specify which outcome is being assessed for risk of bias**	Yale-Brown Obsessive-Compulsive Scale (Y-BOCS)
**Specify the numerical result being assessed.** In case of multiple alternative analyses being presented, specify the numeric result (e.g. RR = 1.52 (95% CI 0.83 to 2.77) and/or a reference (e.g. to a table, figure or paragraph) that uniquely defines the result being assessed.	"On the primary outcome measure (Y-BOCS) there was a significant difference between the conditions, $F(2, 27) = 7.8$, $p = .002$."

Is the review team's aim for this result…?

X to assess the effect of *assignment to intervention* (the 'intention-to-treat' effect)
☐ to assess the effect of *adhering to intervention* (the 'per-protocol' effect)

If the aim is to assess the effect of *adhering to intervention*, select the deviations from intended intervention that should be addressed (at least one must be checked):

☐ occurrence of non-protocol interventions
☐ failures in implementing the intervention that could have affected the outcome

☐	non-adherence to their assigned intervention by trial participants

Which of the following sources were obtained to help inform the risk-of-bias assessment? (tick as many as apply)

X	Journal article(s) with results of the trial
☐	Trial protocol
☐	Statistical analysis plan (SAP)
☐	Non-commercial trial registry record (e.g. ClinicalTrials.gov record)
☐	Company-owned trial registry record (e.g. GSK Clinical Study Register record)
☐	"Grey literature" (e.g. unpublished thesis)
☐	Conference abstract(s) about the trial
☐	Regulatory document (e.g. Clinical Study Report, Drug Approval Package)
☐	Research ethics application
☐	Grant database summary (e.g. NIH RePORTER or Research Councils UK Gateway to Research)
☐	Personal communication with trialist
☐	Personal communication with the sponsor

Supplementary file

Risk of bias assessment

Responses underlined in green are potential markers for low risk of bias, and responses in red are potential markers for a risk of bias. Where questions relate only to sign posts to other questions, no formatting is used.

Domain 1: Risk of bias arising from the randomization process

Signalling questions	Comments	Response options
**1.1 Was the allocation sequence random?**	No information.	Y / PY / PN / N / NI
**1.2 Was the allocation sequence concealed until participants were enrolled and assigned to interventions?**	No information.	Y / PY / PN / N / NI
**1.3 Did baseline differences between intervention groups suggest a problem with the randomization process?**	Yes. "There was a significant difference in the three conditions with regard to age and number of children."	Y / PY / PN / N / NI
**Risk-of-bias judgement**	High risk of bias.	Low / High / Some concerns
Optional: What is the predicted direction of bias arising from the randomization process?	Unpredictable.	NA / Favours experimental / Favours comparator / Towards null /Away from null / Unpredictable

Supplementary file

Domain 2: Risk of bias due to deviations from the intended interventions (*effect of assignment to intervention*)

Signalling questions	Comments	Response options
**2.1. Were participants aware of their assigned intervention during the trial?**	No information.	Y / PY / PN / N / NI
**2.2. Were carers and people delivering the interventions aware of participants' assigned intervention during the trial?**	Yes.	Y / PY / PN / N / NI
**2.3. If Y/PY/NI to 2.1 or 2.2: Were there deviations from the intended intervention that arose because of the trial context?**	Probably no.	NA / Y / PY / PN / N / NI
**2.4 If Y/PY to 2.3: Were these deviations likely to have affected the outcome?**	NA.	NA / Y / PY / PN / N / NI
**2.5. If Y/PY/NI to 2.4: Were these deviations from intended intervention balanced between groups?**	NA.	NA / Y / PY / PN / N / NI
**2.6 Was an appropriate analysis used to estimate the effect of assignment to intervention?**	Probably no.	Y / PY / PN / N / NI
**2.7 If N/PN/NI to 2.6: Was there potential for a substantial impact (on the result) of the failure to analyse participants in the group to which they were randomized?**	No information.	NA / Y / PY / PN / N / NI
**Risk-of-bias judgement**	Hight risk of bias.	Low / High / Some concerns
Optional: What is the predicted direction of bias due to deviations from intended interventions?	Unpredictable.	NA / Favours experimental / Favours comparator / Towards null /Away from null / Unpredictable

Supplementary file

Domain 3: Missing outcome data

Signalling questions	Comments	Response options
**3.1 Were data for this outcome available for all, or nearly all, participants randomized?**	Probably no. 10% (n = 2/20) of the participants were dropped out.	Y / PY / PN / N / NI
**3.2 If N/PN/NI to 3.1: Is there evidence that the result was not biased by missing outcome data?**	No.	NA / Y / PY / PN / N
**3.3 If N/PN to 3.2: Could missingness in the outcome depend on its true value?**	Probably no. Only two people in the waiting group were dropped out.	NA / Y / PY / PN / N / NI
**3.4 If Y/PY/NI to 3.3: Is it likely that missingness in the outcome depended on its true value?**	NA.	NA / Y / PY / PN / N / NI
**Risk-of-bias judgement**	Low risk of bias.	Low / High / Some concerns
Optional: What is the predicted direction of bias due to missing outcome data?	NA.	NA / Favours experimental / Favours comparator / Towards null /Away from null / Unpredictable

Supplementary file

Domain 4: Risk of bias in measurement of the outcome

Signalling questions	Comments	Response options
**4.1 Was the method of measuring the outcome inappropriate?**	No information.	Y / PY / PN / N / NI
**4.2 Could measurement or ascertainment of the outcome have differed between intervention groups?**	No.	Y / PY / PN / N / NI
**4.3 If N/PN/NI to 4.1 and 4.2: Were outcome assessors aware of the intervention received by study participants?**	Yes.	NA / Y / PY / PN / N / NI
**4.4 If Y/PY/NI to 4.3: Could assessment of the outcome have been influenced by knowledge of intervention received?**	No information.	NA / Y / PY / PN / N / NI
**4.5 If Y/PY/NI to 4.4: Is it likely that assessment of the outcome was influenced by knowledge of intervention received?**	Probably no. Since Y-BOCS is a proven self-assessment measure of reliability and validity, knowledge of interventions is unlikely to affect outcomes.	NA / Y / PY / PN / N / NI
**Risk-of-bias judgement**	Some concerns.	Low / High / Some concerns
Optional: What is the predicted direction of bias in measurement of the outcome?	Unpredictable.	NA / Favours experimental / Favours comparator / Towards null /Away from null / Unpredictable

Supplementary file

Domain 5: Risk of bias in selection of the reported result

Signalling questions	Comments	Response options
**5.1 Were the data that produced this result analysed in accordance with a pre-specified analysis plan that was finalized before unblinded outcome data were available for analysis?**	No information.	Y / PY / PN / N / NI
**Is the numerical result being assessed likely to have been selected, on the basis of the results, from...**		
**5.2. ... multiple eligible outcome measurements (e.g. scales, definitions, time points) within the outcome domain?**	No information.	Y / PY / PN / N / NI
**5.3 ... multiple eligible analyses of the data?**	No information.	Y / PY / PN / N / NI
**Risk-of-bias judgement**	Some concerns.	Low / High / Some concerns
Optional: What is the predicted direction of bias due to selection of the reported result?	Unpredictable.	NA / Favours experimental / Favours comparator / Towards null /Away from null / Unpredictable

Supplementary file

Overall risk of bias

**Risk-of-bias judgement**	High risk of bias.	Low / High / Some concerns
Optional: What is the overall predicted direction of bias for this outcome?	Unpredictable.	NA / Favours experimental / Favours comparator / Towards null /Away from null / Unpredictable

Supplementary file

Study details

**Reference**	Vranceanu AM, Jacobs C, Lin A, Greenberg J, Funes CJ, Harris MB, et al. Results of a feasibility randomized controlled trial (RCT) of the Toolkit for Optimal Recovery (TOR): a live video program to prevent chronic pain in at-risk adults with orthopedic injuries. Pilot Feasibility Stud. 2019;5:30.

Study design

X Individually-randomized parallel-group trial
☐ Cluster-randomized parallel-group trial
☐ Individually randomized cross-over (or other matched) trial

For the purposes of this assessment, the interventions being compared are defined as

Experimental: VCBT Comparator: TAU

**Specify which outcome is being assessed for risk of bias**	Physical function in short musculoskeletal function assessment questionnaire (SMFA)
**Specify the numerical result being assessed.** In case of multiple alternative analyses being presented, specify the numeric result (e.g. RR = 1.52 (95% CI 0.83 to 2.77) and/or a reference (e.g. to a table, figure or paragraph) that uniquely defines the result being assessed.	Within-subject effect size for improvement from baseline to post-test in TOR was large for SMFA (d = 2.7).

Is the review team's aim for this result…?

X to assess the effect of *assignment to intervention* (the 'intention-to-treat' effect)
☐ to assess the effect of *adhering to intervention* (the 'per-protocol' effect)

If the aim is to assess the effect of *adhering to intervention*, select the deviations from intended intervention that should be addressed (at least one must be checked):

☐ occurrence of non-protocol interventions
☐ failures in implementing the intervention that could have affected the outcome
☐ non-adherence to their assigned intervention by trial participants

Supplementary file

**Which of the following sources were <u>obtained</u> to help inform the risk-of-bias assessment? (tick as many as apply)**<br>X Journal article(s) with results of the trial<br>☐ Trial protocol<br>☐ Statistical analysis plan (SAP)<br>X Non-commercial trial registry record (e.g. ClinicalTrials.gov record)<br>☐ Company-owned trial registry record (e.g. GSK Clinical Study Register record)<br>☐ “Grey literature” (e.g. unpublished thesis)<br>☐ Conference abstract(s) about the trial<br>☐ Regulatory document (e.g. Clinical Study Report, Drug Approval Package)<br>☐ Research ethics application<br>☐ Grant database summary (e.g. NIH RePORTER or Research Councils UK Gateway to Research)<br>☐ Personal communication with trialist<br>☐ Personal communication with the sponsor

Supplementary file

Risk of bias assessment

Responses underlined in green are potential markers for low risk of bias, and responses in red are potential markers for a risk of bias. Where questions relate only to sign posts to other questions, no formatting is used.

Domain 1: Risk of bias arising from the randomization process

Signalling questions	Comments	Response options
**1.1 Was the allocation sequence random?**	Yes. "Randomization was performed using a random number generator to maintain balance between the groups."	Y / PY / PN / N / NI
**1.2 Was the allocation sequence concealed until participants were enrolled and assigned to interventions?**	Yes. "Surgeons who referred participants as well as the PI were blind to intervention and control."	Y / PY / PN / N / NI
**1.3 Did baseline differences between intervention groups suggest a problem with the randomization process?**	No.	Y / PY / PN / N / NI
**Risk-of-bias judgement**	Low risk of bias.	Low / High / Some concerns
Optional: What is the predicted direction of bias arising from the randomization process?	NA.	NA / Favours experimental / Favours comparator / Towards null /Away from null / Unpredictable

Supplementary file

Domain 2: Risk of bias due to deviations from the intended interventions (*effect of assignment to intervention*)

Signalling questions	Comments	Response options
**2.1. Were participants aware of their assigned intervention during the trial?**	Yes.	Y / PY / PN / N / NI
**2.2. Were carers and people delivering the interventions aware of participants' assigned intervention during the trial?**	Yes. "Because we compared TOR with UC, neither the patient nor the therapist was blinded."	Y / PY / PN / N / NI
**2.3. If Y/PY/NI to 2.1 or 2.2: Were there deviations from the intended intervention that arose because of the trial context?**	Probably no.	NA / Y / PY / PN / N / NI
**2.4 If Y/PY to 2.3: Were these deviations likely to have affected the outcome?**	NA.	NA / Y / PY / PN / N / NI
**2.5. If Y/PY/NI to 2.4: Were these deviations from intended intervention balanced between groups?**	No. "There were no differences in any of the baseline characteristics between follow-up status (trial completers versus lost to follow up) (p > .05)."	NA / Y / PY / PN / N / NI
**2.6 Was an appropriate analysis used to estimate the effect of assignment to intervention?**	No.	Y / PY / PN / N / NI
**2.7 If N/PN/NI to 2.6: Was there potential for a substantial impact (on the result) of the failure to analyse participants in the group to which they were randomized?**	No.	NA / Y / PY / PN / N / NI
**Risk-of-bias judgement**	Some concerns.	Low / High / Some concerns
Optional: What is the predicted direction of bias due to deviations from intended interventions?	Unpredictable.	NA / Favours experimental / Favours comparator / Towards null /Away from null / Unpredictable

Supplementary file

Domain 3: Missing outcome data

Signalling questions	Comments	Response options
**3.1 Were data for this outcome available for all, or nearly all, participants randomized?**	Yes. 96.3% (n = 52/54) of the participants submitted all the data.	Y / PY / PN / N / NI
**3.2 If N/PN/NI to 3.1: Is there evidence that the result was not biased by missing outcome data?**	NA.	NA / Y / PY / PN / N
**3.3 If N/PN to 3.2: Could missingness in the outcome depend on its true value?**	NA.<br><br>NA.	NA / Y / PY / PN / N / NI
**3.4 If Y/PY/NI to 3.3: Is it likely that missingness in the outcome depended on its true value?**		NA / Y / PY / PN / N / NI
**Risk-of-bias judgement**	Low risk of bias.	Low / High / Some concerns
Optional: What is the predicted direction of bias due to missing outcome data?	NA.	NA / Favours experimental / Favours comparator / Towards null /Away from null / Unpredictable

Supplementary file

Domain 4: Risk of bias in measurement of the outcome

Signalling questions	Comments	Response options
**4.1 Was the method of measuring the outcome inappropriate?**	No.	Y / PY / PN / N / NI
**4.2 Could measurement or ascertainment of the outcome have differed between intervention groups?**	No.	Y / PY / PN / N / NI
**4.3 If N/PN/NI to 4.1 and 4.2: Were outcome assessors aware of the intervention received by study participants?**	Probably no.	NA / Y / PY / PN / N / NI
**4.4 If Y/PY/NI to 4.3: Could assessment of the outcome have been influenced by knowledge of intervention received?**	NA.	NA / Y / PY / PN / N / NI
**4.5 If Y/PY/NI to 4.4: Is it likely that assessment of the outcome was influenced by knowledge of intervention received?**	NA.	NA / Y / PY / PN / N / NI
**Risk-of-bias judgement**	Low risk of bias.	Low / High / Some concerns
Optional: What is the predicted direction of bias in measurement of the outcome?	Na.	NA / Favours experimental / Favours comparator / Towards null /Away from null / Unpredictable

Supplementary file

Domain 5: Risk of bias in selection of the reported result

Signalling questions	Comments	Response options
**5.1 Were the data that produced this result analysed in accordance with a pre-specified analysis plan that was finalized before unblinded outcome data were available for analysis?**	Yes. ClinicalTrials.gov ID: NCT03405610. Registered on January 28, 2018—retrospectively registered.	Y / PY / PN / N / NI
**Is the numerical result being assessed likely to have been selected, on the basis of the results, from...**		
**5.2. ... multiple eligible outcome measurements (e.g. scales, definitions, time points) within the outcome domain?**	No.	Y / PY / PN / N / NI
**5.3 ... multiple eligible analyses of the data?**	No.	Y / PY / PN / N / NI
**Risk-of-bias judgement**	Low risk of bias.	Low / High / Some concerns
Optional: What is the predicted direction of bias due to selection of the reported result?	NA.	NA / Favours experimental / Favours comparator / Towards null /Away from null / Unpredictable

Overall risk of bias

**Risk-of-bias judgement**	Some concerns.	Low / High / Some concerns
Optional: What is the overall predicted direction of bias for this outcome?	Unpredictable.	NA / Favours experimental / Favours comparator / Towards null /Away from null / Unpredictable

This work is licensed under a Creative Commons Attribution-NonCommercial-NoDerivatives 4.0 International License.

Supplementary file

Supplementary file 4.
Table S2. List of excluded studies

No.	Study	Reason for exclusions
1	Storch EA, Salloum A, King MA, et al. A RANDOMIZED CONTROLLED TRIAL IN COMMUNITY MENTAL HEALTH CENTERS OF COMPUTER-ASSISTED COGNITIVE BEHAVIORAL THERAPY VERSUS TREATMENT AS USUAL FOR CHILDREN WITH ANXIETY. *Depress Anxiety*. 2015;32(11):843-852. doi:10.1002/da.22399	Age < 18 years old
2	Wood JJ, Ehrenreich-May J, Alessandri M, et al. Cognitive behavioral therapy for early adolescents with autism spectrum disorders and clinical anxiety: a randomized, controlled trial. Behav Ther. 2015;46(1):7-19. doi:10.1016/j.beth.2014.01.002	Not VCBT
3	Elkins RM, Gallo KP, Pincus DB, Comer JS. Moderators of intensive CBT for adolescent panic disorder: the of fear and avoidance. *Child Adolesc Ment Health*. 2016;21(1):30-36. doi:10.1111/camh.12122	Not VCBT
4	Eaton LH, Godfrey DS, Langford DJ, Rue T, Tauben DJ, Doorenbos AZ. Telementoring for improving primary care provider knowledge and competence in managing chronic pain: A randomised controlled trial. *J Telemed Telecare*. 2020;26(1-2):21-27. doi:10.1177/1357633X18802978	Not VCBT
5	Hungerbuethler I, Leite RFM, van de Bilt MT, Gattaz W. A randomized clinical trial of home-based telepsychiatric outpatient care via videoconferencing: design, methodology, and implementation. Archive of Clinical Psychiatry. 2015;42(3):76-78.	Not meet control criteria
6	Bray VJ, Dhillon HM, Bell ML, et al. Evaluation of a Web-Based Cognitive Rehabilitation Program in Cancer Survivors Reporting Cognitive Symptoms After Chemotherapy. *J Clin Oncol*. 2017;35(2):217-225. doi:10.1200/JCO.2016.67.8201	Not VCBT
7	Nelson EL, Barnard M, Cain S. Treating childhood depression over videoconferencing. *Telemed J E Health*. 2003;9(1):49-55. doi:10.1089/153056203763317648	Age < 18 years old
8	O'Neil A, Taylor B, Sanderson K, et al. Efficacy and feasibility of a tele-health intervention for acute coronary syndrome patients with depression: results of the "MoodCare" randomized controlled trial. *Ann Behav Med*. 2014;48(2):163-174. doi:10.1007/s12160-014-9592-0	Not VCBT
9	Eaton LH, Gordon DB, Wyant S, et al. Development and implementation of a telehealth-enhanced intervention for pain and symptom management. *Contemp Clin Trials*. 2014;38(2):213-220. doi:10.1016/j.cct.2014.05.005	Not VCBT
10	van Rosmalen-Nooijens K, Lo Fo Wong S, Prins J, Lagro-Janssen T. Young People, Adult Worries: Randomized Controlled Trial and Feasibility Study of the Internet-Based Self-Support Method "Feel the ViBe" for Adolescents and Young Adults Exposed to Family Violence. *J Med Internet Res*. 2017;19(6):e204. Published 2017 Jun 12. doi:10.2196/jmir.6004	Not VCBT
11	Hilyard A, Kingsley J, Sommerfield D, Taylor S, Bear N, Gibson N. Feasibility of a Randomized Controlled Trial of Paediatric Interdisciplinary Pain Management Using Home-Based Telehealth. *J Pain Res*. 2020;13:897-908. Published 2020 May 1. doi:10.2147/JPR.S217022	Not VCBT
12	Milbury K, Li Y, Durrani S, et al. A Mindfulness-Based Intervention as a Supportive Care Strategy for Patients with Metastatic Non-Small Cell Lung Cancer and Their Spouses: Results of a Three-Arm Pilot Randomized Controlled Trial. *Oncologist*. 2020;25(11):e1794-e1802. doi:10.1634/theoncologist.2020-0125	Not VCBT
13	Washington KT, Demiris G, Parker Oliver D, Albright DL, Craig KW, Tatum P. Delivering problem-solving therapy to family caregivers of	Not VCBT

	people with cancer: A feasibility study in outpatient palliative care. *Psychooncology*. 2018;27(10):2494-2499. doi:10.1002/pon.4859	
14	Freedenberg VA, Hinds PS, Friedmann E. Mindfulness-Based Stress Reduction and Group Support Decrease Stress in Adolescents with Cardiac Diagnoses: A Randomized Two-Group Study. *Pediatr Cardiol*. 2017;38(7):1415-1425. doi:10.1007/s00246-017-1679-5	Not VCBT
15	Yeung A, Martinson MA, Baer L, et al. The Effectiveness of Telepsychiatry-Based Culturally Sensitive Collaborative Treatment for Depressed Chinese American Immigrants: A Randomized Controlled Trial. *J Clin Psychiatry*. 2016;77(8):e996-e1002. doi:10.4088/JCP.15m09952	Not VCBT
16	Cassin S, Leung S, Hawa R, Wnuk S, Jackson T, Sockalingam S. Food Addiction Is Associated with Binge Eating and Psychiatric Distress among Post-Operative Bariatric Surgery Patients and May Improve in Response to Cognitive Behavioural Therapy. *Nutrients*. 2020;12(10):2905. Published 2020 Sep 23. doi:10.3390/nu12102905	Not VCBT
17	Chang MW, Brown R, Nitzke S. Participant recruitment and retention in a pilot program to prevent weight gain in low-income overweight and obese mothers. *BMC Public Health*. 2009;9:424. Published 2009 Nov 21. doi:10.1186/1471-2458-9-424	Not VCBT
18	Garland EL, Hanley AW, Kline A, Cooperman NA. Mindfulness-Oriented Recovery Enhancement reduces opioid craving among individuals with opioid use disorder and chronic pain in medication assisted treatment: Ecological momentary assessments from a stage 1 randomized controlled trial. *Drug Alcohol Depend*. 2019;203:61-65. doi:10.1016/j.drugalcdep.2019.07.007	Not VCBT
20	Demiris G, Oliver DP, Washington K, et al. A Problem Solving Intervention for hospice caregivers: a pilot study. *J Palliat Med*. 2010;13(8):1005-1011. doi:10.1089/jpm.2010.0022	Not meet control criteria
21	Khan F, Granville N, Malkani R, Chathampally Y. Health-Related Quality of Life Improvements in Systemic Lupus Erythematosus Derived from a Digital Therapeutic Plus Tele-Health Coaching Intervention: Randomized Controlled Pilot Trial. *J Med Internet Res*. 2020;22(10):e23868. Published 2020 Oct 20. doi:10.2196/23868	Not VCBT
22	Moreno FA, Chong J, Dumbauld J, Humke M, Byreddy S. Use of standard Webcam and Internet equipment for telepsychiatry treatment of depression among underserved Hispanics. *Psychiatr Serv*. 2012;63(12):1213-1217. doi:10.1176/appi.ps.201100274	Not VCBT
23	Fortney JC, Pyne JM, Mouden SB, et al. Practice-based versus telemedicine-based collaborative care for depression in rural federally qualified health centers: a pragmatic randomized comparative effectiveness trial. *Am J Psychiatry*. 2013;170(4):414-425. doi:10.1176/appi.ajp.2012.12050696	Not VCBT
24	Schliep M, Chudy-Onwugaje K, Abutaleb A, et al. TELEmedicine for Patients With Inflammatory Bowel Disease (TELE-IBD) Does Not Improve Depressive Symptoms or General Quality of Life Compared With Standard Care at Tertiary Referral Centers. *Crohns Colitis 360*. 2020;2(1):otaa002. doi:10.1093/crocol/otaa002	Not VCBT
25	Eilenberg T, Fink P, Jensen J, Rief W, Frostholm L. Acceptance and commitment group therapy (ACT-G) for health anxiety: A randomized controlled trial. *Psychological Medicine,2020;46*(1): 103-115. doi:10.1017/S0033291715001579	Not VCBT
26	Cederberg JT, Cernvall M, Dahl J, von Essen L, Ljungman G. Acceptance as a Mediator for Change in Acceptance and Commitment Therapy for Persons with Chronic Pain?. *Int J Behav Med*. 2016;23(1):21-29. doi:10.1007/s12529-015-9494-y	Not VCBT
27	Auslander W, McGinnis H, Tlapek S, et al. Adaptation and implementation of a trauma-focused cognitive behavioral intervention for girls in child welfare. *Am J Orthopsychiatry*. 2017;87(3):206-215. doi:10.1037/ort0000233	Age < 18 years old
28	Wagner AW, Jakupcak M, Kowalski HM, Bittinger JN, Golshan S. Behavioral Activation as a Treatment for Posttraumatic Stress Disorder	Not VCBT

	Among Returning Veterans: A Randomized Trial. *Psychiatr Serv*. 2019;70(10):867-873. doi:10.1176/appi.ps.201800572	
29	Nyström MBT, Stenling A, Sjöström E, et al. Behavioral activation versus physical activity via the internet: A randomized controlled trial. *J Affect Disord*. 2017;215:85-93. doi:10.1016/j.jad.2017.03.018	Not VCBT
30	Verdurmen MJ, Videler AC, Kamperman AM, Khasho D, van der Feltz-Cornelis CM. Cognitive behavioral therapy for somatic symptom disorders in later life: a prospective comparative explorative pilot study in two clinical populations. Neuropsychiatr Dis Treat. 2017;13:2331-2339. Published 2017 Sep 1. doi:10.2147/NDT.S141208	Not VCBT
31	Hedman E, Andersson E, Lindefors N, Andersson G, Rück C, Ljótsson B. Cost-effectiveness and long-term effectiveness of internet-based cognitive behaviour therapy for severe health anxiety. *Psychol Med*. 2013;43(2):363-374. doi:10.1017/S0033291712001079	Not VCBT
32	Wada SL, Carey J, Wolfe CR. An Online Family Intervention to Reduce Parental Distress Following Pediatric Brain Injury. *Journal of Consulting and Clinical Psychology*. 2006;74(5):445-454.	Not VCBT
33	Calvano C, Groß M, Warschburger P. Do Mothers Benefit from a Child-Focused Cognitive Behavioral Treatment (CBT) for Childhood Functional Abdominal Pain? A Randomized Controlled Pilot Trial. *Children (Basel)*. 2017;4(2):13. Published 2017 Feb 15. doi:10.3390/children4020013	Age < 18 years old
34	Fichter MM, Quadflieg N, Nisslmüller K, et al. Does internet-based prevention reduce the risk of relapse for anorexia nervosa?. *Behav Res Ther*. 2012;50(3):180-190. doi:10.1016/j.brat.2011.12.003	Not VCBT
35	Rosas LG, Azar KMJ, Lv N, et al. Effect of an Intervention for Obesity and Depression on Patient-Centered Outcomes: An RCT. *Am J Prev Med*. 2020;58(4):496-505. doi:10.1016/j.amepre.2019.11.005	Not VCBT
36	Stikkelbroek Y, Bodden DH, Deković M. et al. Effectiveness and cost effectiveness of cognitive behavioral therapy (CBT) in clinically depressed adolescents: individual CBT versus treatment as usual (TAU). BMC Psychiatry 2013;13:314. https://doi.org/10.1186/1471-244X-13-314	Not VCBT
37	Müller M, Matthies LM, Goetz M. *et al.* Effectiveness and cost-effectiveness of an electronic mindfulness-based intervention (eMBI) on maternal mental health during pregnancy: the *mindmom* study protocol for a randomized controlled clinical trial. *Trials. 2020;*21:933. https://doi.org/10.1186/s13063-020-04873-3	Not VCBT
38	Wade SL, Kurowski BG, Kirkwood MW, et al. Online problem-solving therapy after traumatic brain injury: a randomized controlled trial. *Pediatrics*. 2015;135(2):e487-e495. doi:10.1542/peds.2014-1386	Age < 18 years old
39	Lopez-Montoyo A, Quero S, Montero-Marin J. *et al.* Effectiveness of a brief psychological mindfulness-based intervention for the treatment of depression in primary care: study protocol for a randomized controlled clinical trial. *BMC Psychiatry* 2019;19:301. https://doi.org/10.1186/s12888-019-2298-x	Not RCT (protocol)
40	Gharraee B, Tajrishi KZ, Farani AR, Bolari J, Farahani H. THE EFFECTIVENESS OF ACCEPTANCE AND COMMITMENT THERAPY FOR SOCIAL ANXIETY DISORDER. *International Journal of Life Science and Pharma Research.* 2018;8(4): L1-L9.	Not VCBT
41	Seguranyes G, Costa D, Fuentelsaz-Gallego C, et al. Efficacy of a videoconferencing intervention compared with standard postnatal care at primary care health centres in Catalonia. *Midwifery*. 2014;30(6):764-771. doi:10.1016/j.midw.2013.08.004	Not VCBT
42	Sayal K, Roe J, Ball H. *et al.* Feasibility of a randomised controlled trial of remotely delivered problem-solving cognitive behaviour therapy versus usual care for young people with depression and repeat self-harm: lessons learnt (e-DASH). *BMC Psychiatry* 2019;19:42.	Data not available

	https://doi.org/10.1186/s12888-018-2005-3	
43	Hange D, Ariai N, Kivi M, Eriksson MC, Nejati S, Petersson EL. The impact of internet-based cognitive behavior therapy on work ability in patients with depression - a randomized controlled study. *Int J Gen Med*. 2017;10:151-159. Published 2017 May 19. doi:10.2147/IJGM.S129710	Not VCBT
44	Scogin F, Lichstein K, DiNapoli EA, et al. Effects of Integrated Telehealth-Delivered Cognitive-Behavioral Therapy for Depression and Insomnia in Rural Older Adults. *J Psychother Integr*. 2018;28(3):292-309. doi:10.1037/int0000121	Data not available
